# Sampling inequalities affect generalization of neuroimaging-based diagnostic classifiers in psychiatry

**DOI:** 10.1186/s12916-023-02941-4

**Published:** 2023-07-03

**Authors:** Zhiyi Chen, Bowen Hu, Xuerong Liu, Benjamin Becker, Simon B. Eickhoff, Kuan Miao, Xingmei Gu, Yancheng Tang, Xin Dai, Chao Li, Artemiy Leonov, Zhibing Xiao, Zhengzhi Feng, Ji Chen, Hu Chuan-Peng

**Affiliations:** 1 Experimental Research Center for Medical and Psychological Science (ERC-MPS), School of Psychology, Third Military Medical University, Chongqing, China; 2 Faculty of Psychology, Southwest University, Chongqing, China; 3 The Center of Psychosomatic Medicine, Sichuan Provincial Center for Mental Health, Sichuan Provincial People’s Hospital, Chengdu, China; 4 The Clinical Hospital of Chengdu Brain Science Institute, MOE Key Laboratory for Neuroinformation, University of Electronic Science and Technology of China, Chengdu, China; 5 Institute of Systems Neuroscience, Heinrich Heine University Düsseldorf, Düsseldorf, Germany; 6 School of Business and Management, Shanghai International Studies University, Shanghai, China; 7 Department of Radiology, The Third Affiliated Hospital, Sun Yat-Sen University, Guangdong, China; 8 School of Psychology, Clark University, Worcester, MA USA; 9 State Key Laboratory of Cognitive Neuroscience and Learning, Beijing Normal University, Beijing, China; 10 Department of Psychology and Behavioral Sciences, Zhejiang University, Hangzhou, China; 11 Department of Psychiatry, The Fourth Affiliated Hospital, Zhejiang University School of Medicine, Yiwu, Zhejiang China; 12 School of Psychology, Nanjing Normal University, Nanjing, China

**Keywords:** Psychiatric machine learning, Diagnostic classification, Meta-analysis, Neuroimaging, Sampling inequalities

## Abstract

**Background:**

The development of machine learning models for aiding in the diagnosis of mental disorder is recognized as a significant breakthrough in the field of psychiatry. However, clinical practice of such models remains a challenge, with poor generalizability being a major limitation.

**Methods:**

Here, we conducted a pre-registered meta-research assessment on neuroimaging-based models in the psychiatric literature, quantitatively examining global and regional sampling issues over recent decades, from a view that has been relatively underexplored. A total of 476 studies (*n* = 118,137) were included in the current assessment. Based on these findings, we built a comprehensive 5-star rating system to quantitatively evaluate the quality of existing machine learning models for psychiatric diagnoses.

**Results:**

A global sampling inequality in these models was revealed quantitatively (sampling Gini coefficient (*G*) = 0.81, *p* < .01), varying across different countries (regions) (e.g., China, *G* = 0.47; the USA, *G* = 0.58; Germany, *G* = 0.78; the UK, *G* = 0.87). Furthermore, the severity of this sampling inequality was significantly predicted by national economic levels (*β* =  − 2.75, *p* < .001, *R*^*2*^_*adj*_ = 0.40; *r* =  − .84, 95% CI: − .41 to − .97), and was plausibly predictable for model performance, with higher sampling inequality for reporting higher classification accuracy. Further analyses showed that lack of independent testing (84.24% of models, 95% CI: 81.0–87.5%), improper cross-validation (51.68% of models, 95% CI: 47.2–56.2%), and poor technical transparency (87.8% of models, 95% CI: 84.9–90.8%)/availability (80.88% of models, 95% CI: 77.3–84.4%) are prevailing in current diagnostic classifiers despite improvements over time. Relating to these observations, model performances were found decreased in studies with independent cross-country sampling validations (all *p* < .001, BF_10_ > 15). In light of this, we proposed a purpose-built quantitative assessment checklist, which demonstrated that the overall ratings of these models increased by publication year but were negatively associated with model performance.

**Conclusions:**

Together, improving sampling economic equality and hence the quality of machine learning models may be a crucial facet to plausibly translating neuroimaging-based diagnostic classifiers into clinical practice.

**Supplementary Information:**

The online version contains supplementary material available at 10.1186/s12916-023-02941-4.

## Background

Machine learning (ML) models have been extensively utilized for classifying patients with mental illness to aid in clinical decision-making [1, 2]. By building machine learning models that are trained from neuroimaging-based features, the diagnostic decision could be more accurate and reliable with the aid of these objective and high-dimensional biomarkers [3, 4]. Furthermore, given the multivariate nature of brain features, machine learning techniques could capture the whole neural pattern across high-volume dependent voxels for revealing pathophysiological signatures of these disorders, while individualized prediction of machine learning models in the neuroimaging-based ML models also facilitates to address the increasing needs of precision psychiatry [5, 6]. Despite considerable efforts devoted to this end, the translation of machine learning classification for diagnostic and treatment recommendation into clinical practice remains challenging [7]. This is partly due to the poor generalizability of particular these neuroimaging-based classifiers, which are often optimized within a specific sample to incur failure of generalizing to diagnose unseen patients in new samples [8–10]. Although these classifiers can be trained to achieve a desirably high accuracy in a specific cohort, they are not representative of a more general population across medical centers, geographic regions, socioeconomic statuses, and ethnic groups [11, 12]. Moreover, persisting concerns over generalizability imply potential sampling biases despite the substantially increased size of data over recent decades [13].

As promising noninvasive, in vivo techniques (e.g., magnetic resonance imaging, MRI; electroencephalogram, EEG; positron emission computed tomography, PET), they provide unique opportunities to assess brain structure, function, and metabolic anomalies for revealing the pathophysiological signatures of these psychiatric disorders as intermediate phenotype, and hence fueled the enthusiasm in these machine learning diagnostic models [9, 14]. In addition, with the huge developments of big-data sharing initiatives (e.g., UK Biobank, Alzheimer’s Disease Neuroimaging Initiative), the diagnostic studies utilizing neuroimaging-based methods for classifying psychiatric conditions have seen a remarkable proliferation at an unprecedented speed over the recent decades [9, 15]. Despite these technical merits and promising research insights, these approaches are nonetheless cost, somewhat non-scalable, and are mostly not readily available or accessible in low-income countries and regions, especially the high-field MRI and PET for neural system mapping. In this vein, probing into why and how the sampling bias and relevant factors impeded the generalizability could be a potent avenue prompting translations of these neuroimaging-based machine learning models into clinical actions. However, comprehensive knowledge about the degree of such sampling issues and what relevant factors incur poor generalizability in these models is still scarce.

The importance of replication in generalizing scientific conclusions has been increasingly stressed, and a “replication crisis” has been discussed for several decades within or beyond psychological science: multiple experimental findings fail to be replicated and generalized across populations and contexts [16, 17]. One possible underlying reason may be that the available data was primarily and predominantly drawn from WEIRD (western, educated, industrialized, rich, and democratic) societies, which mirrors a typical sampling bias [18, 19]. Specifically, in 2008, 96% studies on human behavior relied on samples from WEIRD counties, with the remaining 82% of global population being largely ignored [20, 21]. Recently, we have conducted a systematic appraisal for neuroimaging-based machine learning models in the psychiatric diagnosis by using PTOBAST (Prediction model Risk Of Bias ASsessment Tool) criterion. Results demonstrated that 83.1% of these models are at high risk of bias (ROB), and further indicate a biased distribution of sampled populations [22]. Despite these descriptive evidences, there have been no quantitative analyses conducted to clearly illustrate the extent of sampling biases at a global or regional level [22]. And what’s more, the long-lasting discussion regarding the association between the regional economic level and these sampling biases remains uncertain, and requires reliable statistical evidence for clarification [22, 23]. Examining the status quo of sampling biases is particularly important for psychiatric neuroimaging-based classifiers as generalizability is critical for translating models into clinical actions [23, 24]. Patient groups, compared with non-clinical or healthy entities, are far more heterogeneous due to high inter-individual variability in psychopathology [25, 26]. This is affected not only by genetics, but environment, a broad sense covering socioeconomic status, family susceptibility, and living environment [27, 28]. Therefore, developing a generally applicable model remains challenging, as the issues raised by sampling biases may further compound poor generalizability in psychiatric classification experiments.

Apart from the generalization failures due to sampling bias, there are other pitfalls to cause overfitting as the results of heedless or intended analysis optimization. Overfitting accompanied by accuracy inflation in machine learning models refers that the results are only valid within the data used for optimization but can hardly generalize to other data drawn from the same distribution [29, 30]. In support of this notion, a recent large-scale methodological overview indicated that 87% of machine learning models for clinical prediction exhibited a high risk of bias (ROB) for overfitting, particularly in the domain of psychiatric classification [31]. In addition, variants of methodological parameters that may cause overfitting have been repeatedly discussed in prior review papers: sample-size limitation, in-sample validation, overhyping, data leakage, and especially “double-dipping” cross-validation (CV) methods [32, 33]. The cross-validation procedure is to evaluate the classification performance of the ML model by splitting the whole sample into an independent training set and testing set [32, 34]. Nevertheless, improper CV schemes have been found to overestimate model performance by “double-dipping” dependence or data leakage, which is a main source of incurring overfitting [8]. Besides, a recent review on the application of machine learning for gaining neurobiological and nosological insights in psychiatry underscored the need for cautious interpretation of accuracy in machine learning models [35]. That is, the analytic procedures to obtain reliable model performance are even more critical. However, a comprehensive review that systematically determines these methodological issues in prior studies of psychiatric machine learning classification is currently lacking, and how data/model availability allows for replication analysis to ensure generalization remains unclear. Thus, conducting a meta-research review concerning this topic would facilitate the characterization of the shortcomings and limitations in these current models. Moreover, developing a proof-of-concept assessment tool integrating these issues would facilitate the establishment of a favorable psychiatric machine learning eco-system.

To systematically access the generalizability issues, we conducted a pre-registered meta-research review of current studies that applied neuroimaging-based machine learning models to diagnose psychiatric populations. A total of 476 studies screened from PubMed (n _total_ = 41, 980) over the recent three decades (Jan 1990–July 2021) were included (see Additional file 1: Fig. S1-S2). First, geospatial mapping of the distribution patterns of the samples used in prior literature was depicted to illustrate the sampling biases. Furthermore, capitalizing on the sampling Gini model with the Dagum-Gini algorithm, we quantified the global and area-wide sampling inequality by taking both sampling biases and geospatial patterns into account. The underlying factors of these sampling inequalities were further explored, focusing on economic, social developmental, educational developmental gaps, and psychiatric disorder burdens, with a generalized additive model (GAM). Next*,* we focused on issues of poor generalizability by extending our examination to methodological issues that caused overfitting in previous psychiatric machine learning studies, which facilitated to uncover potential pitfalls that may undermine generalizability. Finally, we utilized the results of our meta-research review to propose a 5-star standardized rating system for assessing psychiatric machine learning quality considering five domains: sample representativeness, cross-validation method, validation scheme for generalization assessment, report transparency, and data/model availability. Associations of study quality scores with publication year, psychiatric category, and model performance were then established.

## Methods

The proposal and protocol for the current study have been pre-registered at Open Science Framework to endorse transparency.

### Search strategy for literature

We searched eligible literature in accordance with PRISMA 2020 statement (Preferred Reporting Items for Systematic reviews and Meta-Analyses, see Additional file 1: Fig. S2). We retrieved literature at the PubMed database, with the following predefined criterion: (1) published from 1990 to 2021 (Jul); (2) peer-reviewed English-written article in journals or in conferences; (3) building machine learning models for diagnosis (classification) towards psychiatric disorders with neuroimaging-based biomarkers. By using Boolean codes and DSM classifications, we retrieved a total of 41,980 records from forty-eight 2^nd^ level psychiatric categories. All records were input into Endnote X9 software for initial inspection and further underwent duplicate removal by using self-made code in Excel suits. Eligible papers were screened strictly following the inclusion and exclusion criteria detailed underneath. Furthermore, to obviate missing eligible records, we hand-inspected the reference list for the newest articles (2021).

We implemented a three-stage validation to ensure the correctness of all the processes. Stage 1: one reviewer was required to perform all the works (e.g., literature searching, data extraction, and data coding) by standard pipeline. Stage 2: a completely independent reviewer was asked to conduct all the works mentioned above for cross-check validation. Stage 3: another independent senior reviewer was designated to check the disparities of results between Stage 1 and Stage 2. If there were incongruences in records, the third reviewers should redo this process independently to determine which one was correct.

### Inclusion and exclusion

We included studies by the following criteria: (1) machine learning models were built to diagnose (classify) psychiatric patients (defined by DSM-5) from healthy control by neuroimaging-based biomarkers; (2) the ground-truth definition for patients was in accordance with clinical diagnoses performed by qualified staffs (e.g., clinical psychiatrists, DSM-5 or ICD-10); (3) fundamental information was given, such as bibliometric information, classifier, model performance, and sample size for both the training set and testing set. More details can be found in Additional file 1: Fig. S1.

We excluded studies that provided no original machine learning models and non-peer-reviewed results, including reviews, abstract reports, meta-analyses, perspectives, comments, and pre-printing papers. Furthermore, studies would be ruled out if they build models by non-machine learning algorithms or reported model performance with non-quantitative metrics. In addition, researches training machine learning models by non-neuroimaging-based features (e.g., genetics and blood markers) or in nonhuman participants were excluded in the current study. As aforementioned, we also discarded eligible studies if the patients’ group had not yet been diagnosed by qualified institutes or medical staff. Finally, studies aimed at non-diagnostic prediction (e.g., prognostic prediction and regressive prediction) were removed for formal analysis.

### Data extraction and coding

To ensure transparency and reproducibility, we extracted and coded data by referring to guidelines, including PRISMA [36], CHARMS checklist [37] (CHecklist for critical Appraisal and data extraction for systematic Reviews of prediction Modeling Studies), and TRIPOD [38] (Transparent Reporting of a multivariable prediction model for Individual Prognosis Or Diagnosis) statement. As mentioned above, the three-stage validation was adopted here to ensure the correctness of these data. We coded eligible studies from two parts, with one for metainformation (e.g., publication year, affiliation, and countries for first author and journals) and another one for the scientific contexts of machine learning models (e.g., sample population, model performance, toolkit, feature selection methods, data availability, and sample size). Full contexts on data extraction and coding can be found in Additional file 1: Fig. S1.

### Data resources

Less (more) economic developed countries (LEDC and MEDC) were defined by using the United Nations Development Programme (UNDP) criteria and International Monetary Fund (IMF, 2020) classification [39, 40]. Following that, a total of 34 countries or regions have been classified as MEDC, such as the USA, Germany, the UK, Japan, and Korea. Data for national development metrics derived from World Bank (WB)-World Development Indicators (2021), including Gross Domestic Product (GDP), Human Development Index (HDI), total government expenditure on public education (GEE), and research and development expenditure (R & D). In addition, we extracted data recording mental health disease burden (MHDB) and prevalence of psychiatric diseases from the Global Burden of Disease Study 2019 (GBD 2019) and the Global Health Data Exchange (GHDx) database. Finally, we obtained metrics for evaluating journal impacts by Journal Citation Reports of Clarivate ™ (2020).

### Geospatial models

We built a global geospatial distribution model by packages of R, including the “ggplot2” and “maptools”. The global geospatial map was defined by 251 countries or regions, which was validated by EasyShu suits. Furthermore, the geospatial maps for the USA, Germany, and the UK have been built by public dataset (CSDN communities). In addition, the geospatial pattern of China was built by the EasyShu software 3.2.2 for interactive visualization. Given the overlapping dataset, the global map visualizing the results of this geospatial model in the present study may be highly similar (but not equal) to the one in our previous work [22].

### Sampling inequality coefficient

To quantify sampling bias and geospatial pattern for sampled population, we estimated sampling inequality based on the Dagum-Gini algorithm [41]. We estimated the Gini coefficient with Dagum-Gini algorithm by fitting multiple Lorenz curves, with absolutely high values for high sampling inequality. Specifically, we defined a relatively total sample size into each grid cell (e.g., each state in a country or each country in the world) based on extracted data in these eligible studies. Furthermore, the sub-modules were set by economic classification (i.e., MEDC and LEDC). Lastly, the Dagum-Gini model was used to decompose contribution from module-between variance, module-between-net variance, and intensity of transvariation. In this vein, we could estimate the Gini coefficient by adjusting the geospatial pattern and relative economic gap for a given economic entity, which improved statistical rigors by controlling unexpected variances. To validate the robustness of the Gini coefficient, we also calculated the Theil index based on the information entropy algorithm.

### Case–control skewness

We calculated case–control skewness to estimate the extent to which the sample size between patients and the healthy control (HC) group was unbalanced, with a high value for high case–control skewness. We estimated the ratio of the number of patients to HC when the sample size in the patient group was larger than the HC group and vice versa, which was used as a metric to quantify the case–control skewness.

### Statistics

**Fig. 1:**
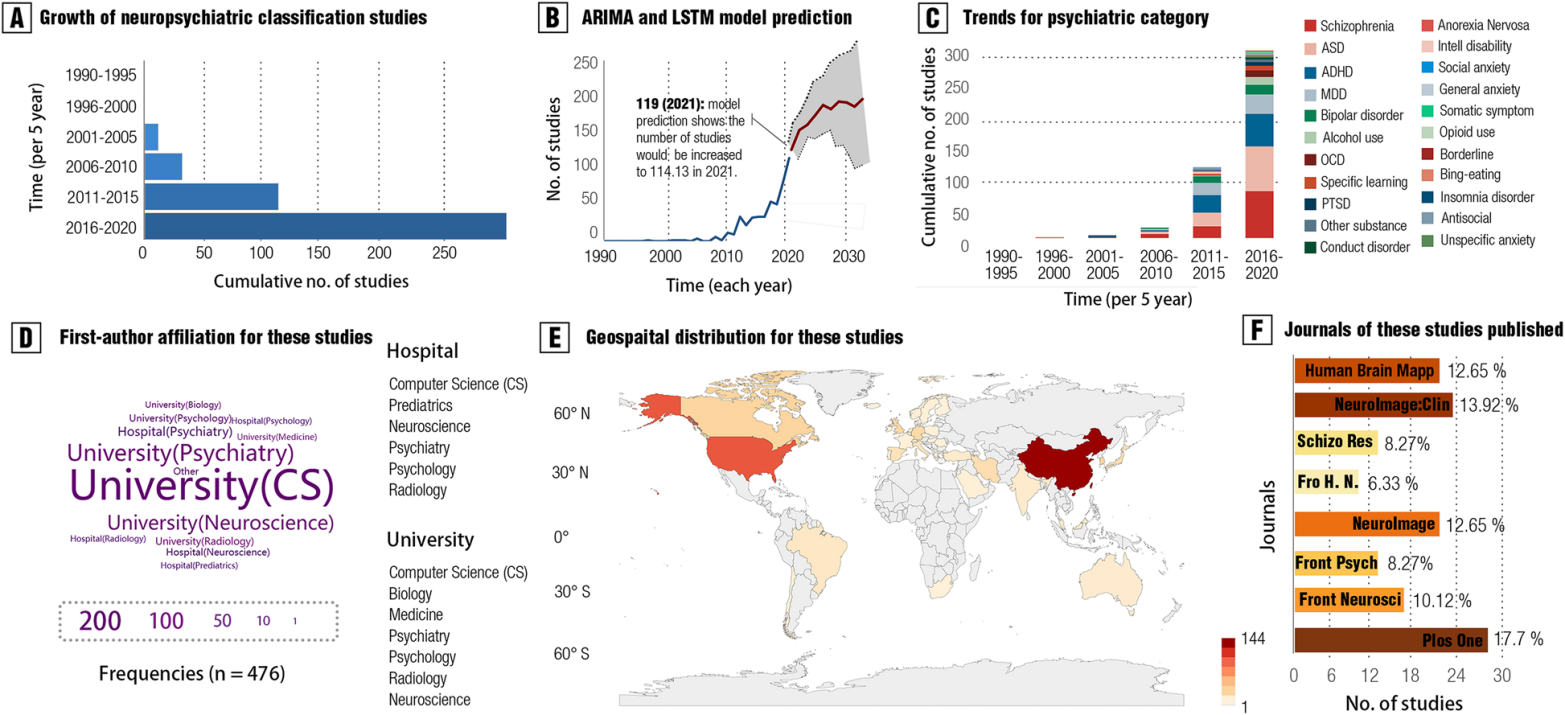
Trends for research aiming at neuropsychiatric diagnostic prediction (classification) during the recent three decades (1990–2020). **A** illustrates the growth of the number of studies concerning neuropsychiatric classification from 1995 to 2020. **B** shows a prediction of the number of relevant studies for future decades based on both the autoregressive integrated moving average (ARIMA) model and the long short-term memory (LSTM) model. The number of relevant studies in 2021 was used as a testing set in the real world. We trained these models with data from 1990 to 2020 and tested them by using real data in 2021 to show the well generalizability. The models predicted the number of relevant studies would be increased to 114.13, and we found that the actual number of these publications in 2021 was 119. **C** presents trends for each psychiatric category during 1990–2021 (June). **D** shows the frequencies of first-author affiliation for all the included studies. **E** mapped the number of countries for the first affiliation in these included studies by using R packages “maptools” and “ggplot2”. **F** illustrates which journals prefer to publish these studies. The top–bottom rank for these journals was determined by the number of these studies adjusted by the total number of publications per year. The length of the bar shows the proportion of one journal including these studies on all the journals

To examine the monotonic increasing trends for time-series data, we capitalized on the non-parametric Mann–Kendall Trend by using the R package [42]. Furthermore, we built both ARIMA (autoregressive integrated moving average) model and LSTM (long short-term memory) model to perform time-series prediction for the incremental trends of the number of relevant studies during the future decade, which were implemented by Deep Learning Toolbox embedded in MATLAB 2020b (MathWorks ® Inc.). Both models were trained by data split from 90% in the whole dataset and were tested in the remaining 10% dataset. Notably, we tested this model with real-world data using the actual number of relevant studies at the end of 2021 (Dec. 30) (see Fig. 1b).

Given the failure in fulfilling the prerequisites of parametric estimation, the Spearman rank model was defaulted for correlation analysis in the current study. Also, the parametric models for validating these correlations have been built as well. Furthermore, the 95% confidence interval (CI) has been estimated by using Bootstrapping process at *n* = 1,000. Equivalent Bayesian analytic models have been constructed as well for providing additional statistical evidence. We used the Jeffreys-Zellner-Siow Bayes factor (BF) with prior Cauchy distribution (*r* = 0.34), with BF > 3 for strong evidence. To examine the non-linear associations of these variables of interest, we have built the generalized additive model (GAM) with natural shape-free spline functions by R package (“mgcv”). To obviate overfitting, the shape-free splines (i.e., smooth function) were used in these models. Finally, metrics of model performance (i.e., classification accuracy) for each study were precision-weighted rather than the original ones as reported.

### Checklist for quantitative assessment on quality

We evaluated study quality in terms of the following five facets that were integrated from these meta-analytic findings: sampling representativeness (item 1: sample size and sites), model performance estimation (item 2: CV scheme), model generalizability (item 3: external validation), reporting transparency (item 4: reports for model performance) and model reproducibility (item 5: data/model availability). By ExpertScape™ rank and peer recommendations, we attempted to reach out to peer experts in multidisciplinary domains to examine the validity of this checklist, including data/computer science, psychiatry, neuroscience, psychology, clinical science, and open science. To this end, we have received concerns or advice for item classification and scoring criteria from three independent experts, and have performed four rounds of revision to form a final 5-star rating system called “Neuroimaging-based Machine Learning Model Assessment Checklist for Psychiatry” (N-ML-MAP-P). Three-stage validation was used to ensure assessment quality as well. Scores for one study would be reevaluated by a third independent scorer once the absolute difference between two scorers was larger than 2 points.

## Results

### General information

Four hundred and seventy-six studies with 118,137 participants from the 41,980 papers were eligible in this meta-research review (see Methods). These studies covered 66.67% (14/21) psychiatric disorders defined by the DSM-5 classification [43]. Diagnostic machine learning classifiers were mostly for schizophrenia (SZ, 24.57%, 117/476), autism spectrum disorder (ASD, 20.79%, 99/476), and attention deficit/hyperactivity disorder (ADHD, 17.85%, 85/476). To probe whether such research interests converged with the healthcare needs, we examined the association between the total number of machine learning studies concerning each psychiatric disorder and their prevalence/disease burdens (Data source: Global Health Data Exchange, GHDx) [44, 45]. Our findings indicated that there was no significant association between the number of studies related to different psychiatric disorders and their real-world prevalence (rho =  − 0.24, *p* = 0.47; BF_01_ = 1.4, moderate evidence strength for supporting the null hypothesis). Furthermore, we found no significant association between the number of these studies for psychiatric disorders and corresponding DALYs (i.e., disability-adjusted life-years)/YLDs (i.e., years lived with disability) that reflected disease burden (DALYs, rho =  − 0.05, p = 0.89; BF_01_ = 2.5; YLDs, rho =  − 0.06, *p* = 0.89; BF_01_ = 2.6, moderate evidence strength for null association). Based on World Bank (WB) and International Monetary Fund (IMF 2021) classification, populations sampled in these studies were from 39 upper-middle-income to high-income countries, leaving population from the remaining 84.46% (212/251) countries in the globe unenrolled. In addition, 59.45% (283/476) of these studies used domestically-collected samples, while 31.10% (148/476) reused open-access datasets (e.g., ABIDE and ADHD-200).

### Historical trends

The total number of psychiatric machine learning studies for diagnostic classification on psychiatric disorders increased markedly in the past 30 years (*z* = 5.81, *p* = 6.41 × 10 ^−9^, Cohen *d* = 1.82, Mann-Kendell test) (see Fig. 1a and Additional file 1: Fig. S3). Based on time-series prediction models, we predicted a persistent increment for the number of studies pursuing brain imaging-based diagnostic classification for psychiatric disorders in the future decade (e.g., *k* = 229.65 in 2030, 95% CI: 106.85–352.44) (see Fig. 1b and the “ Methods” section).

Despite the accelerated increase in the number of psychiatric machine learning studies, the increase rate for different psychiatric disorders was found to be different: the number of existing studies on SZ, ASD, ADHD, major depression disorder (MDD), and bipolar disorder (BP) is significantly larger than that on other high-disease-burden categories (e.g., eating disorder and intellectual disability) (see Fig. 1c). To quantify the increment pattern for different psychiatric categories, we capitalized on increment curve models. We found that increase speeds for machine learning models regarding neuropsychiatric diagnoses towards SZ (*b* = 2.40, 95% CI: 2.05–2.74, *p* < 0.01) and ASD (*b* = 2.64, 95% CI: 2.25–3.02, *p* < 0.01) were significantly faster than others (see Additional file 1: Fig. S3 and Tab. S1).

Interestingly, a quite number of first authors of these studies (46.42%, 221/476) seemed to be trained in computer and data science instead of psychiatry or neuroscience (see Fig. 1d). Institutes from China, the USA, Canada, Korea, and the UK contributed mostly for the total number of these machine learning studies (see Fig. 1e). Moreover, by adjusting the total publications per year, we found that these studies were mostly published in journals with a special scope on neuroimaging, such as *Human Brain Mapping* and *Neuroimaging: Clinical* (see Fig. 1f and Additional file 1: Tab. S2-S3).

### Sampling bias and sampling inequality

#### Geospatial pattern of sampling bias

**Fig. 2:**
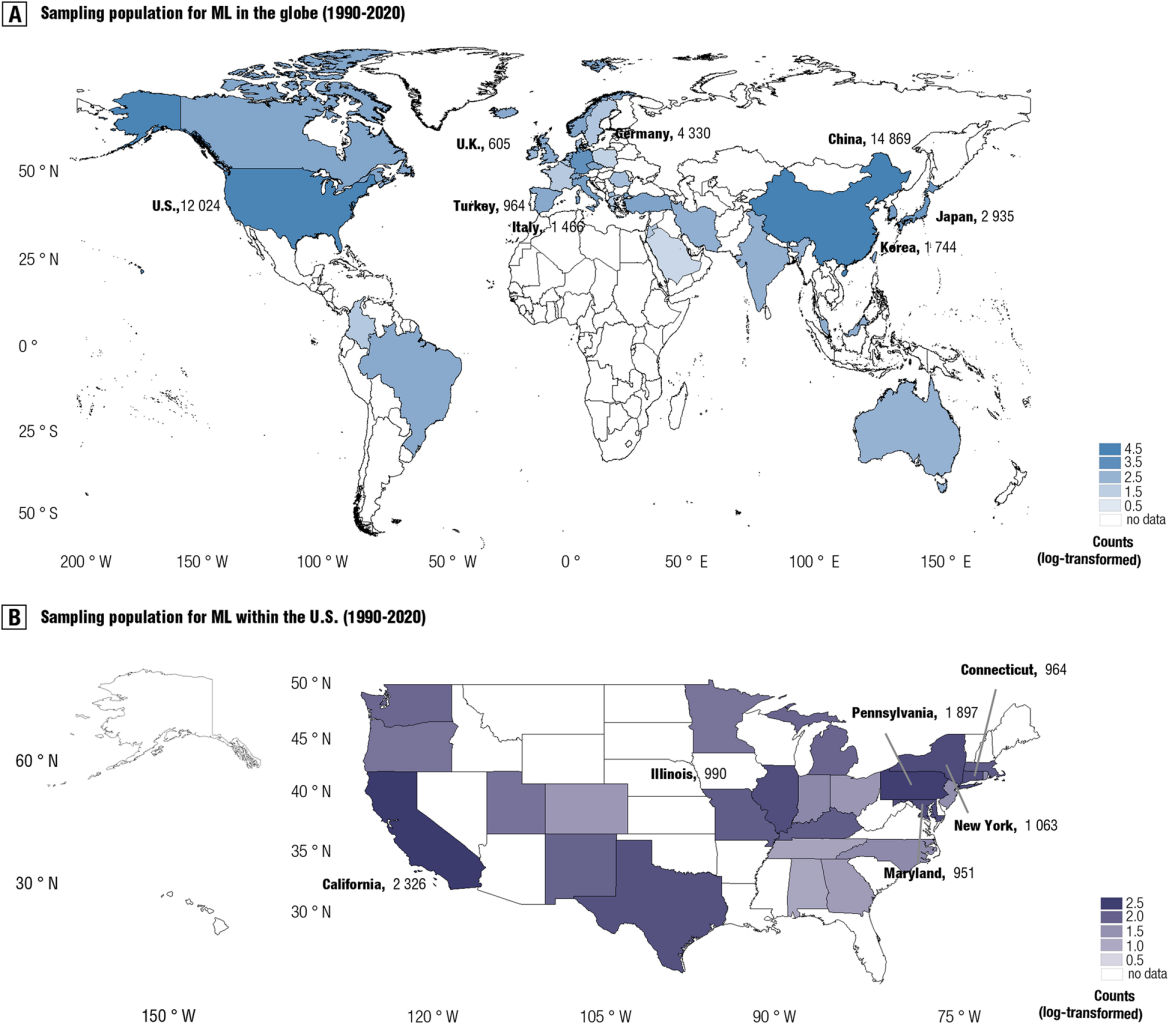
Geospatial model for sample population regarding ML models towards neuropsychiatric classification in the world (**A**) and USA (**B**). Both maps were built by 1st administrative grid cell, with each country/region for the globe (251 countries/regions) and state for the USA (51 states). For better readability, we re-scaled the sample size by log-transformation. Sample size for a portion of countries/regions has been shown in these maps. **A** panel was depicted similarly to the Fig. 2A in our previous article [22], because of the overlapping datasets between them

Geospatial maps were generated to visualize the distribution of the sampled populations (i.e., the number of participants). We found that the sample populations covered only the minority upper-middle-income and high-income countries (UHIC) worldwide (*n*_UHIC countries_ = 32; 12.74%). Even in UHIC, across-country imbalance in sample population was striking (total sample size: *n*_Chinese_ = 14,869, *n*_Americans_ = 12,024, *n*_Germans_ = 4, 330; see Fig. 2a and Additional file 1: Tab. S4). Moreover, we found a likewise prominent within-country imbalance of sample populations (see Fig. 2b, Additional file 1: Tab. S5-S8 and Additional file 1: Fig. S4-S5). Furthermore, as for continents-based classification, populations of these machine learning models were largely enrolled from Asia (44.67%, adjusted by total population) and North America (26.76%, adjusted by total population). Notably, in the current meta-research review, no machine learning models were observed to train classifiers by samples in Africa despite its large population.

**Fig. 3:**
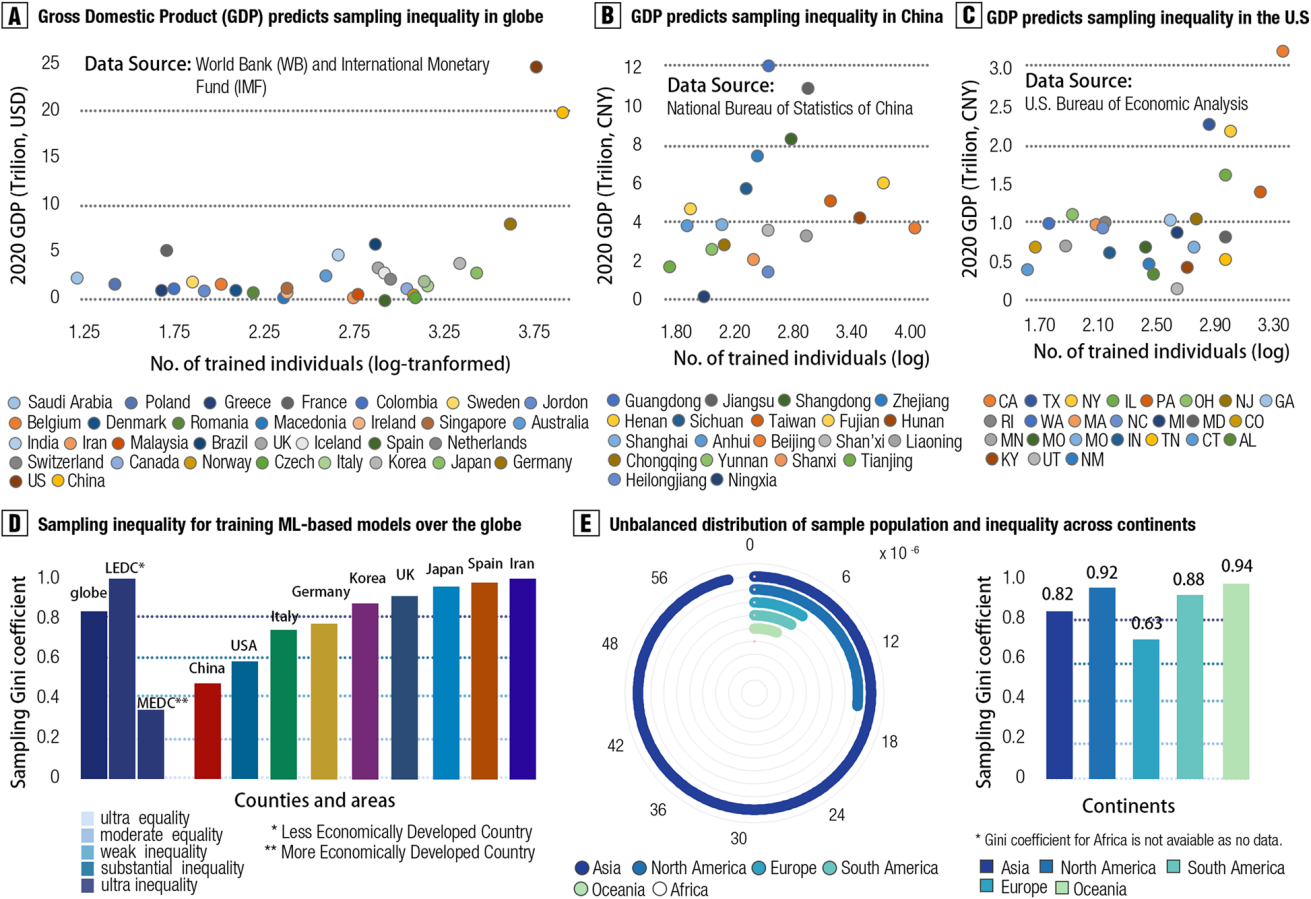
Sampling bias and sampling inequalities in these trained ML models. **A** provides a scatter plot for the association between GDP and sample size for 32 counties/regions in the globe. **B** offers a scatter plot showing the association between GDP and sample size for 20 provinces within China. (C) shows the association between GDP and sample size for 25 states within the USA. (D) plots Gini sampling coefficients for the top 10% countries with large sample sizes to train ML models in existing studies, with high Gini value for high sampling inequality. LEDC and MEDC were categorized by World Bank (WB) and International Monetary Fund (IMF) classification. **E** illustrates the sampling bias and Gini coefficients for each continent. The left panel shows the proportion of the total sample size for training ML models in existing studies on the total sample population for each continent. The right panel shows the Gini coefficient for each continent

We examined whether the size of the sample population in these models could be determined by the national economic level. Results showed a strikingly positive association between nation-wide GDP (Gross Domestic Product, Data source: IMF 2021) and total sample size all over the globe (*r* = 0.65, 95% CI: 0.40–0.81, conditions-adjusted, *p* < 0.001; BF_10_ = 1.10 × 10^3^, Strong evidence) (see Fig. 3a). Supporting that, such association was found within China (r = 0.47, 95% CI: 0.02–0.76, *p* < 0.05, conditions-adjusted; BF_10_ = 1.93, moderate evidence) and the USA (*r* = 0.47, 95% CI: 0.10–0.73, *p* < 0.05, conditions-adjusted; BF_10_ = 3.72, Strong evidence), respectively (see Fig. 3b–c).

#### Sampling inequality

To quantitatively evaluate such sampling bias, the new concept, sampling inequality, was introduced, which reflects both the sample-size gap and the geospatial bias for the sampled populations reported in existing psychiatric machine learning studies. We used sampling Gini coefficient (*G*, ranged from 0 to 1.0) based on the Dagum-Gini algorithm, to quantify the degree of sampling bias (see Methods). We found severe sampling inequality in samples of prior psychiatric machine learning studies (*G* = 0.81, *p* < 0.01, permutation test, see Fig. 3d). Furthermore, based on IMF classification, we grouped global countries into More Economically Developed Country (MEDC) bloc and Less Economically Developed Country (LEDC) bloc and found a significant difference in the sampling inequality between them: sampling Gini coefficient in LEDC was threefold (*G*_LEDC_ = 0.94, *G*_MEDC_ = 0.33, *p* < 0.01, permutation test) higher than that in MEDC. In addition, we also examined within-country sampling inequality. Results showed a weak sampling inequality in China (*G* = 0.47) and the USA (*G* = 0.58), but severe inequality in Germany (G = 0.78), the UK (*G* = 0.87), Spain (*G* = 0.91), and Iran (*G* = 0.92) (see Fig. 3d and Additional file 1: Tab. S9). Furthermore, we found a relatively lower sampling inequality in Europe compared with other continents (*G*_Europe_ = 0.63; see Fig. 3e and Additional file 1: Tab. S10-S11). Notably, a significantly positive association between these sampling Gini coefficients and averaged classification accuracy was uncovered (*r* = 0.60, *p* = 0.04, one-tailed; permutation test at *n* = 10,000), which possibly implied potential inflated estimates for model performance because of such sampling inequality.

To examine whether sampling inequality was further increased by economic gap, that was, individuals (patients) living in richer countries (areas) were more likely to be recruited in building rich-areas-machine learning-specific models, a generalized additive model (GAM) with natural shape-free spline function was constructed. Interestingly, the GDP of these countries allows for an accurate prediction of the sampling inequality values (*β* =  − 2.75, S.E = 0.85, *t* = 4.75, *p* < 0.001, *R* ^2^_adj_ = 0.40; *r* =  − 0.84, 95% CI: − 0.41 to − 0.97, *p* < 0.01; BF_10_ = 13.57, strong posterior evidence), with higher national income for weaker sampling inequality. The apparent presence of sampling bias and high sampling economic inequality for the reviewed psychiatric machine learning studies may resonate with generalization failure that was widely concerned in the field.

### Methodological considerations on generalizability

#### Sample size, validation, technical shifting, and case–control skewness

**Fig. 4:**
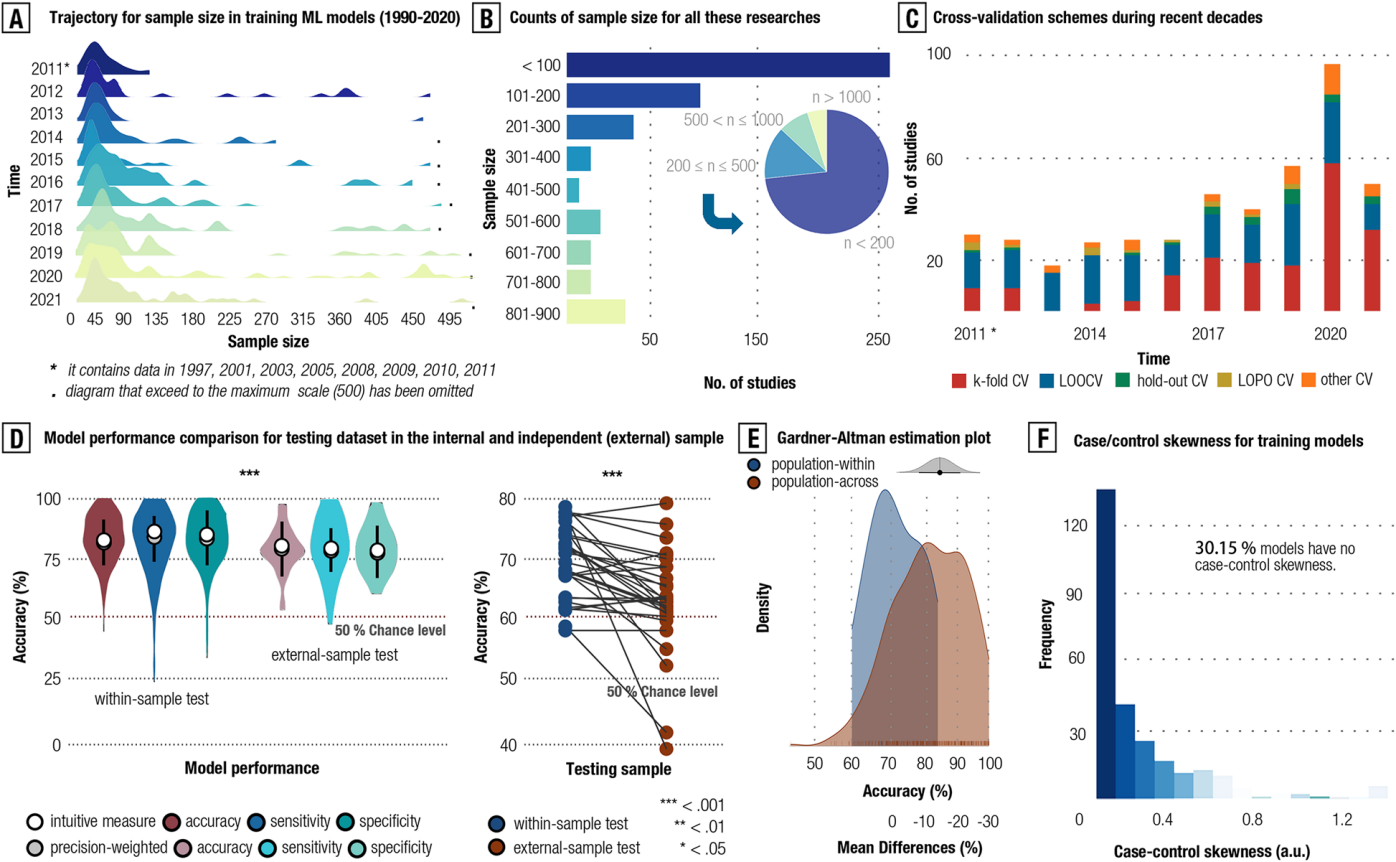
Methodological considerations for existing ML models towards psychiatric diagnosis. **A** illustrates increment trends for sample size during the recent three decades by Gaussian kernel density plots. Labeling 2011 sums up all the sample size from 1990 to 2011. **B** shows the counts for subgroups by dividing these studies according to sample size. **C** plots the trends of using cross-validation (CV) schemes by accounting counts from all the included studies during the recent three decades. **D** shows model performance comparisons between independent-sample validation and within-sample validation. The non-parametric W test was used for statistical inferences, with *** for *p* < .00. Precision-weighted accuracy was estimated by Woo et al. **E** depicts Gardner-Altman estimation for the classification accuracy comparison between population-within sample and population-across sample. Black dot indicated the point estimate for the mean difference (delta) of the two groups, and the shadow areas showed the distribution estimated by delta. **F** presents a frequency plot to show case–control skewness

We extended the investigations of sampling bias and sampling inequality to an analysis of other methodological facets that may likely lead to overfitting and hence magnify the generalization errors. A significant correlation was found between the sample size in psychiatric machine learning studies and publication year in the last three decades (*r*_(total)_ = 0.75, 95% CI: 0.22–0.93, *p* = 0.013; BF_10_ = 5.83, Strong evidence) (see Fig. 4a and Additional file 1: Tab. S12-S14). Despite improvement over time, we observed a strikingly biased distribution skewing to a small sample size (*n* < 200) in these machine learning models (73.10%, 348/476) (see Fig. 4b and Additional file 1: Tab. S15).

In addition, we found a prominently positive association between the ratio of using k-fold cross-validation (CV) scheme and publication year in recent decades (*r* = 0.82, 95% CI: 0.40–0.95 *p* < 0.01; BF_10_ = 15.80, Strong evidence) (see Additional file 1: Tab. S16-S17). As repeated recommendations by didactic technical papers [8, 10, 34, 46], adopting a k-fold CV to validate model performance could outperform popular LOOCV methods in terms of model variance and biases. We thus examined model performance between them by precision-weighted method [47] that could adjust the effects of sample size and between-study heterogeneity. Results showed that model performance estimated by LOOCV was prominently higher than k-fold CV (Acc _LOOCV_ = 80.35%, Acc _k-fold_ = 76.66%, precision-adjusted, *w* = 20,752, *p* < 0.001, Cohen *d* = 0.31; BF_10_ = 2289, strong evidence) (see Fig. 4c). Details for other methodological considerations can be found at Additional file 1: Tab. S18-22.

As for independent-sample validation, we found a significantly positive association between the ratio of validating model performance in the independent sample (site) and publication year in recent decades (*r* = 0.88, 95% CI: 0.63–0.97 *p* < 0.01; BF_10_ = 234.93, Strong evidence). Nevertheless, the majority of these machine learning studies (84.24%, 401/476) still lacked validation for model generalizability in the independent sample(s). Furthermore, we found that the classification performance of these models tested in the independent samples was more “conservative” than those tested in the internal samples (Acc _independent-sample validation_ = 72.71%, Acc _others_ = 77.75%, precision-adjusted, *w* = 3,041, *p* < 0.001, Cohen *d* = 0.32; BF_10_ = 29.43, Strong evidence) (see Fig. 4d and Additional file 1: Tab. S23). To directly test the impact of sampling bias on model generalizability, we compared the model performance between cross-country samples (i.e., training model in a sample from one country and testing model in a sample from other countries) and within-country (i.e., training and testing model in sample within the same countries) sample. Results showed that model performance was more “conservative” in the cross-country sample than in the within-country sample (Acc _cross-country sample_ = 72.83%, Acc _within-control sample_ = 82.69%, precision-adjusted, *w* = 2,008, *p* < 0.001, Cohen *d* = 0.54; BF_10_ = 150.90, Strong evidence; see Fig. 4e).

Furthermore, we specifically examined the shift of mainstream neuroimaging modalities and features of these models in recent decades. Results showed that the (functional) MRI was still the mainstream neuroimaging technique to build these models over the last three decades (i.e., averaged 73.70% of these models for (functional) MRI, 21.57% for EEG/ERP, 2.69% for fNIRs, 2.02% for MEG and 0.21% for PET). Despite that, the increasing trend of using multi-modalities in training these neuroimaging-based ML models was observed, from 3.22% to 19.19% of these models over time. In addition, with the developments of ML techniques, the ratio of using deep learning models or complicated parameterized models to “shallow learning models” was increasing during recent decades, particularly after 2019 (i.e., 0% in 2012, 10.40% in 2016, and 32.32% in 2020). As for the strategy of feature selection, we found an increase in the applications of algorithmic techniques than of pre-engineered selections in building these models (i.e., 0% before 2012, and averaged 30.90% after 2012). Nevertheless, no changes were found for the shift of paradigm from a single-snapshot case–control cohort to repetitive scanning of the same participants in these models. While the shifting of main neuroimaging modalities, model complexity, and feature selection strategy was observed over time, we found no prominent trends of model performance (i.e., precision-weighted accuracy) over time (Accuracy: 84.43%, 95% CI: 81.84–87.88% at 2011; 84.38%, 95% CI: 80.79–87.86% at 2015; 84.78%, 95% CI: 82.82–87.49% at 2020). Full results for these findings can be found in Additional file 1: Fig. S6-S8.

Finally, by calculating the standardized case–control ratio (see the “Methods” section), we observed a case–control skewness (i.e., the number of patients is larger than healthy control, and vice versa) in a quarter of all the included studies (25.37%, 121/476) (see Fig. 4f). The case–control skewness was significantly (but weakly) associated with the reported classification accuracy, which may imply inflated accuracy due to the imbalanced case–control distribution in the data (*r* = 0.15, 95% CI: 0.04–0.27, *p* < 0.05; BF_10_ = 2.04, moderate evidence).

#### Technical transparency and reproducibility

**Fig. 5:**
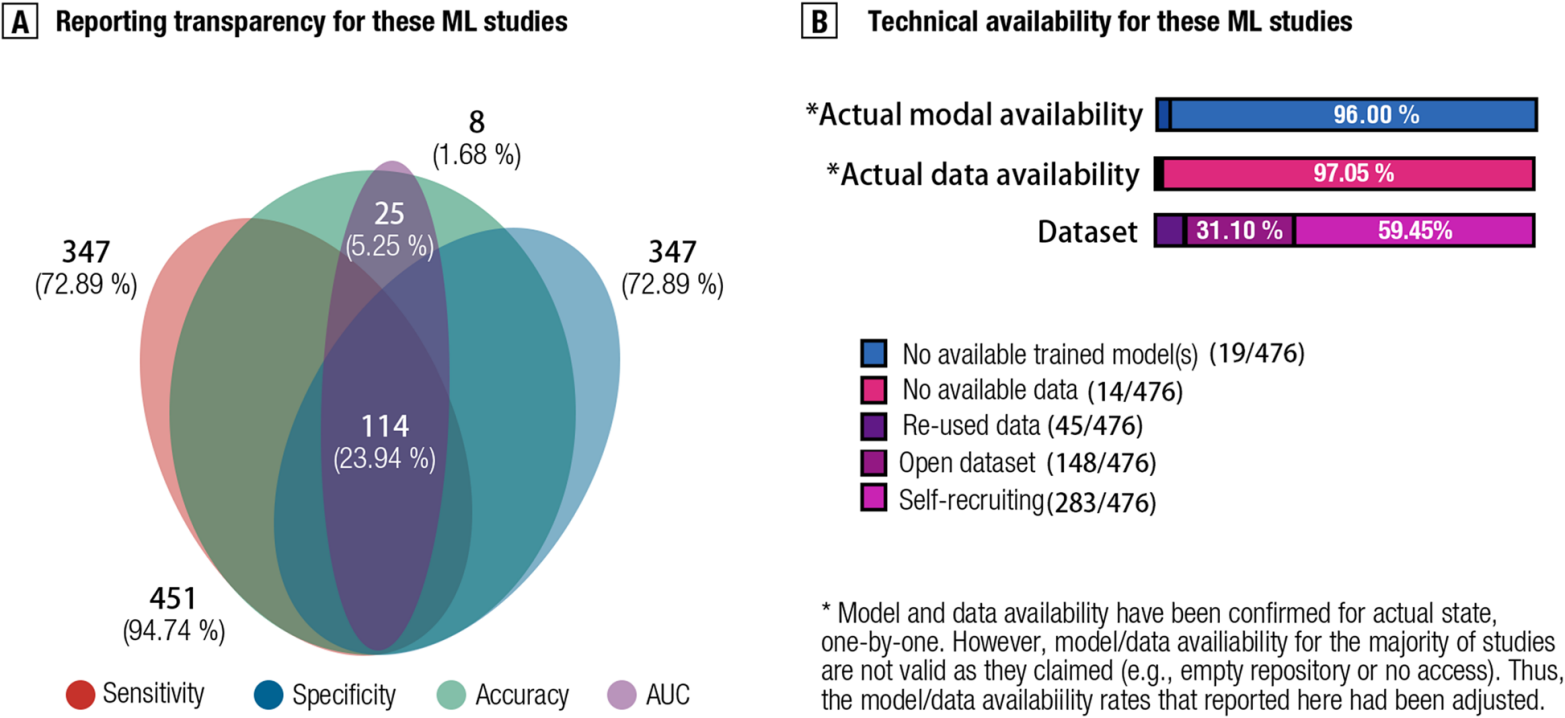
Reporting transparency and technical (data and model) availability. **A** presents patterns of reporting model performance across sensitivity, specificity, balanced accuracy, and area under curve (AUC) by a Venn plot. **B** sums up the proportion of having actual model availability, data availability, and datasets

We further determined whether existing studies provided sufficiently transparent reports to evaluate potential overfitting and reproducibility. We found that only one fifth of them (23.94%, 114/476) fulfilled the minimum requirements for reporting model results (i.e. balanced accuracy, sensitivity, specificity, and area under curve) by the criterion as proposed by Poldrack [8, 48] (see Fig. 5a).

As for the model reproducibility, only 12.25% (58/476) of studies shared trained classifiers (full-length codes). Furthermore, only 19.12% (91/476) studies claimed to provide available original data. Notably, we manually checked the validity of these resources as these studies stated, one-by-one, but found that only a small portion of trained classifiers (32.27%, 19/58) or data (15.38%, 14/91) were actually available/accessible (see Fig. 5b). Thus, incomplete reports for model results and poor technical reproducibility may be one of the sources to hamper the assessment of generalizability, and hence, the “generalization crisis” remains.

### Five-star quality rating system

**Fig. 6:**
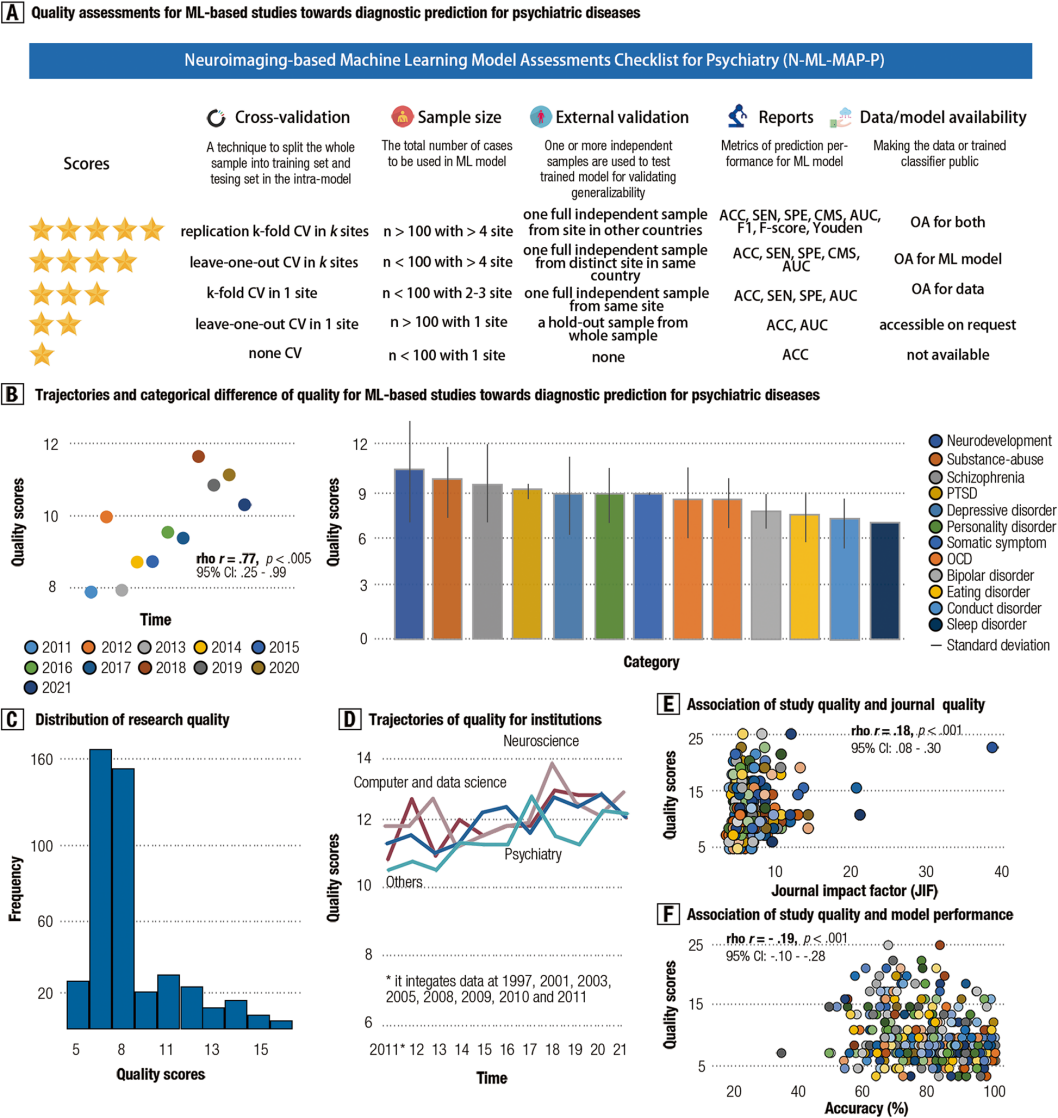
Neuroimaging-based machine learning model assessment checklist for psychiatry (N-ML-MAP-P). **A** provides details for five items and scoring criteria in this checklist for evaluating the study quality of all the included studies. **B** presents a scatter plot for showing the trends of improving study quality during the recent decade (2011–2021). **C** shows the overall study quality for each psychiatric category in existing studies. This plot is ranked by total quality score, and bars indicate standard error (S.E.). **C** provides a frequency plot for overall quality scores. **D** shows the trajectories of study quality for different affiliations, including data/computer science, neuroscience, psychiatry, and others. **E** draws a scatter plot showing the association between journal quality (i.e., journal impact factor, JIC) and overall quality scores. **D** provides a scatter plot to show the association of overall quality scores with model accuracy as reported in these studies

To promote the establishment of an unbiased, fair, and generalizable diagnostic model, we proposed a 5-star quality rating system called “Neuroimaging-based Machine Learning Model Assessments Checklist for Psychiatry (N-ML-MAP-P)” by integrating these meta-research findings aforementioned and up-to-date guidelines that provided by multidisciplinary experts (see Methods). This rating system incorporated five elements, including sample representativeness, CV methods, independent-sample validations, reports for model performance, and data/model availability (see Fig. 6a).

Based on this N-ML-MAP-P rating system, we found that overall quality scores for these models have increased consistently over the last decade (*r* = 0.77, 95% CI: 0.25–0.99, *p* < 0.01; BF_10_ = 7.04, strong evidence), demonstrating that study quality for machine learning models on psychiatric diagnosis has been increasingly improved (see Fig. 6b). In addition, we also examined study quality for each item and revealed that ratings for sample size, CV methods, independent validation, and reporting transparency have been gradually improved (see Additional file 1: Tab. S24). However, we found no prominent increase in quality scores on technical (data and model) availability (see Additional file 1: Tab. S25). Furthermore, we found a considerably strong positive correlation between the number of disorder-specific studies and their quality scores (*r* = 0.69, 95% CI: 0.13–0.88, *p* < 0.05; BF_10_ = 4.10, strong evidence), with relatively high quality for machine learning studies concerning SZ, ASD, and ADHD.

Despite the increase, the overall quality scores remained relatively low in the vast majority of these models (see Fig. 6c–d). Intriguingly, we found a weak but statistically significant association between journal impact factors/journal citation indicator (JIF/JCI) and the scores of model quality rated by N-ML-MAP-P assessment (*r*_(JIF)_ = 0.18, 95% CI: 0.08–0.30,* p* < 0.001; BF_10_ = 41.90, strong evidence; *r*_(JCI)_ = 0.15, 95% CI: 0.06–0.25, *p* < 0.01; BF_10_ = 8.60, strong evidence) (see Fig. 6e). Furthermore, we also observed a weakly negative association between the JIF/JCI and model performance (*r* =  − 0.19, 95% CI: − 0.10 to − 0.28, *p* < 0.001; BF_10_ = 4697.67, strong evidence) (see Fig. 6f).

In summary, our purpose-built N-ML-MAP-P system for quantitatively assessing the quality of these models revealed prominent improvements for them over time, possibly indicating that efforts made by scientific communities [8, 10, 49] to address overfitting issues in diagnostic machine learning models for psychiatric conditions may be effective. However, existing machine learning studies may still face several challenges, e.g., low overall quality and poor technical reproducibility, which still characterize a majority of these studies. A full list of these models can be found in Additional file 2 [50–510].

## Discussion

We conducted a pre-registered meta-research review and quantitative appraisal to clarify generalizability and even quality in existing machine learning models on neuroimaging-based psychiatric diagnosis (*k* = 476) from insights into sampling issues, methodological flaws, and technical availability/transparency. By doing so, we quantified a severe sampling economic inequality in existing machine learning models. By further determining methodological issues, we found that sample-size limitation, improper CV methods, lack of independent-sample validation, and case–control skewness still contributed to an inflation of model performance. Furthermore, we found a poor technical availability/transparency which may in turn critically hamper mechanisms to examine generalizability for these models. Based on these findings, we developed a checklist to quantitatively assess the quality of existing machine learning models. We found that despite increasing improvement, the overall quality of the vast majority of these machine learning models was still low (88.68% models were rated at low quality in existing literature). Taken together, the results indicated that ameliorating sampling inequality and improving the model quality may facilitate to build of unbiased and generalizable classifiers in future clinical practices.

One critical finding that warrants further discussion is the severe global sampling inequality in existing machine learning models. Despite rapid proliferation, we found that the samples were predominately recruited from upper-middle-income and high-income countries (444/476 models, 93.28%). Also, we observed regional sampling inequalities, with the Gini coefficient in LEDC being 3-fold higher than that in MEDC. To make matters worse, both sampling inequalities were found to be enlarged by regional economic gaps. Despite supporting the descriptive observation to previous studies [22, 35, 511], the present study provided unique statistical evidence to clearly reveal the severity of sampling bias of these extant models in the globe or across countries (regions), which advanced our knowledge to make the sampling bias quantitatively comparable rather previously conceptional concern. Beyond that, the predictive role of national (regional) economic level on these sampling biases has been quantitatively verified, possibly indicating a sampling economic inequality in the neuroimaging-based ML-aid diagnosis. For instance, in China, machine learning models were predominately trained by samples from mid-eastern Chinese with high incomes, whereas there was no evidence to validate whether these trained machine learning models could be generalized for western Chinese with lower incomes. Notably, it is the same case with predictive models, recent works have revealed generalization failure for cross-ethnicity/race samples in neuroimaging-based predictive models [512, 513]. Compared to previous studies using qualitative inferences or conclusions [514, 515], we provided preliminary evidence to quantify the extent to which sampling inequality impacted model generalizability, this result may imply how much sampling inequality should be limited to built generalizable neuroimaging-based diagnostic classifiers. As a didactic example recently, Marek and colleagues (2022) have provided a quantitatively empirical evidence that thousands of subjects are needed to attain reliable brain-behavior associations, although the intensive discussions or concerns for the high overfitting in neuroimaging-based models with small sample sizes have been debated for a long-lasting time [10, 34, 46, 516]. Thus, by quantitatively revealing the association of sampling issues to inflated classification accuracy, the present study may provide valuable insights into how to increase sampling equality enough to achieve good model generalizability in future empirical studies. To tackle this issue, diversifying sample representation (i.e., racial balance or socioeconomic balance) in neuroimaging-based predictive models has been increasingly advocated [517, 518]. That is, existing “population-specific models” trained with less or even no samples in LEDC and Africa practically questioned their generalizability across intersectional populations. More importantly, besides common sense for the disadvantages of global economic gaps in science development, “leaving the poor ones out” in training machine learning models may not only render poor generalizability to psychiatric diagnostic models but also exacerbate global inequalities in clinical healthcare. Relating to this consideration, future studies could explore whether and how economic gaps contribute to biases in clinically diagnostic measures, such as neuroimaging-based precision diagnosis in high-income countries, as opposed to more subjective symptom-dependent diagnosis in low-income countries. This investigation could further our understanding of how economic disparities impact the inequalities in the development and implementation of diagnostic basis. Nonetheless, another insightful viewpoint worthy to note was that an overly board emphasis on generalizability may impede clinical applications of these machine learning models in specific medical systems (e.g., healthcare) [519, 520], with high generalizability at the expense of optimal model performance within specific cohorts. In other words, despite poor geospatial or socioeconomic generalizability, these machine learning models posing high performance within specific contexts (e.g., machine learning model trained by data in the A hospital could accurately predict patients within A hospital rather than other ones) may be still reliable into a given clinical practice.

Another factor contributing to poor generalizability was rooted in methodological issues. We found that, with consistent efforts made by scientific communities [8, 521], the ratio of using k-fold CV in estimating model performance gradually increased during recent decades, which may partly mirror effective controls for overestimation on diagnostic accuracy that was caused by flawed CV scheme [23, 34]. However, the LOOCV was still used widely (40.33%) in recent decades, which may overfit models compared to those using k-fold CV (precision-weighted classification Acc _level-one-subject-one CV_, 80.35%; Acc _k-fold CV_, 76.66%, *p* < 0.001). Thus, although the repeated technical recommendations and calls may be effective in changing our practices to rectify model overfitting, this issue has not been fully addressed to date [522, 523]. Compared to the CV method, testing model performance in external (independent) samples could provide more accurate estimates for interpretability and generalizability [524, 525]. Nevertheless, only 15.76% models were validated in the independent sample (s). More importantly, we observed that model performance may be highly overestimated in within-country independent samples compared to cross-country ones (precision-weighted classification Acc _cross-country_, 72.83%; Acc _within-country_, 82.69%, *p* < 0.001). Thus, not only “independent-sample” validation but also the well-established “intersectional-population-cross” validation is demanded to strengthen generalizability in future studies [526]. Moreover, few machine learning models (< 5%) provided adequate technical availability, which diluted our confidence for the generalizability and reproducibility of these models, especially in the “big data” era [527]. On balance, we found that methodological flaws of these machine learning models were increasingly ameliorated to prompt model generalizability in recent decades, but sample limitation, improper CV methods, lack of “cross-population” independent-sample validation, and poor technical availability still exposed these models to high risks of overfitting.

In the current study, we proposed a quantitative framework for evaluating the quality of these models, covering sample, CV, independent-sample validation, transparency, and technical availability. We found that the overall quality of these models increasingly improved over time. As aforementioned, some didactic methodological papers [8, 9, 23] have considerably contributed to prompting the scientific communities to rectify these methodological flaws in machine learning models. Furthermore, reporting benchmarks or guidelines were also developed to increase the transparency of information for accurately evaluating model performance in recent years [38, 528]. However, despite encouraging improvements, the low-quality machine learning models seem to still dominate this field, as we observed in the current study that single-site samples and poor data/model availability remained largely unchanged [15]. Together, the findings may imply that existing machine learning models are not as solid as claimed in terms of generalizability and reproducibility in clinical practices in their current form. It is noteworthy that the high-quality models that were rated in the current study have not yet been tested for generalizability. Thus, testing the generalizability or reproducibility of these models from originally trained samples to different populations (e.g., countries, ethnics, income-levels) could be a more reliable and valid way to validate the generalizability in future studies.

To tackle these generalizability issues, here we recommended several practical tips. Beyond sample size, recruiting a diverse, economically-equal, case–control balanced, and representative sample is one of the best avenues to obviate sampling biases. Technically speaking, the cross-ethnicity/race or cross-country independent sample should be prepared for the generalizability test. At least, the nest k-fold CV method is clearly warranted. Moreover, we also recommend transparent and unfolded reports for model performance facilitating to take these models into clinical insights. In addition, improving the data/modal availability for these models is one of the ways to provide venues to validate clinical applicability. To conceptualize and streamline these recommendations, we have preliminary built the “Reporting guideline for neuroimaging-based machine learning studies for psychiatry” (RNIMP 2020) checklist and diagram (see Additional file 3: Tab. S1-2); this suit is developed by encapsulating the above tips and currently promising benchmarks [8, 23].

This study warrants several limitations. First, we narrowed the research scope into diagnosis but not all the categories were sampled evenly. Predictive machine learning models for psychiatric conditions included (at least) three forms of prediction: diagnosis (i.e., predicting the current psychiatric condition), prognosis (i.e., predicting outcome in future onsets), and prediction (i.e., predicting response or outcome for a given treatment) [529]. Second, this study may not cover all eligible data, especially in literature that was published in African areas or written in non-English languages, as data were screened from English-written peer-reviewed papers. Therefore, we stress that all the findings are grounded on these studies, instead of completely representing the real-world situation. Third, the current study did not probe into the model generalizability from biological insights, but focused on sampling bias and methodological issues only. Thus, it left room to be uncovered in future works. Fourth, we empirically inferred the academic training experiences of the first authors by their affiliation. However, such assumptions may not be solid. Extending these conclusions from this section to elsewhere should be more prudent. Fifthly, the present study has not thoroughly analyzed the factors that contribute to the changes in the methodological and technical underpinnings of machine learning models for psychiatric diagnosis. For instance, the increased sample size in these models over the last decades may be attributed to the improvements of imaging techniques/infrastructures, the developments of machine learning knowledge, and the decrease of data costs, which are not explored in the current study. In other words, future studies could reap huge fruits from delving into the specific roles of these factors in the advances of these models, particularly in sample size. Lastly, given that the neuroimaging-based signatures are not practically applicable for diagnosing all psychiatric conditions, the statistics for unbalanced developments and qualities across these DSM categories should be explained more prudently.

## Conclusions

On balance, we provided meta-research evidence to quantitatively verify the sampling economic inequality in existing machine learning models for psychiatric diagnosis. Such biases may incur poor generalizability that impedes their clinical translations. Furthermore, we found that the methodological flaws have been increasingly ameliorated because of repeated efforts made by these technical papers and recommendations. Nonetheless, in the present study, we stretched views to find that these limitations including small sample size, flawed CV method (i.e., LOOCV), no independent-sample validation, case–control skewness, and poor technical availability still remained, and have demonstrated quantitative associations of such limitations to inflated model performance, which may hence indicate model overfitting. In addition, poor reporting transparency and technical availability were also observed as a hurdle to translate these models into real-world clinical actions. Finally, we extended to develop a 5-star rating system to provide a purpose-built and quantitative quality assessment of existing machine learning models and found that the overall quality of a vast majority of them may still be low. In conclusion, while these models showed a promising direction and well-established contributions in this field, it is suggested that enhancing sampling equality, methodological rigor, and technical availability/reproducibility may be helpful to build an unbiased, fair, and generalizable classifier in neuroimaging-based machine learning-aid diagnostics of psychiatric conditions.

## Supplementary Information

**Additional file 1: ** Figure S1-S8 and Table S1-S25. **FigS1.** Research pipelines for data acquisition. **FigS2.** PRISMA 2020 flow diagram for the current study. **FigS3.** Trends in ML-based diagnostic prediction for psychiatric diseases by neural features. **FigS4.** Mental health disorders as the portion of total disease burden at 2019. **FigS5.** Geospatial model for sampling population within China, Germanyand U.K. **FigS6.** Distribution of methodological details. **FigS7.** Model performance across algorithm, tookit, cross-validation, sample sizeand skewness. **FigS8.** Model performance across validations, trajectories, psychiatric categories, journal impacts, scanning technology/modalityand institutes/datasets. **TabS1.** Curve fitting results for exponential function model. **TabS2.** Journals counts for papers aiming at neuropsychiatric diagnostic prediction. **TabS3.** Counts for contributors’ sources for these papers. **TabS4.** Summary for sample population for these papers in the world. **TabS5.** Summary for sample population for these papers in the U.S. **TabS6.** Summary for sample population for these papers in the China. **TabS7.** Summary for sample population for these papers in the Germany. **TabS8.** Summary for sample population for these papers in the U.K. **TabS9.** Sampling inequalities for globe and countries/regions. **TabS10.** Sampling inequalities for continents. **TabS11.** Sampling inequalities and national development index. **TabS12.** Sample size during recent decade for all the studies. **TabS13.** Sample size during recent decade for studies using self-recruiting sample. **TabS14.** Sample size during recent decade for studies using open dataset. **TabS15.** Sample size during recent three decades in the current study. **TabS16.** Summary for what modelswere built for neuropsychiatric diagnostic prediction in existing studies. **TabS17.** Summary for what cross-validationschemes were used to estimate model performance. **TabS18.** Summary for feature selection methods in existing studies. **TabS19.** Summary for what neural featureswere used in existing studies. **TabS20.** Summary for what pre-processing methods were used in existing studies. **TabS21.** Trends for the ratio of using open dataset on training ML models. **TabS22.** Results for comparison between SVM and DL classifiers on model performance. **TabS23.** Results for comparison between external validation CVCV) and otherson model performance. **TabS24.** Results for correlation between time and quality scores. **TabS25.** Study quality across psychiatric category.

**Additional file 2: Table S1.** Evidence table. This table is to summary the metadata and metainformation of all the included studies.

**Additional file 3: **Table S1-S2. **TabS1.** RNIMP 2022 Checklist. Reporting guideline and checklist for neuroimaging-based machine learning studies for psychiatry. **TabS2.** RNIMP 2022 workflow diagram. A workflow to guide for reporting neuroimaging-based machine learning models for psychiatry.

## Data Availability

The datasets generated and/or analyzed during the current study are available in the Open Science Framework (OSF) repository, https://osf.io/4zhsp/.

## Abbreviations

Acc: Accuracy
ADHD: Attention deficit/hyperactivity disorder
ARIMA: Autoregressive integrated moving average
ASD: Autism spectrum disorder
BP: Bipolar disorder
CV: Cross-validation
DALYs: Disability-adjusted life-years
GAM: Generalized additive model
GBD: Global Burden of Disease
GDP: Gross Domestic Product
GEE: Total Government Expenditure on public Education
HC: Healthy control
HDI: Human Development Index
LEDC (MEDC): Less (more) economic developed countries
LOOCV: Leave-one-out cross-validation
LSTM: Long short-term memory
MDD: Major depressive disorder
MHDB: Mental health disease burden
ML: Machine learning
OSF: Open Science Framework
R & D: Research and Development expenditure
ROB: Risk of bias
SZ: Schizophrenia
UHIC: High-income countries
WB: World Bank
WEIRD: Western, Educated, Industrialized, Rich, and Democratic
YLDs: Years lived with disability
