# Supplementary material for: Sampling inequalities affect generalization of neuroimaging-based diagnostic classifiers in psychiatry
Source: BMC Med. 2023 Jul 3;21:241. doi: 10.1186/s12916-023-02941-4 (PMC10318841; doi:10.1186/s12916-023-02941-4)
Supplement: Supplementary file 1 — Additional file 1: Figure S1-S8 and Table S1-S25. FigS1. Research pipelines for data acquisition. FigS2. PRISMA 2020 flow diagram for the current study. FigS3. Trends in ML-based diagnostic prediction for psychiatric diseases by neural features. FigS4. Mental health disorders as the portion of total disease burden at 2019. FigS5. Geospatial model for sampling population within China, Germanyand U.K. FigS6. Distribution of methodological details. FigS7. Model performance across algorithm, tookit, cross-validation, sample sizeand skewness. FigS8. Model performance across validations, trajectories, psychiatric categories, journal impacts, scanning technology/modalityand institutes/datasets. TabS1. Curve fitting results for exponential function model. TabS2. Journals counts for papers aiming at neuropsychiatric diagnostic prediction. TabS3. Counts for contributors’ sources for these papers. TabS4. Summary for sample population for these papers in the world. TabS5. Summary for sample population for these papers in the U.S. TabS6. Summary for sample population for these papers in the China. TabS7. Summary for sample population for these papers in the Germany. TabS8. Summary for sample population for these papers in the U.K. TabS9. Sampling inequalities for globe and countries/regions. TabS10. Sampling inequalities for continents. TabS11. Sampling inequalities and national development index. TabS12. Sample size during recent decade for all the studies. TabS13. Sample size during recent decade for studies using self-recruiting sample. TabS14. Sample size during recent decade for studies using open dataset. TabS15. Sample size during recent three decades in the current study. TabS16. Summary for what modelswere built for neuropsychiatric diagnostic prediction in existing studies. TabS17. Summary for what cross-validationschemes were used to estimate model performance. TabS18. Summary for feature selection methods in existing studies. TabS19. Summary for what neural featuresw [file 12916_2023_2941_MOESM1_ESM.pdf]

SUPPLEMENTAY MATERIALS: Sampling inequalities affect generalization of neuroimaging-based diagnostic classifiers in psychiatry

Zhiyi Chen[1,2*], Bowen Hu[1,2], Xuerong Liu[1], Benjamin Becker[3,4], Simon B. Eickhoff[5], Kuan Miao[1], Xingmei Gu[1], Yancheng Tang [6], Xin Dai[2], Chao Li[7], Artemiy Leonov[8], Zhibing Xiao[9], Zhengzhi Feng [1], Ji Chen[10,11*], Hu Chuan-Peng[12]

[1] Experimental Research Center for Medical and Psychological Science (ERC-MPS), School of Psychology, Third Military Medical University, Chongqing, China
[2] Faculty of Psychology, Southwest University, Chongqing, China
[3] The Center of Psychosomatic Medicine, Sichuan Provincial Center for Mental Health, Sichuan Provincial People's Hospital, Chengdu, China
[4] The Clinical Hospital of Chengdu Brain Science Institute, MOE Key Laboratory for Neuroinformation, University of Electronic Science and Technology of China, Chengdu, China
[5] Institute of Systems Neuroscience, Heinrich Heine University Düsseldorf, Düsseldorf, Germany
[6] School of Business and Management, Shanghai International Studies University, Shanghai, China
[7] Department of Radiology, The Third Affiliated Hospital, Sun Yat-Sen University, Guangdong, China
[8] School of Psychology, Clark University, Massachusetts, USA
[9] State Key Laboratory of Cognitive Neuroscience and Learning, Beijing Normal University, Beijing, China
[10] Department of Psychology and Behavioral Sciences, Zhejiang University, Hangzhou, China
[11] Department of Psychiatry, The Fourth Affiliated Hospital, Zhejiang University School of Medicine, Yiwu, Zhejiang, China;
[12] School of Psychology, Nanjing Normal University, Nanjing, China

* Corresponding authors: Zhiyi Chen (chenzhiyi@tmmu.edu.cn); Ji Chen (jichen.allen@hotmail.com)

Contents

SI Methods 1
SI Results 2
SI Supplementary Fig. 1 Research pipelines for data acquisition 4
SI Supplementary Fig. 2 PRISMA 2020 flow diagram for the current study 6
SI Supplementary Fig. 3 Trends in ML-based diagnostic prediction for psychiatric diseases by Neural features 7
SI Supplementary Fig. 4 Mental health disorders as the portion of total disease burden at 2019 (CC-BY) 8
SI Supplementary Fig. 5 Geospatial model for sampling population within China (A), Germany (B) and U.K (C).......... 9
SI Supplementary Fig. 6 Distribution of methodological details 10
SI Supplementary Fig. 7 Model performance across algorithm (A), tookit (B), cross-validation (C), sample size (D) and skewness (E) 11
SI Supplementary Fig. 8 Model performance across validations (A-B), trajectories (C), psychiatric categories (D), journal impacts (E), scanning technology/modality (F) and institutes/datasets (G).......... 13
SI Supplementary Tab. 1 Curve fitting results for exponential function model.......... 14
SI Supplementary Tab. 2 Journals counts for papers aiming at neuropsychiatric diagnostic prediction (classification) 15
SI Supplementary Tab. 3 Counts for contributors' sources for these papers 19
SI Supplementary Tab. 4 Summary for sample population for these papers in the world 20
SI Supplementary Tab. 5 Summary for sample population for these papers in the U.S 21
SI Supplementary Tab. 6 Summary for sample population for these papers in the China 22
SI Supplementary Tab. 7 Summary for sample population for these papers in the Germany 23
SI Supplementary Tab. 8 Summary for sample population for these papers in the U.K 24
SI Supplementary Tab. 9 Sampling inequalities for globe and countries/regions 25
SI Supplementary Tab. 10 Sampling inequalities for continents 26
SI Supplementary Tab. 11 Sampling inequalities and national development index 27
SI Supplementary Tab. 12 Sample size during recent decade for all the studies 28
SI Supplementary Tab. 13 Sample size during recent decade for studies using self-recruiting sample 29
SI Supplementary Tab. 14 Sample size during recent decade for studies using open dataset. 30
SI Supplementary Tab. 15 Sample size during recent three decades in the current study ... 31
SI Supplementary Tab. 16 Summary for what models (algorithms) were built for neuropsychiatric diagnostic prediction in existing studies 32
SI Supplementary Tab. 17 Summary for what cross-validation (CV) schemes were used to estimate model performance 34
SI Supplementary Tab. 18 Summary for feature selection methods in existing studies 35
SI Supplementary Tab. 19 Summary for what neural features (modality) were used in existing studies 38

SI Supplementary Tab. 20 Summary for what pre-processing methods were used in existing studies 39
SI Supplementary Tab. 21 Trends for the ratio of using open dataset on training ML models 40
SI Supplementary Tab. 22 Results for comparison between SVM and DL classifiers on model Performance 41
SI Supplementary Tab. 23 Results for comparison between external validation CV (leave-one-site-out CV and independent-samples (sites) CV) and others (i.e., k-fold, LOSO and hold-out CV) on model performance 42
SI Supplementary Tab. 24 Results for correlation between time and quality scores 43
SI Supplementary Tab. 25 Study quality across psychiatric category 44

Supplementary texts

SI Methods

ARIMA model

To examine the data stationarity, we used MATLAB to plot time series data for visualization inspection. Then, the Dickey-Fuller test and KPSS test have been done to examine data stationarity by statistical inferences. Further, the detrending and 1st differences-in-differences (DiD) processes have been used for non-stationarity correction. Subsequently, we have drawn the partial auto regressive function (PACF) and ACP plots to determine model parameters.

SI Results

Robust validation for association between sampling inequalities and national development index

We have validated the robustness and sensitivity for association between national development index and sampling inequalities by using parallel statistical models. Results showed significantly positive association between Gross Domestic Product (GDP) and sampling Gini coefficient in parametric model instead of non-parametric one ($r_{(Pearson)}$ = - .85, p = .004, BF_{10} = 13.57; $r_{(Spearman)}$ = - 0.65, p = .067, BF_{10} = 1.90). On the other hand, we found null association between sampling Gini coefficient and other national development index, including Human Development Index (HDI) ($r_{(Pearson)}$ = - .34, p = .372, BF_{01} = 1.72; $r_{(Spearman)}$ = - .03, p = .948, BF_{10} = 2.43), total government expenditure on public education (GEE) ($r_{(Pearson)}$ = - .21, p = .58, BF_{10} = 2.16; $r_{(Spearman)}$ = - .12, p = .776, BF_{10} = 2.43) and mental health diseases burden (MHDB) ($r_{(Pearson)}$ = .40, p = .289, BF_{10} = 1.50; $r_{(Spearman)}$ = .27, p = .490, BF_{10} = 1.79). Further, to obviate algorithmic pitfalls in Gini index, we re-calculated these association by using Theil index, and demonstrated similar results, with positive association to GDP ($r_{(Pearson)}$ = - .77, p = .014, BF_{10} = 5.36; $r_{(Spearman)}$ = - .65, p = .067, BF_{10} = 1.90) and null association to HDI ($r_{(Pearson)}$ = .15, p = .698, BF_{01} = 2.30; $r_{(Spearman)}$ = .03, p = .948, BF_{01} = 2.42), GEE ($r_{(Pearson)}$ = .05, p = .831, BF_{01} = 2.44; $r_{(Spearman)}$ = - .12, p = .776, BF_{01} = 2.43), R&D ($r_{(Pearson)}$ = - .08, p = .821, BF_{01} = 2.41; $r_{(Spearman)}$ = - .25, p = .521, BF_{01} = 2.05) and MHDB ($r_{(Pearson)}$ = .45, p = .223, BF_{01} = 1.27; $r_{(Spearman)}$ = .27, p = .493, BF_{01} = 1.80). In short, the results for the association between sampling inequalities and national development index were robust.

Permutation test for the association between sampling inequality and national income

We estimated the Pearson correlation coefficient for the association between sampling inequality and national income (i.e., GDP). By visual inspection, we controlled confounding factor from removing two outliers (i.e., China and the USA). We randomly shuffled labels of sampling Gini coefficients to generate pseudo-group. This process has been iterated for 1000 times to produce null distribution. As we assumed the positive direction for this correlation, the one-side statistical inference has been used here. It should be in mind that the p value is found to marginally reach significance (p = 0.08).

Robust validation for the association between time and sample size

Likewise, we also used Spearman correlation to perform robustness validation for the association between time and sample size. Supporting that, we found the significantly positive correlation between time and averaged sample size for all the existing studies (Pearson model, $r_{(total)}$ = .75, 95 % CI: .22 - 0.93, p = .013; BF_{10} = 5.83, Strong evidence; Spearman model, $r_{(total)}$ = .79, 95 % CI: .33 - 0.95, p = .010; BF_{10} = 11, Strong evidence). In addition, we also examined this association by using median value, and revealed the same relationship ($n_{(Median, 2011)}$ = 40, $n_{(Mean, 2020)}$ = 128, $r_{(total)}$ = .86, 95 % CI: .50 - 0.97, p = .001; BF_{10} = 23.40, Strong evidence). In total, the positive linear association between time and sample size was robust in the current study.

K-fold statistics

To tackle with unbalanced number of two groups for comparisons towards model performance,

we used k-fold scheme for down-sampling towards group which has high volume of cases. With respect to algorithm (classifier), we randomly down-sampled SVM group into four folds. Consistent findings were observed in all the folds: the accuracy for SVM classifiers was higher than DL ones (see Supplementary Tab. 22). Likewise, the same solution (i.e., down-sampling whole sample into six folds) was used for comparison for model performance with regards to external validation CV (i.e., leave-one-site-out CV and independent-samples (sites) CV) and others (i.e., k-fold, LOSO and hold-out CV). We observed the model performance was lower in external validation CV than of others in all the folds as well (see Supplementary Tab. 23).

Supplementary Figures

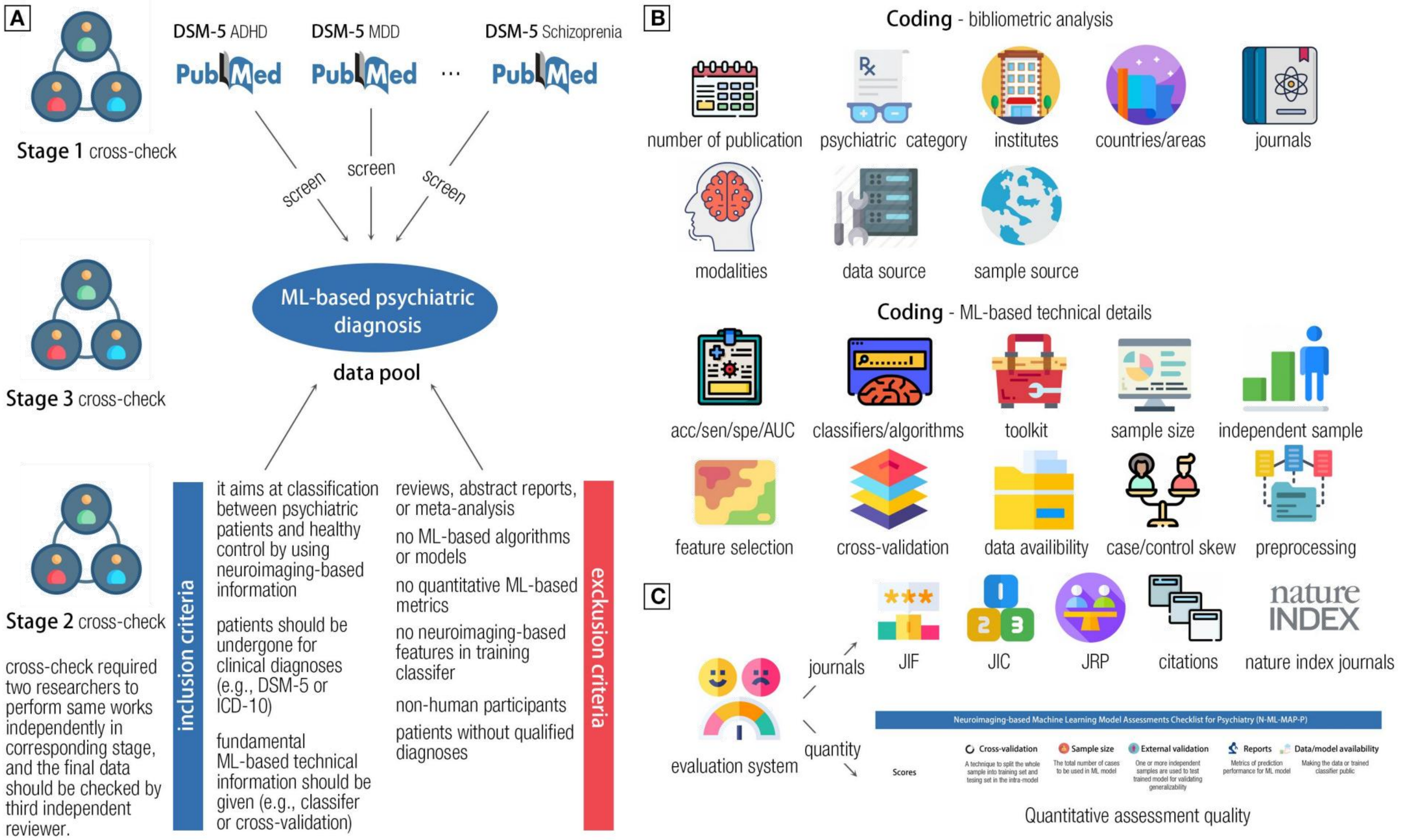

Supplementary Fig. 1 Research pipelines for data acquisition. (A) presents literature searching procedure in accordance with PRISMA 2020, and details inclusion and exclusion; each stage required cross-check processing; (B) shows what metainformation we wanna code in bibliometric analysis; (C) shows what evaluation system we built to probe into the association between study quality and these machine-learning metrics (e.g., acc, AUC, CV schemes).

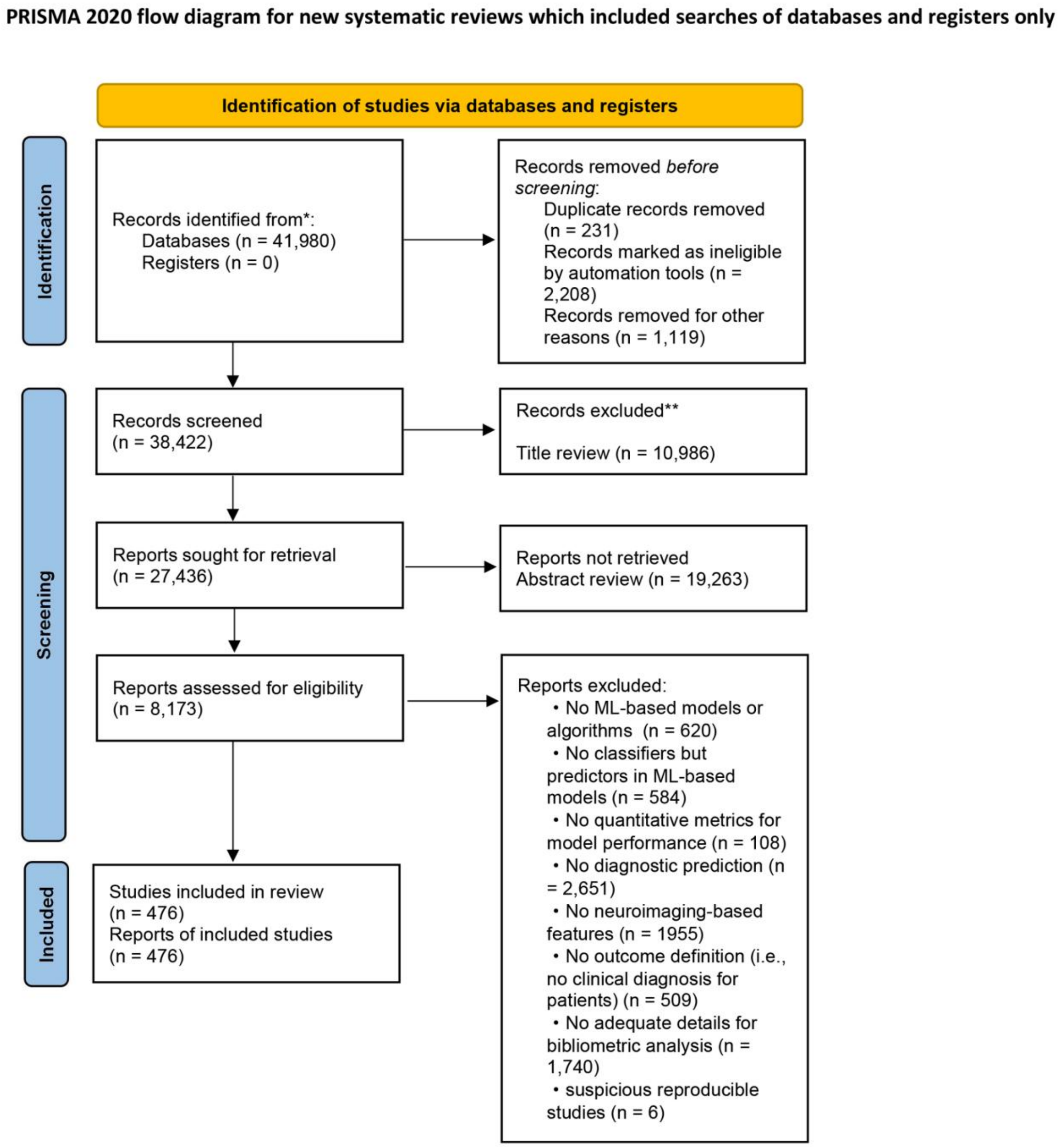

Supplementary Fig. 2 PRISMA 2020 flow diagram for the current study.

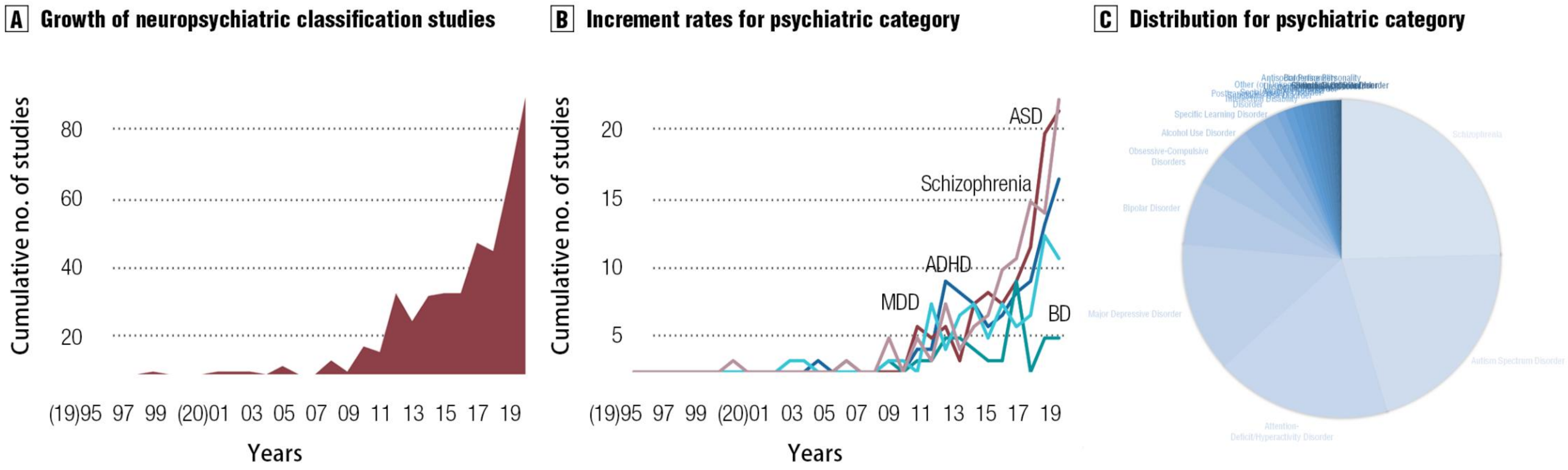

Supplementary Fig. 3 Trends in ML-based diagnostic prediction for psychiatric diseases by neural features. (A) describes increased number of studies for relevant studies during recent three decades (1990-2020); (B) plots increment rates of existing studies concerning ML-based psychiatric diagnostic prediction towards ADHD, MDD, SZ and BD; (C) provides a pie plot to show the distribution pattern for psychiatric categories.

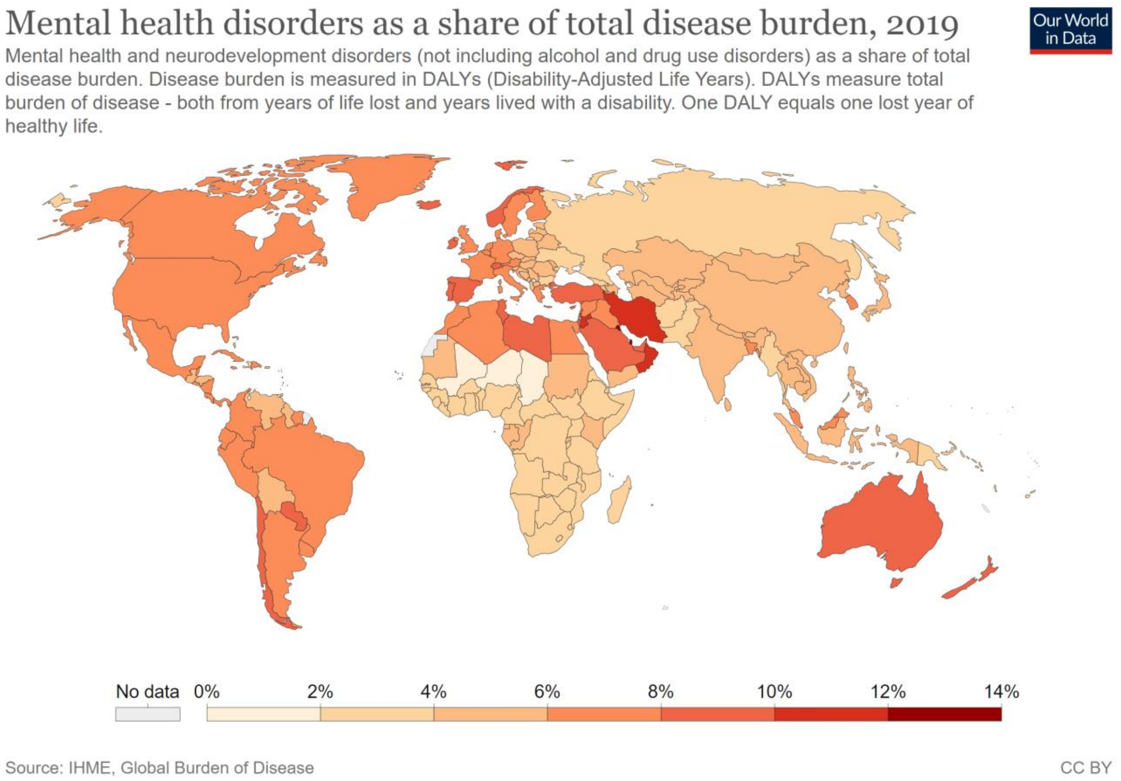

Supplementary Fig. 4 Mental health disorders as the portion of total disease burden at 2019 (CC-BY). Data and map is drawn basing on Our world in data.

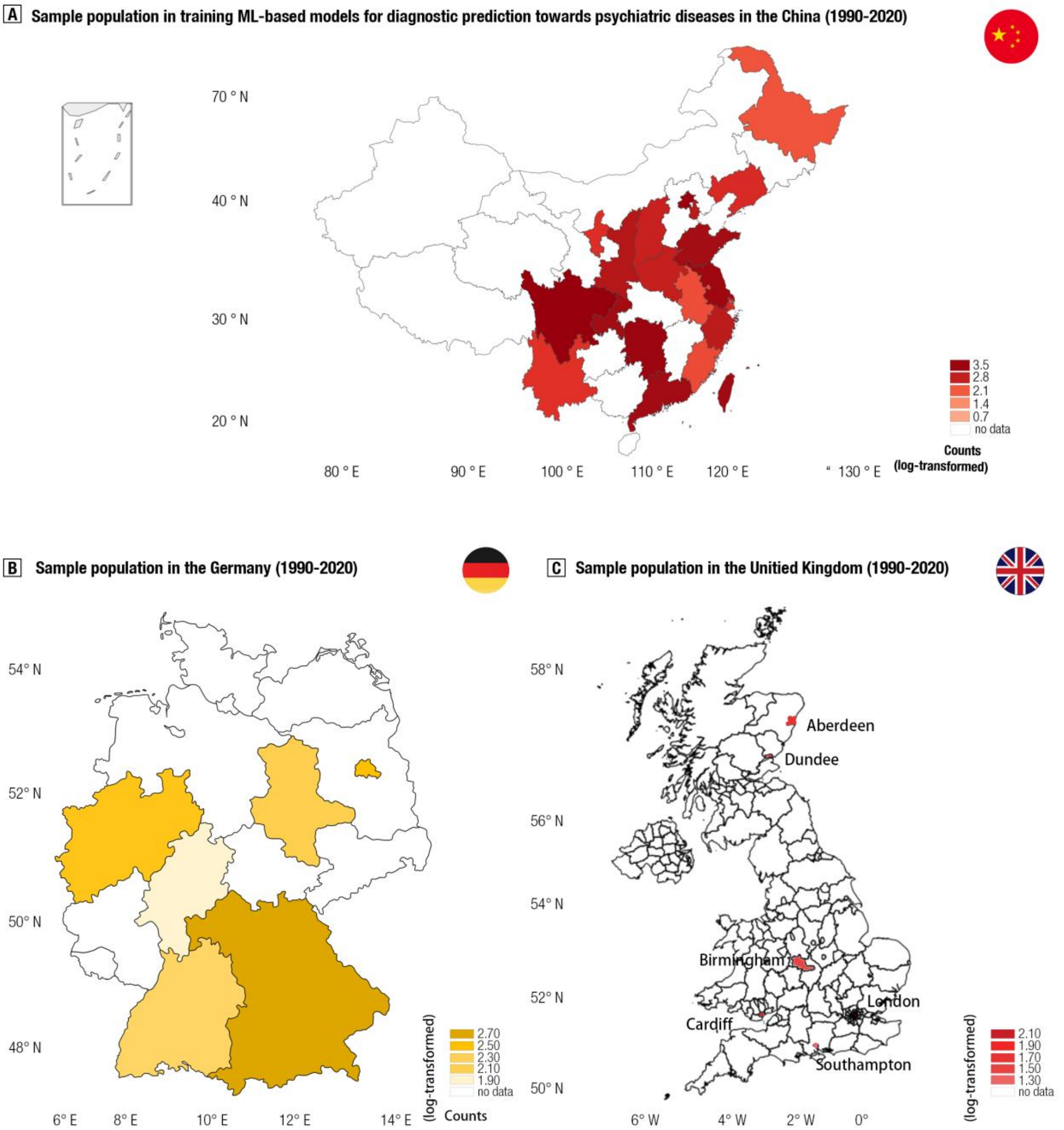

Supplementary Fig. 5 Geospatial model for sampling population within China (A), Germany (B) and U.K (C). For readable visualization, the number of participants (sampling population) has been transformed by log functions. Projecting U.K. samples used second-level (i.e. county) administrative fine-grained map.

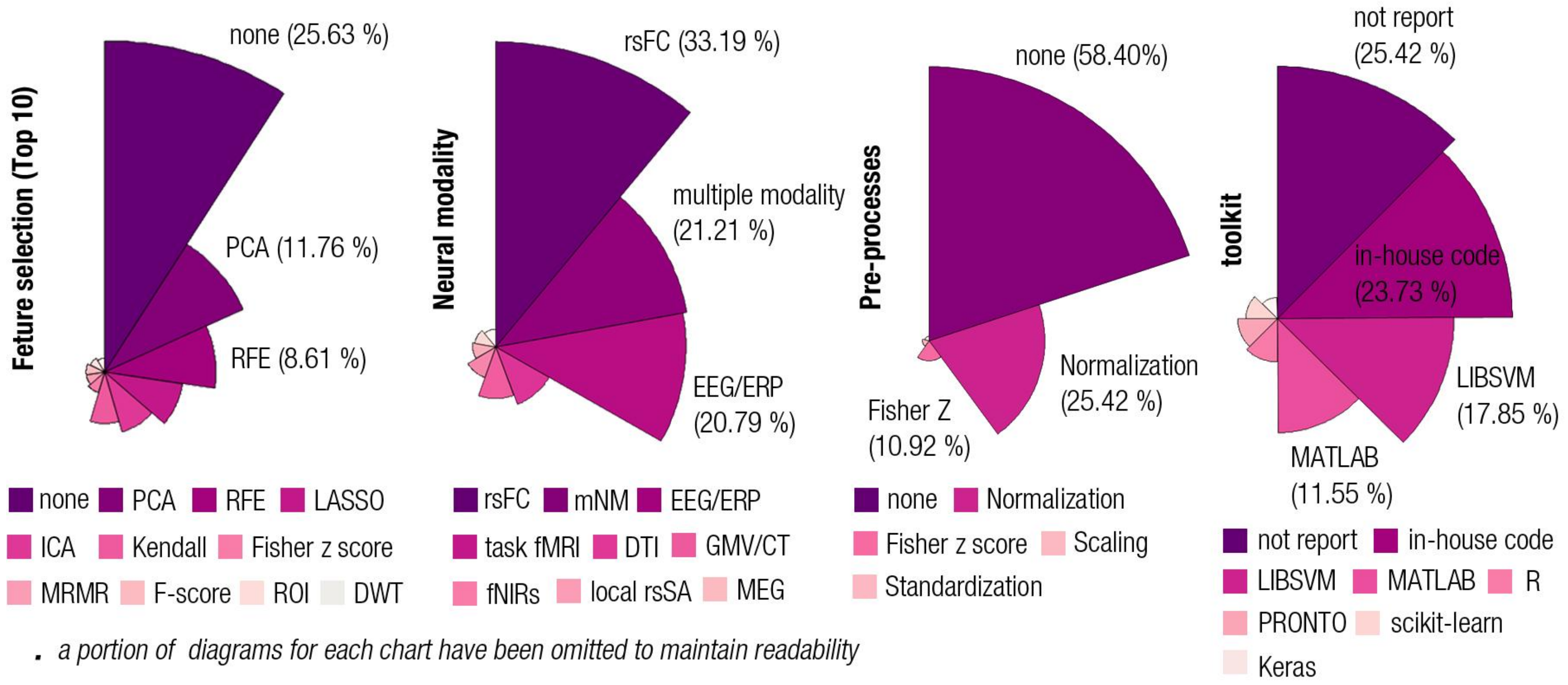

Supplementary Fig. 6 Distribution of methodological details. These panels show a portion of methodological parameters (Top 10%) for reliability. Full results can be found in Supplementary Tables.

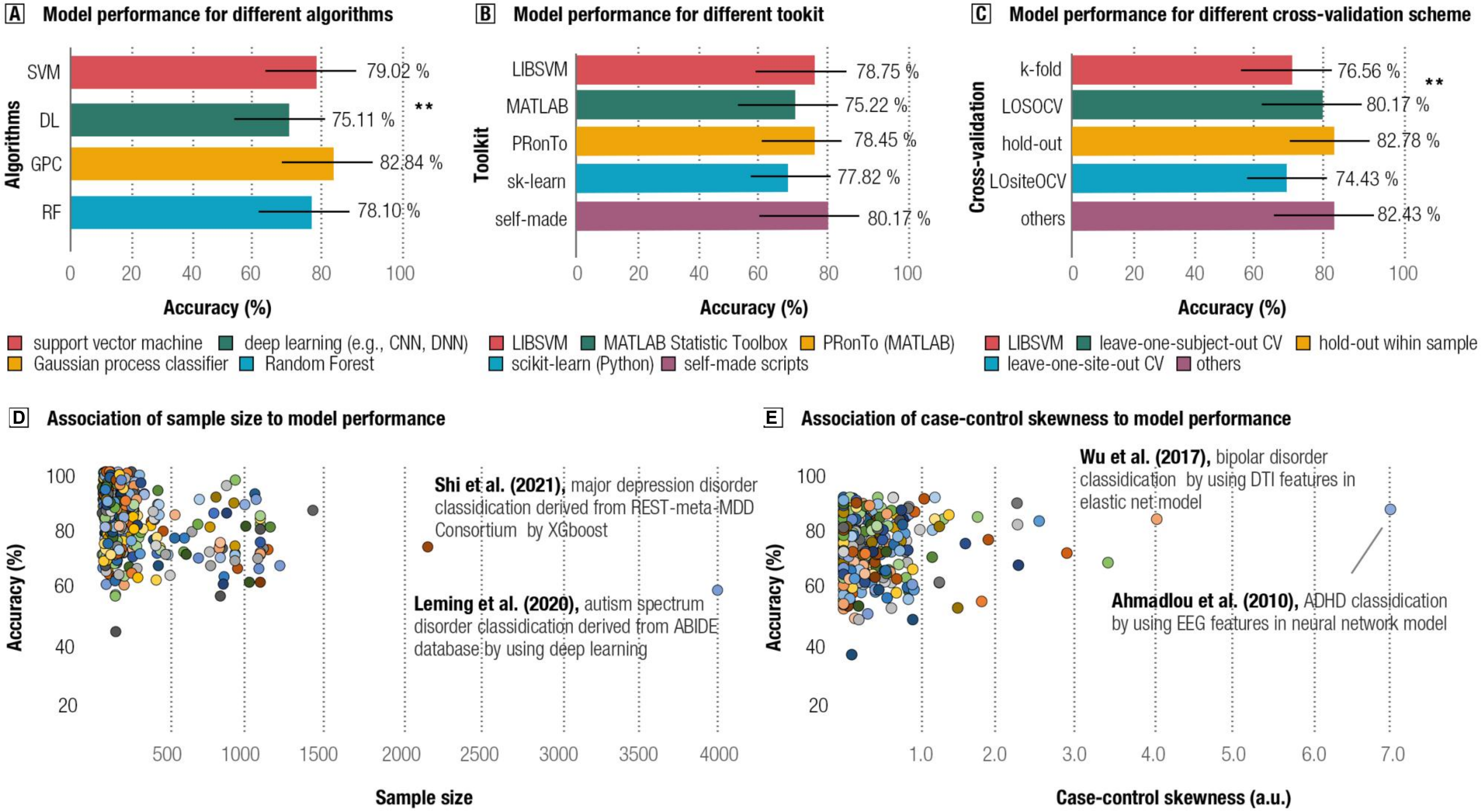

Supplementary Fig. 7 Model performance across algorithm (A), tookit (B), cross-validation (C), sample size (D) and skewness (E). Precision-weighted method

was used to adjust model accuracy. Non-parametric statistics were conducted for comparison across methodological schemes (i.e., algorithm and cross-validation schemes). * < .05; ** <.01; *** <.001.

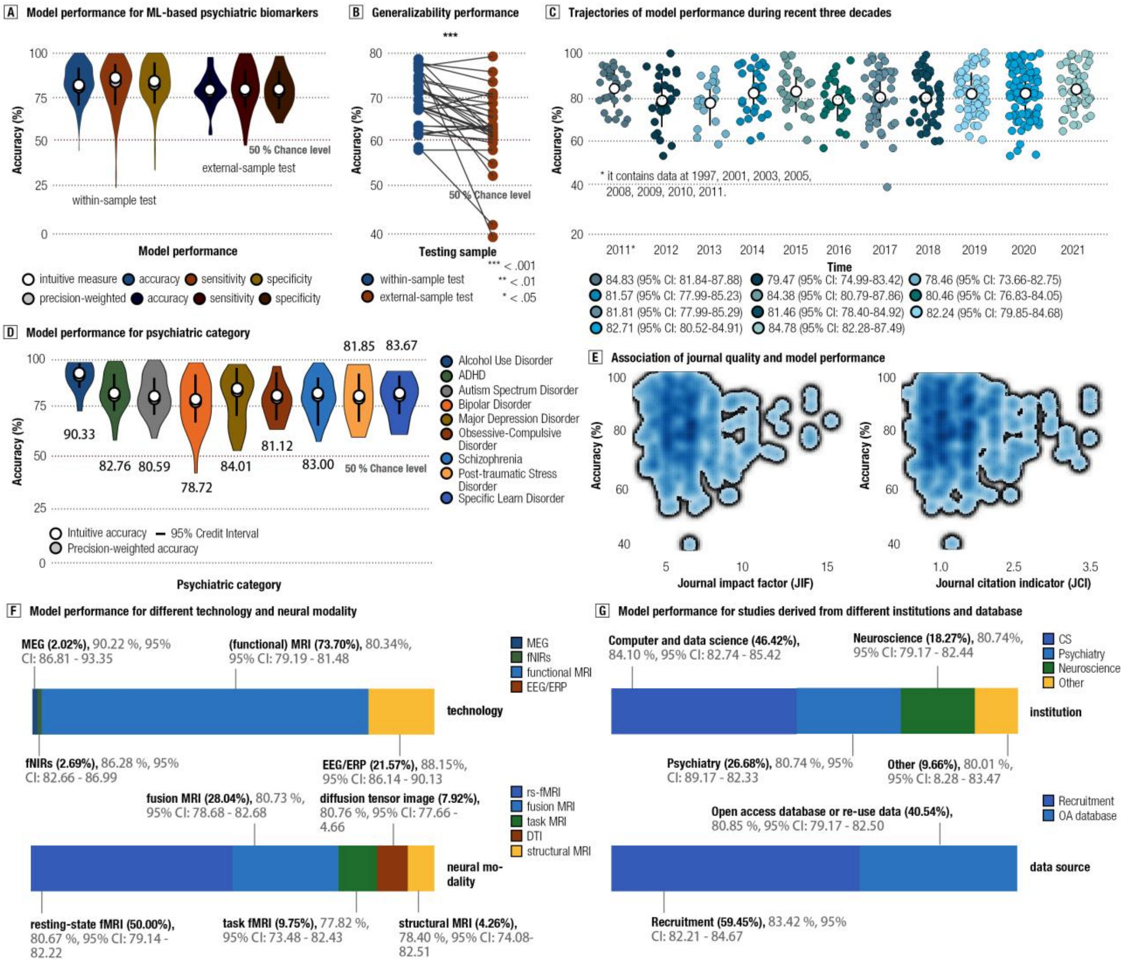

Supplementary Fig. 8 Model performance across validations (A-B), trajectories (C), psychiatric categories (D), journal impacts (E), scanning technology/modality (F) and institutes/datasets (G). Precision-weighted method was used to adjust model accuracy. Both intuitive measures and precision-weighted measures for estimating model performance for all the studies validating in the internal, which showed accuracy of 82.69 % (precision-weighted, 79.01%; sensitivity of 82.08%, 77.48 (weighted); specificity of 82.39%, 78.40%). Panel B showed that model performance estimated by external sample is significantly declined, which indicated the poor generalizability for these models. Panel C shows no prominent trends for model performance are found, which lead us to imply that the model performance for predicting psychiatric disorders is not improved substantially during recent 30 years. Panel D shows no significantly deviation for model performance towards different disorders excepting alcohol use disorder in visual inspection. Panel E shows no significant association between model performance and journal quality (measured by IF and JCI). All the panels show the precision-weighted measures without statistical inferences given the unbalanced sample sizes.

Category	b value	95 % CI	SSE	R^2	Adjust R^2	DFE	RMSE
Schizophrenia	2.395	2.054 - 2.742	43.13	0.956	0.954	29	1.219
Major Depression Disorder	1.609	1.138 - 2.079	55.57	0.819	0.812	29	1.384
Bipolar Disorder	1.178	0.353 - 2.002	49.30	0.423	0.403	29	1.304
Autism Spectrum Disorder	2.637	2.252 - 3.023	45.80	0.956	0.954	29	1.256
attention deficit/ hyperactivity disorder	1.895	1.470 - 2.319	60.96	0.891	0.887	29	1.499

Supplementary Tab. 1 Curve fitting results for exponential function model. All the data have been undergone centering.

Journals	Counts
Plos one	28
NeuroImage: Clinical	22
Human Brain Mapping	20
Neuroimage	20
Front Neurosci	16
Front Psychiatry	13
Schizophrenia research	13
Frontiers in Human Neuroscience	10
Journal of affective disorders	9
Scientific Report	9
Annu Int Conf IEEE Eng Med Biol Soc	8
Brain imaging and behavior	8
Front Syst Neurosci	8
Psychological Medicine	8
Computer Methods and Programs in Biomedicine	7
IEEE TRANSACTIONS ON NEURAL SYSTEMS AND REHABILITATION ENGINEERING	7
Journal of neuroscience methods	7
Psychiatry Research: Neuroimaging	7
Clinical Neurophysiology	6
Sensors	6
Transl Psychiatry	6
Clinical EEG and Neuroscience	5
Artificial Intelligence in Medicine	5
BMC Psychiatry	5
J Neural Eng	5
Annu Int Conf IEEE Eng Med Biol Soc	4
Brain sciences	4
Computerized Medical Imaging and Graphics	4
Neuroscience Letters	4
Schizophrenia Bulletin	4
Biol Psychiatry Cogn Neurosci Neuroimaging	3
BioMed research international	3
Bipolar Disord	3
Brain	3
Brain and behavior	3
Brain connectivity	3
Computational and Mathematical Methods in Medicine	3
Conf Proc IEEE Eng Med Biol Soc	3
Cortex	3
IEEE Trans Biomed Eng	3
International Journal of Neural Systems	3
J Neural Transm (Vienna)	3

Journal of digital imaging	3
Journal of Medical Systems	3
Medical & Biological Engineering & Computing	3
Medical image computing and computer-assisted intervention	3
Neuroreport	3
NPJ Schizophr	3
Physical and Engineering Sciences in Medicine	3
Psychiatry and clinical neurosciences	3
Psychiatry Research	3
2018 40th Annual International Conference of the IEEE Engineering in Medicine and Biology Society (EMBC)	2
Acta Psychiatrica Scandinavia	2
Addict Biol	2
Autism Research	2
Biol Psychiatry	2
Biomed Engineering Online	2
Br J Psychiatry	2
Cerebral Cortex	2
Cognitive Neurodynamics	2
EBioMedicine	2
Eur Child Adolesc Psychiatry	2
European Archives of Psychiatry and Clinical Neuroscience	2
Front Neuroinform.	2
Frontiers in computational neuroscience	2
IEEE TRANSACTIONS ON CYBERNETICS	2
Int J Methods Psychiatr Res	2
International Journal of Psychophysiology	2
International Journal of Neural Systems	2
J Clin Med	2
JAMA Psychiatry	2
Journal of Attention Disorders	2
Journal of Child Psychology and Psychiatry	2
Journal of Neurodevelopmental Disorders	2
Magnetic Resonance Imaging	2
Neural Networks	2
Neuropsychopharmacology	2
Nonlinear biomedical physics	2
Progress in Neuro-Psychopharmacology and Biology pathways	2
2014 36th Annual International Conference of the IEEE Engineering in Medicine and Biology Society	1
2020 42nd Annual International Conference of the IEEE Engineering in Medicine & Biology Society (EMBC)	1
ACS Chemical Neuroscience	1
Acta Neuropsychiatr	1

AIMS Neurosci	1
Ann Dyslexia	1
Australian & New Zealand Journal of Psychiatry	1
Basic Clin Neurosci.	1
Behav Brain Res	1
Behav Neurol	1
Bio-Medical Materials and Engineering	1
Biological Psychology	1
BMC Bioinformatics	1
BMC medicine	1
BMC Neurosci	1
BMC Med inform decision making	1
BMC Neurology	1
BMJ open	1
BOOK	1
Brain research	1
Brain Struct Funct	1
Brain Topogr	1
Chaos	1
Children (Basel)	1
Clin Neurol Neurosurg	1
Clinical psychological science	1
Computational Intelligence and Neuroscience	1
Computers in Biology and Medicine	1
Current Biology	1
Disease Markers	1
Entropy	1
Epilepsia	1
Eur J Radiol	1
Eur Neuropsychopharmacol	1
Experimental neurobiology	1
Front Physiol.	1
Frontiers in neural circuits	1
Heliyon	1
ieee access	1
IEEE J Biomed Health Inform	1
IEEE J Transl Eng Health Med	1
IEEE Transactions on Neural Networks and Learning Systems	1
Int J Eat Disord.	1
International journal of environmental research and public health	1
International journal of geriatric psychiatry	1
J Magn Reson Imaging	1
J Neuroimaging	1
Journal of Clinical Neurophysiology	1

JOURNAL OF COMPUTATIONAL BIOLOGY	1
Journal of Integrative Neuroscience	1
Journal of Medical Signals and Sensors	1
Journal of personalized medicine	1
Journal of Psychiatric Research	1
Journal of the American Academy of Child & Adolescent Psychiatry	1
Lancet Psychiatry	1
Med Eng Phys	1
Medical Image Analysis	1
Medical physics	1
Medicine (Baltimore)	1
Molecular Autism	1
Nat Med	1
Nature communication	1
Neural Plast	1
Neuroinformatics	1
Neurophotonics	1
Neuropsychiatr Dis Treat.	1
Neuropsychiatr Electrophysiol	1
Neuroscience	1
PeerJ	1
Physiol Meas	1
PLOS BIOL	1
Proc IEEE Int Symp Biomed Imaging.	1
Proc Inst Mech Eng H	1
Psychophysiology	1
Radiol Artif Intell	1
Radiology	1
Social cognitive and affective neuroscience	1
Social Neuroscience	1
World J Biol Psychiatry	1

Supplementary Tab. 2 Journals counts for papers aiming at neuropsychiatric diagnostic prediction (classification).

Countries or regions	Counts
China	136
USA	102
Canada	24
Korea	23
UK	21
Germany	20
Iran	17
Japan	16
Spain	13
Italy	12
Brazil	9
Singapore	8
Taiwan	8
Malaysia	7
Switzerland	7
India	6
Netherlands	6
Turkey	6
Australia	5
Czech	3
Greece	3
Norway	3
Chilie	2
Iceland	2
Saudi Arabia	2
Sweden	2
Belgium	1
Cyprus	1
Denmark	1
Finland	1
France	1
Hungary	1
Ireland	1
Israel	1
Poland	1
Portugal	1
Republic of Macedonia	1
Romania	1
South Africa	1

Supplementary Tab. 3 Counts for contributors' sources for these papers.

Countries or regions	No. of studies	No. of participants
China	121	14869
USA	134	12024
Germany	17	4330
Japan	10	2935
Korea	15	1744
Italy	13	1466
Taiwan	8	884
Czech	3	864
Norway	3	848
Canada	9	819
Switzerland	6	785
Netherlands	8	670
Spain	10	641
Iceland	1	630
UK	13	605
Brazil	5	584
Malaysia	8	439
Iran	10	401
Turkey	6	394
India	4	362
Australia	7	306
Singapore	2	188
Ireland	3	187
Macedonia	1	177
Hungary	1	145
Romania	1	128
Denmark	1	104
Belgium	3	92
Jordan	1	77
Sweden	2	71
Columbia	1	60
France	1	56
Greece	2	54
Poland	2	28
Saudi Arabia	2	19

Supplementary Tab. 4 Summary for sample population for these papers in the world. No. of participants have been adjusted by reproducible counts, such as reuse data and open data repository.

Sample site in U.S.	No. of studies	No. of participants
California	30	2326
Pennsylvania	9	1897
New York	10	1063
Illinois	7	990
Connecticut	13	964
Maryland	7	951
Texas	9	829
Massachusetts	6	541
Missouri	2	526
Kentucky	4	511
New Mexico	2	407
Michigan	5	367
Washington	4	342
Utah	3	214
Oregon	3	200
Minnesota	5	199
Indiana	2	88
Rhode Island	2	83
North Carolina	2	80
New Jersey	1	72
Ohio	1	50
Colorado	1	48
Georgia	1	40
Tennessee	1	30
Alabama	1	27

Supplementary Tab. 5 Summary for sample population for these papers in the U.S. No. of participants have been adjusted by reproducible counts, such as reuse data and open data repository.

Sample site in China (including Taiwan area)	No. of studies	No. of participants
Beijing	17	4515
Sichuan	15	2208
Hunan	20	1382
JiangSu	14	1161
Taiwan	8	884
Chongqing	4	723
Shandong	2	673
GuangDong	5	630
ShanXi(South)	1	414
Tianjing	3	361
Zhejiang	2	354
Henan	3	300
ShanXi(North)	6	281
Liaoning	1	189
Shanghai	7	185
Ningxia	2	165
Yunnan	2	158
Fujian	1	90
Anhui	1	87
Heilongjiang	1	75

Supplementary Tab. 6 Summary for sample population for these papers in the China (including Taiwan area) No. of participants have been adjusted by reproducible counts, such as reuse data and open data repository.

Sample site in Germany	No. of studies	No. of participants
Jülich	3	918
Berlin	4	601
Aachen	2	378
Dresden	1	254
Heidelberg	1	154
Frankfurt am Main	1	21

Supplementary Tab. 7 Summary for sample population for these papers in the Germany No. of participants have been adjusted by reproducible counts, such as reuse data and open data repository.

Sample site in U.K.	No. of studies	No. of participants
London	8	388
Cardiff	1	83
Aberdeen/Edinburgh	1	62
Dundee	1	41
Birmingham	1	37
Southampton	1	24

Supplementary Tab. 8 Summary for sample population for these papers in the U.K. No. of participants have been adjusted by reproducible counts, such as reuse data and open data repository.

Country (Regions)	Gini coefficient	Theil index	P value
Globe	0.810	1.537	< .001
LEDC	0.936	1.885	< .001
MEDC	0.334	0.408	< .001
Iran	0.922	2.342	< .001
Germany	0.784	1.296	< .001
China	0.471	0.548	< .001
Italy	0.742	1.236	< .001
Japan	0.915	2.242	< .001
Korea	0.855	1.732	< .001
Spain	0.912	2.204	< .001
U.K.	0.876	1.991	< .001
U.S.	0.577	0.742	< .001

Supplementary Tab. 9 Sampling inequalities for globe and countries/regions. Permutation test is used for statistical inference to compared with null distribution. LEDC = Less Economic Development Countries; MEDC = More Economic Development Countries; U.K. = United Kingdom; U.S. = United States

Continents	No. of participants	Total population	Proportion of sample	Gini coefficient
Asia	21,860	41.64×10^{8}	0.52×10^{-5}	0.826
Europe	11,134	7.40×10^{8}	0.15×10^{-5}	0.636
North America	12,843	5.28×10^{8}	0.24×10^{-5}	0.920
South America	1,636	4.34×10^{8}	0.03×10^{-5}	0.886
Oceania	306	0.29×10^{8}	1.05×10^{-5}	0.937
Africa	-	-	-	-

Supplementary Tab. 10 Sampling inequalities for continents. Proportion of sample was used to adjust the number of participants who used for training ML models by total number of population in each continent. Total number of population in each continent was referred from Department of Economic and Social Affairs at United Nations (UN) (2019 Revision of World Population Prospects, https://population.un.org/wpp/)

Countries	Gini	Theil	GDP	HDI	GEE (%)	MHDB (%)	R&D (%)
Iran	0.922	2.342	0.203	0.783	3.96	10.31	0.83
Germany	0.784	1.296	3.85	0.947	4.91	6.43	3.13
China	0.471	0.548	14.72	0.761	3.51	5.3	2.14
Italy	0.742	1.236	1.89	0.892	4.04	7.15	1.39
Japan	0.915	2.242	5.06	0.919	3.18	4.91	3.28
Korea	0.855	1.732	1.64	0.916	5.31	5.23	4.53
Spain	0.912	2.204	1.28	0.904	4.21	8.68	1.24
U.K.	0.876	1.991	2.76	0.932	5.44	7.12	1.7
U.S.	0.577	0.742	20.95	0.926	4.391	6.56	2.83

Supplementary Tab. 11 Sampling inequalities and national development index. GDP = Gross Domestic Product; HDI = Human Development Index; GEE = total government expenditure on public education; MHDB = mental health diseases burden; R & D = research and development expenditure.

Time	Median	Mean	S-W test	Min	Max	Skew
2011	40.00	53.38	0.87***	10	105	1.30
2012	68.00	222.17	0.68***	24	1026	0.58
2013	54.00	100.87	0.33***	24	964	4.47
2014	59.75	102.93	0.68***	23	450	2.07
2015	48.00	136.46	0.54***	10	1008	0.95
2016	104.00	177.01	0.62***	22	888	0.46
2017	74.00	139.51	0.63***	17	1032	3.47
2018	125.25	238.62	0.72***	14	941	1.63
2019	108.00	238.10	0.59***	12	2004	2.91
2020	128.00	394.33	0.53***	19	4372	4.14
$r_{(Pearson)}$	0.860***	0.748*				
BF_{10}	31.38	5.826				
$r_{(Spearman)}$	0.867**	0.794**				
BF_{10}	11.203	11.203				

Supplementary Tab. 12 Sample size during recent decade for all the studies. S-W test means Shapiro-Wilk test to examine whether the distribution of data is in accordance with Gaussian shape. * $p < .05$, ** $p < .01$, *** $p < .001$.

Time	Median	Mean	S-W test	Min	Max	Skew
2011	40.00	54.29	0.85**	10	150	1.24
2012	53.00	60.53	0.84***	24	113	0.67
2013	50.00	50.24	0.93	24	82	0.05
2014	53.00	56.76	0.85*	24	132	1.43
2015	40.00	75.90	0.38***	10	630	4.32
2016	74.00	92.78	0.90*	22	216	0.73
2017	64.00	111.00	0.75***	17	374	1.49
2018	79.00	94.41	0.90	14	225	1.14
2019	92.50	115.43	0.51***	12	935	4.81
2020	95.00	168.38	0.66***	20	1100	2.85
r (Pearson)	0.891***	0.901***				
BF_{10}	87.66	87.66				
r (Spearman)	0.872***	0.822***				
BF_{10}	44.01	44.01				

Supplementary Tab. 13 Sample size during recent decade for studies using self-recruiting sample. S-W test means Shapiro-Wilk test to examine whether the distribution of data is in accordance with Gaussian shape. * p < .05, ** p < .01, *** p < .001.

Time	Median	Mean	S-W test	Min	Max	Skew
2011	53.50	50.50	0.89	20	75	-0.31
2012	626.00	544.20	0.92	58	1026	-0.33
2013	90.00	251.60	0.61***	46	964	2.21
2014	94.00	178.09	0.86	23	450	0.66
2015	173.00	306.12	0.77	38	1008	1.61
2016	180.50	344.10	0.78	60	888	0.92
2017	114.00	189.00	0.65***	19	1032	2.80
2018	193.00	333.59	0.81***	24	941	0.89
2019	222.00	459.04	0.78***	30	2004	1.62
2020	219.00	603.63	0.61***	19	4372	3.11
r (Pearson)	0.890***	0.858***				
BF_{10}	32.26	16.78				
r (Spearman)	0.833***	0.722**				
BF_{10}	26.03	9.57				

Supplementary Tab. 14 Sample size during recent decade for studies using open dataset. S-W test means Shapiro-Wilk test to examine whether the distribution of data is in accordance with Gaussian shape. * p < .05, ** p < .01, *** p < .001.

Sample size (No. of participants)	No. of Studies	Proportion (%)
<100	248	52.94
101-200	100	21.05
201-300	38	7.36
301-400	21	4.42
401-500	6	1.26
501-600	7	1.47
601-700	4	0.84
701-800	14	2.94
801-900	9	1.83
901-1000	4	0.84
>1000	24	5.05

Supplementary Tab. 15 Sample size during recent three decades in the current study.

Algorithms (Classifiers)	No. of Studies	Proportion (%)
Support Vector Machine, SVM	254	53.36134454
Convolutional Neural Network, CNN	37	7.773109244
Random Forest, RF	19	3.991596639
Gaussian Process Classifier, GPC	15	3.151260504
Linear Discriminant Analysis, LDA	14	2.941176471
Artificial Neural Network, ANN	10	2.100840336
Clustering	10	2.100840336
Deep Neural Network, DNN	10	2.100840336
Logistic Regression Classifier, LRC	10	2.100840336
K-Nearest Neighbor, KNN	5	1.050420168
LASSO Classifier	5	1.050420168
Self-made Unnamed Classifier	5	1.050420168
Decision Tree, DT	4	0.840336134
Probabilistic Neural Network, PNN	4	0.840336134
Relevant Vector Machine, RVM	4	0.840336134
XGBoost	4	0.840336134
Deep Brief Network, DBN	3	0.630252101
Recursive Neural Network, RNN	3	0.630252101
Convolutional Denoising Autoencoder, CDAE	2	0.420168067
Elastic Net, EN	2	0.420168067
Graph Convolutional Networks, GCN	2	0.420168067
Quadratic Discriminant Analysis, QDA	2	0.420168067
Short-term Memory Network, LSTM	2	0.420168067
Simple Linear Regression, SLR	2	0.420168067
AlexNet	1	0.210084034
ASD-DiagNet	1	0.210084034
Discriminant Deep Learning, DANS	1	0.210084034
Deep Autoencoder, DA	1	0.210084034
Deep Transfer Learning Neural Network, DTLNN	1	0.210084034
Deep Learning, DL	1	0.210084034
DL-DeepfMRI	1	0.210084034
DL-EEGNet	1	0.210084034
Discriminative Restricted Boltzmann machines, DRBM	1	0.210084034
Dual Subspace Learning, DSL	1	0.210084034
Empirical Mode Decomposition, EBT	1	0.210084034
Extreme Learning Machine, EML	1	0.210084034
EMPaSchiz	1	0.210084034
Ensemble	1	0.210084034

Generative Adversarial Networks, GAN	1	0.210084034
Gradient Boosting Decision Tree, GBDT	1	0.210084034
Gaussian Mixed Model, GMM	1	0.210084034
Graph Neural Network, GNN	1	0.210084034
Hierarchical Clustering, HC	1	0.210084034
Nonlinear Manifold Learning Algorithms, ISOMAP	1	0.210084034
Kernel Discriminant Analysis, KDA	1	0.210084034
L1-norm Regularized Sparse Canonical Correlation Analysis, L1-SCCA	1	0.210084034
L2-norm Linear Regression, L2-LR	1	0.210084034
Automatic Bayesian Classification, ABC	1	0.210084034
Locally Linear Embedding, LLE	1	0.210084034
Multiple Kernel Learning Classifier, MKL	1	0.210084034
Multiple Learning Process , MLP	1	0.210084034
Modified Adaboost Classification, MAC	1	0.210084034
Multistage Algorithm, MA	2	0.420168067
Unnamed Neural network	1	0.210084034
PBL-McRBFN	1	0.210084034
Penalized Regression Model, PRM	1	0.210084034
Radial Basis Function Neural Network, RBFNN	2	0.420168067
ResNet-50	1	0.210084034
Robust interdependence measure, RIM	1	0.210084034
Sparse hypergraph learning, STM	1	0.210084034
Symmetrical Uncertainty, SU	1	0.210084034
Transductive Classifier, TC	1	0.210084034
Unambiguous Component with Maximum Correlation, UMAX	1	0.210084034

Supplementary Tab. 16 Summary for what models (algorithms) were built for neuropsychiatric diagnostic prediction in existing studies.

Cross-validation scheme	No. of Studies	Proportion (%)
10-fold CV	132	27.7310924
5-fold CV	36	7.5630252
k-fold CV (n $\neq$ 5 or 10)	15	3.1512605
hold-out CV	24	5.0420168
LORO CV	1	0.210084
LOPO CV	12	2.5210084
LOsiteO CV	14	2.9411765
LOSO CV	182	38.2352941
nested 10-fold CV	11	2.3109244
nested 5-fold CV	1	0.0210084
nested k-fold CV	6	1.2605042
nested LOSO CV	10	2.1008403
Others	34	7.1428571

Supplementary Tab. 17 Summary for what cross-validation (CV) schemes were used to estimate model performance. LORO = leave-one-run-out; LOPO = leave-one-pair-out; LOsiteO = leave-one-site-out; LOSO = leave-one-subject-out.

Feature selection	No. of Studies	Proportion (%)
No feature selection	122	25.6302521
Univariate analysis	56	11.76470588
PCA	41	8.613445378
RFE	29	6.092436975
LASSO	23	4.831932773
ICA	19	3.991596639
Kendall tau rank	8	1.680672269
Fisher z score	7	1.470588235
MRMR	7	1.470588235
F-score	6	1.260504202
ROI	5	1.050420168
Discrete wavelet transforms	4	0.840336134
L2	4	0.840336134
Minimum redundancy and maximum relevance (mRMR)	4	0.840336134
Rank-based feature selection	4	0.840336134
Autoencoder (AE)	3	0.630252101
Dual Regression ICA	3	0.630252101
Elastic Net	3	0.630252101
Forward stepwise analyses	3	0.630252101
GLM	3	0.630252101
Max-pooling	3	0.630252101
Sequential forward feature selection (SFFS)	3	0.630252101
SVD	3	0.630252101
Wrapping	3	0.630252101
Genetic algorithm	2	0.420168067
GGLM	2	0.420168067
K-S test	2	0.420168067
LDA	2	0.420168067
Relief algorithm	2	0.420168067
Self-made	2	0.420168067
SVM	2	0.420168067
2-stage PCA	1	0.210084034
2D convolution kernel	1	0.210084034
AM_FM	1	0.210084034
ApEn	1	0.210084034
AR	1	0.210084034
Backward elimination	1	0.210084034
Block	1	0.210084034
BSL	1	0.210084034
BWAS	1	0.210084034
CART (Classification and Regression Trees) algorithm	1	0.210084034

CBAN	1	0.210084034
CCA+Pearson graph matching sparse group lasso	1	0.210084034
CFS reduction	1	0.210084034
COMPARE	1	0.210084034
Connections of interest (COIs)	1	0.210084034
Consensuse	1	0.210084034
Cosine algorithm	1	0.210084034
CRF-based dimension reduction algorithm	1	0.210084034
Dimension Reduction	1	0.210084034
Distance	1	0.210084034
DoG	1	0.210084034
Eeset	1	0.210084034
Elman Neural Network	1	0.210084034
EMB	1	0.210084034
Empirical mode decomposition	1	0.210084034
Ensemble feature selection	1	0.210084034
Extra-Trees	1	0.210084034
Factor-based feature extraction	1	0.210084034
FAE	1	0.210084034
FBM	1	0.210084034
FDR	1	0.210084034
Filter Bank Common Spatial Patterns	1	0.210084034
GABM	1	0.210084034
GAD	1	0.210084034
Graph-Based Feature Selection	1	0.210084034
Greedy	1	0.210084034
Grey level co-occurrence matrix	1	0.210084034
HOG	1	0.210084034
ICA-ICN	1	0.210084034
Information Gain	1	0.210084034
Inter-ICN	1	0.210084034
Kernel PCA	1	0.210084034
Kernel Principal Component Analysis	1	0.210084034
KW test	1	0.210084034
LAAM	1	0.210084034
latent variable	1	0.210084034
LBP-TOP	1	0.210084034
LICA	1	0.210084034
LLE	1	0.210084034
Low Frequency Components	1	0.210084034
MDA(mean decrease in accuracy)	1	0.210084034
MDS	1	0.210084034
Modified multiscale entropy	1	0.210084034
Motif configurations	1	0.210084034

mSVM-RFE	1	0.210084034
Multiple-task logistic regression	1	0.210084034
MVAR model	1	0.210084034
N2EN	1	0.210084034
Nested feature-selection	1	0.210084034
Network sparsity	1	0.210084034
NSD	1	0.210084034
Joint distribution adaptation (JDA) method	1	0.210084034
PDA	1	0.210084034
PDF-FS	1	0.210084034
peak	1	0.210084034
Pegosos	1	0.210084034
pLDA	1	0.210084034
PLI	1	0.210084034
PS assessment	1	0.210084034
RADACC, RADMPL, and AD(average degree)	1	0.210084034
Random forest–based feature selection	1	0.210084034
R-SFM	1	0.210084034
ReliefF	1	0.210084034
ResNet50	1	0.210084034
ROC	1	0.210084034
SBE	1	0.210084034
Sequential minimal optimization (SMO)	1	0.210084034
SFS	1	0.210084034
Short time series selection	1	0.210084034
SNV+PCA	1	0.210084034
Sparse logistic regression (SLR)	1	0.210084034
Stepwise analysis	1	0.210084034
Symmetrical Uncertainty (SU)	1	0.210084034
Task comparison	1	0.210084034
The multi scale ranked organizing maps (MS-ROM)	1	0.210084034
Top-k-feature-set	1	0.210084034
Units representing features	1	0.210084034
VGG16 model	1	0.210084034
Subject Weights	1	0.210084034

Supplementary Tab. 18 Summary for feature selection methods in existing studies. Almost methods were created originally, and thus provided self-defined name. More details for these methods can be found elsewhere in corresponding paper.

Neural modality (features)	No. of Studies	Proportion (%)
Functional MRI (rsFC)	158	33.19327731
Fusion (multiple neural modality)	101	21.21848739
EEG/ERP	99	20.79831933
Functional MRI (Task)	32	6.722689076
Structural MRI (DWI)	27	5.672268908
Structural MRI (GMV)	18	3.781512605
fNIRS	12	2.521008403
MEG	9	1.890756303
Functional MRI (Local features)	11	2.310924369
Functional MRI (PSD)	2	0.420168067
MRI (ASL)	4	0.084033613
SPECT	1	0.210084034

Supplementary Tab. 19 Summary for what neural features (modality) were used in existing studies. rsFC = resting-state functional connectivity; EEG = electroencephalogram/event-related potentials; DWI = diffusion weighted image; GMV = grey matter volumes; fNIRS = functional near-infrared spectroscopy; MEG = magnetoencephalogram; Local features = Reho, fALFF, ALFF and so on; PSD = power spectral density; ASL = arterial spin labeling; SPECT = single photon emission computed tomography.

Pre-processing methods	No. of Studies	Proportion (%)
None	278	58.40336134
Normalization	123	25.84033613
Fisher Z	52	10.92436975
Scaling	9	1.890756303
Standardization	3	0.630252101
L1 or L2	2	0.420168067
Regressed residual	2	0.420168067
0-1 Normalization	1	0.210084034
LeFMSF	1	0.210084034
Centering	1	0.210084034
GSP	1	0.210084034
HOG	1	0.210084034
Median	1	0.210084034
PLI	1	0.210084034

Supplementary Tab. 20 Summary for what pre-processing methods were used in existing studies. A portion of pre-processing methods were developed originally in corresponding studies, which can be found elsewhere for details.

Years	No. of open dataset	No. of studies	Proportion (%)
2011*	0	31	0
2012	8	29	27.5862069
2013	4	19	21.0526316
2014	6	28	21.4285714
2015	6	29	20.6896552
2016	10	29	34.4827586
2017	16	47	34.0425532
2018	21	44	47.7272727
2019	20	70	28.5714286
2020	36	99	36.3636364
2021**	28	51	54.9019608

Supplementary Tab. 21 Trends for the ratio of using open dataset on training ML models. * indicated the total counts during 1990 to 2011; ** represented this data is retrieved in July, 2021.

	Test	Statistics	Cohen d	95% CI	BF_{10}
Fold 1	Student	3.825***	0.663	0.31 - 1.01	115.29
	Mann-Whitney	3028***	0.370	0.18 - 0.52	444.10
Fold 2	Student	4.57***	0.793	0.40 - 1.14	1555.07
	Mann-Whitney	3234***	0.463	0.30 - 0.60	1423.78
Fold 3	Student	5.331***	0.924	0.56 - 1.28	31647.5
	Mann-Whitney	3616***	0.635	0.50 - 0.74	5087.73
Fold 4	Student	4.446***	0.854	0.45 - 1.24	881.52
	Mann-Whitney	2513***	0.655	0.51 - 0.76	1423.71

Supplementary Tab. 22 Results for comparison between SVM and DL classifiers on model performance. Results for Bayesian Mann-Whitney tests were based on augmentation algorithm with 5 chains of 1000 iteration. * $p < .05$, ** $p < .01$, *** $p < .001$

	Test	Statistics	Cohen d	95% CI	BF_{10}
Fold 1	Student	3.559***	0.610	0.26 - 0.95	50.19
	Mann-Whitney	3042***	0.320	0.13 - 0.48	19.18
Fold 2	Student	5.096***	0.874	0.52 - 1.23	12341
	Mann-Whitney	3806***	0.646	0.52 - 0.75	11156
Fold 3	Student	4.949***	0.849	0.49 - 1.20	6830
	Mann-Whitney	3845***	0.663	0.54 - 0.80	15187
Fold 4	Student	5.279***	0.905	0.55 - 1.25	26158
	Mann-Whitney	3995***	0.728	0.62 - 0.80	40171
Fold 5	Student	5.872***	1.007	0.65 - 1.36	340190
	Mann-Whitney	4388***	0.898	0.85 - 0.93	6.78×10^6
Fold 6	Student	4.927***	0.918	0.53 - 1.30	5475
	Mann-Whitney	3138***	0.846	0.77 - 0.96	41528

Supplementary Tab. 23 Results for comparison between external validation CV (leave-one-site-out CV and independent-samples (sites) CV) and others (i.e., k-fold, LOSO and hold-out CV) on model performance. Results for Bayesian Mann-Whitney tests were based on augmentation algorithm with 5 chains of 1000 iteration. * $p < .05$, ** $p < .01$, *** $p < .001$

Time	Item 1	Item 2	Item 3	Item 4	Item 5
2011	2.16129032	1.161290323	1.193548387	2.35483871	1.032258065
2012	2.58620689	2.24137931	1.275862069	2.448275862	1.206896552
2013	1.94736842	1.263157895	1.157894737	2.578947368	1
2014	2.21428571	1.571428571	1.285714286	2.571428571	1
2015	2.17241379	1.75862069	1.137931034	2.517241379	1.068965517
2016	2.62068965	1.931034483	1.275862069	2.413793103	1.137931034
2017	2.51063829	1.744680851	1.425531915	2.553191489	1
2018	2.88636363	2.454545455	1.818181818	2.727272727	1.363636364
2019	2.74285714	2.171428571	1.557142857	2.785714286	1.285714286
2020	2.94949494	2.444444444	1.525252525	2.616161616	1.262626263
2021	2.60784313	2.098039216	1.392156863	2.823529412	1.137254902
$r_{(rho)}$	0.76**	0.60	0.72*	0.78**	0.48
BF_{10}	5.01	2.70	3.53	7.03	1.28

Supplementary Tab. 24 Results for correlation between time and quality scores. Rho coefficient was estimated from Spearman correlation model. * $p < .05$, ** $p < .01$, *** $p < .001$, BF_{10} = Bayesian factor for supporting alternative hypothesis.

Category	Mean	S.D.	No. of studies
Anxiety Disorder	8.125	1.457737974	8
Bipolar Disorder	8.935483871	1.749961597	31
Depression Disorder	9	2.571556576	63
Conduct Disorder	8.333333333	1.505545305	6
Feeding and Eating Disorder	8.5	1	4
Neurodevelopment Disorder	10.52261307	3.842642347	199
Obsessive Compulsive Disorder	8.9375	2.619637379	143
Personality Disorder	9	1.414213562	2
Schizophrenia Disorder	9.854700855	2.853506486	117
Sleep Disorder	8	0	2
Somatic and Related Disorder	9	-	1
Substance-related and Addictive Disorder	10.15	2.680828623	20
Post-Traumatic Stress Disorder	9.142857143	0.690065559	7

Supplementary Tab. 25 Study quality across psychiatric category. S.D. = standard derivation
