# Supplementary material for: Sampling inequalities affect generalization of neuroimaging-based diagnostic classifiers in psychiatry
Source: BMC Med. 2023 Jul 3;21:241. doi: 10.1186/s12916-023-02941-4 (PMC10318841; doi:10.1186/s12916-023-02941-4)
Supplement: Supplementary file 2 — Additional file 2: Table S1. Evidence table. This table is to summary the metadata and metainformation of all the included studies. [file 12916_2023_2941_MOESM2_ESM.pdf]

ID	DSM category	First Author (s)	Publication year	Journal	Affiliation country	Sample country
[1]	Intellectual Disability	Inga Sophia Knoth	2018	Journal of Neurodevelopmental Disorders	Canada	Montreal
[2]	Intellectual Disability	Mangor Pedersen	2015	Epilepsia	Australia	Victoria
[3]	Intellectual Disability	Yalin Wang	2013	Neuroimage	USA	California
[4]	Intellectual Disability	Fumiko Hoeft	2011	JAMA Psychiatry	USA	California
[5]	Autism Spectrum Disorder	SarahParisot	2018	Medical Image Analysis	UK	multiple
[6]	Autism Spectrum Disorder	Sean Matlis	2015	BMC Neurology	USA	Massachusetts
[7]	Autism Spectrum Disorder	MadhuraIngalhalikar	2011	Neuroimage	USA	Pennsylvania
[8]	Autism Spectrum Disorder	HosseinShahamat	2020	Neural Networks	Iran	multiple
[9]	Autism Spectrum Disorder	Ghasem SadeghiBajestani	2019	Computer Methods and Programs in Biomedicine	Iran	Mashhad
[10]	Autism Spectrum Disorder	Pradyumna Lanka	2019	Brain imaging and behavior	USA	multiple
[11]	Autism Spectrum Disorder	LiangliangZhang	2020	Computer Methods and Programs in Biomedicine	China	Utah
[12]	Autism Spectrum Disorder	Mladen Rakić	2020	NeuroImage: Clinical	Spain	multiple
[13]	Autism Spectrum Disorder	Mehran Ahmadlou	2010	Journal of Clinical Neurophysiology	Iran	Tehran
[14]	Autism Spectrum Disorder	Lauren E. Libero	2015	Cortex	USA	Alabama

[15]	Autism Spectrum Disorder	The-Hanh Pham	2020	International journal of environmental research and public health	Singapore	Irbid
[16]	Autism Spectrum Disorder	Olfa Graa	2019	Journal of neuroscience methods	Turkey	Istanbul
[17]	Autism Spectrum Disorder	Madhura Ingalhalikar	2012	Medical image computing and computer-assisted intervention	USA	Pennsylvania
[18]	Autism Spectrum Disorder	Meenakshi Khosla	2019	NeuroImage	USA	multiple
[19]	Autism Spectrum Disorder	Hailong Li	2018	Front Neurosci	USA	multiple
[20]	Autism Spectrum Disorder	Bhaskar Sen	2018	Plos one	Canada	multiple
[21]	Autism Spectrum Disorder	Lingyu Xu	2020	Clinical Neurophysiology	China	GuangDong
[22]	Autism Spectrum Disorder	Xueke Ma	2021	Neuroscience Letters	China	Utah
[23]	Autism Spectrum Disorder	Zarina Rakhimberdina	2020	Sensors	Japan	multiple
[24]	Autism Spectrum Disorder	Vassilis Tsiaras	2011	Computers in Biology and Medicine	Greece	Heraklion
[25]	Autism Spectrum Disorder	Hui Wang	2012	Plos one	USA	California
[26]	Autism Spectrum Disorder	Jinlong Hu	2020	Computational and Mathematical Methods in Medicine	China	multiple
[27]	Autism Spectrum Disorder	Minyoung Jung	2019	Br J Psychiatry	Japan	Utah

[28]	Autism Spectrum Disorder	Anibal Sólon Heinsfeld	2017	NeuroImage: Clinical	Brazil	multiple
[29]	Autism Spectrum Disorder	Runa Bhaumik	2018	Neuroinformatics	USA	multiple
[30]	Autism Spectrum Disorder	Liye Wang	2016	Brain imaging and behavior	China	New York
[31]	Autism Spectrum Disorder	ChristineEcker	2010	NeuroImage	UK	London
[32]	Autism Spectrum Disorder	Seyedmehdi Payabvash	2019	Brain connectivity	USA	California
[33]	Autism Spectrum Disorder	True Price	2014	Medical image computing and computer-assisted intervention	USA	New York
[34]	Autism Spectrum Disorder	Reem Haweel	2020	Medical physics	USA	Kentucky
[35]	Autism Spectrum Disorder	Colleen P. Chen	2015	NeuroImage: Clinical	USA	multiple
[36]	Autism Spectrum Disorder	Jeffrey S. Anderson	2011	Brain	USA	Utah
[37]	Autism Spectrum Disorder	Lucina Q. Uddin	2014	JAMA Psychiatry	USA	California
[38]	Autism Spectrum Disorder	Jared A. Nielsen	2013	Frontiers in Human Neuroscience	USA	multiple
[39]	Autism Spectrum Disorder	Afrooz Jahedi	2017	Brain connectivity	USA	multiple
[40]	Autism Spectrum Disorder	Alessandra Retico	2016	Molecular Autism	Italy	Pisa

[41]	Autism Spectrum Disorder	Sara Calderoni	2012	Neuroimage	Italy	Pisa
[42]	Autism Spectrum Disorder	Bun Yamagata	2019	Brain imaging and behavior	Japan	Tokyo
[43]	Autism Spectrum Disorder	Ilaria Gori	2015	J Neuroimaging	Italy	Pisa
[44]	Autism Spectrum Disorder	Matthew Leming	2020	International Journal of Neural Systems	UK	multiple
[45]	Autism Spectrum Disorder	Mark D. Shen	2019	Lancet Psychiatry	USA	California
[46]	Autism Spectrum Disorder	EnzoGrossi	2017	Computer Methods and Programs in Biomedicine	Italy	Pisa
[47]	Autism Spectrum Disorder	Sukrit Gupta	2020	NeuroImage: Clinical	Singapore	New York
[48]	Autism Spectrum Disorder	Sina Ghiassian	2016	Plos one	Canada	New York
[49]	Autism Spectrum Disorder	Chen Zu	2019	Brain imaging and behavior	China	New York
[50]	Autism Spectrum Disorder	Gajendra J. Katuwal	2016	Plos one	USA	multiple
[51]	Autism Spectrum Disorder	Qin Li	2019	Cortex	China	multiple
[52]	Autism Spectrum Disorder	Omar Dekhil	2018	Plos one	USA	Kentucky
[53]	Autism Spectrum Disorder	Bun Yamagata	2019	Psychiatry and clinical neurosciences	Japan	Tokyo

[54]	Autism Spectrum Disorder	Tetsuyalidaka	2015	Cortex	Japan	multiple
[55]	Autism Spectrum Disorder	Wasifa Jamal	2014	J Neural Eng	UK	Southampton
[56]	Autism Spectrum Disorder	Fan Zhang	2018	Neuroimage	USA	Massachusetts
[57]	Autism Spectrum Disorder	Sheeba J Sujit	2019	J Magn Reson Imaging	USA	multiple
[58]	Autism Spectrum Disorder	Huifang Huang	2019	Human Brain Mapping	China	New York
[59]	Autism Spectrum Disorder	Aina Eill	2019	Brain connectivity	USA	California
[60]	Autism Spectrum Disorder	Xiang Xiao	2016	Autism Research	China	JiangSu
[61]	Autism Spectrum Disorder	Tae-Eui Kam	2017	Human Brain Mapping	Korea	multiple
[62]	Autism Spectrum Disorder	Sofia Ira Ktena	2018	Neuroimage	UK	multiple
[63]	Autism Spectrum Disorder	Maryam Akhavan Aghdam	2019	Journal of digital imaging	Iran	multiple
[64]	Autism Spectrum Disorder	Masoumeh Sadeghi	2017	Psychiatry Research: Neuroimaging	Iran	Utah
[65]	Autism Spectrum Disorder	Ahmad Chaddad	2017	BMC Neurosci	Canada	Pennsylvania
[66]	Autism Spectrum Disorder	Ridha Djemal	2017	BioMed research international	Saudi Arabia	Jeddah

[67]	Autism Spectrum Disorder	Noriaki Yahata	2016	Nature communication	Japan	Tokyo/New York
[68]	Autism Spectrum Disorder	T Heunis	2018	BMC medicine	South Africa	California
[69]	Autism Spectrum Disorder	Heng Chen	2015	Progress in Neuro-Psychopharmacology and Biology pathways	China	New York/California
[70]	Autism Spectrum Disorder	Marcel Adam Just	2014	Plos one	USA	Kentucky
[71]	Autism Spectrum Disorder	Maryam Akhavan Aghdam	2018	Journal of digital imaging	Iran	multiple
[72]	Autism Spectrum Disorder	Fahd A. Alturki	2020	Sensors	Saudi Arabia	Jeddah
[73]	Autism Spectrum Disorder	Alina Spiegel	2019	Current Biology	USA	Maryland
[74]	Autism Spectrum Disorder	Eugenia Conti	2020	Journal of personalized medicine	Italy	Pisa
[75]	Autism Spectrum Disorder	Xia-an Bi	2018	Frontiers in Human Neuroscience	China	multiple
[76]	Autism Spectrum Disorder	Luca Pollonini	2010	Annu Int Conf IEEE Eng Med Biol Soc	USA	Texas
[77]	Autism Spectrum Disorder	Justin Eldridge	2014	Journal of Neurodevelopmental Disorders	USA	Ohio
[78]	Autism Spectrum Disorder	Naseer Ahmed Khan	2020	Brain sciences	USA	multiple
[79]	Autism Spectrum Disorder	Zeinab Sherkatghanad	2020	Front Neurosci	Iran	multiple

[80]	Autism Spectrum Disorder	Jingjing Gao	2020	Front Neurosci	China	multiple
[81]	Autism Spectrum Disorder	Yaya Liu	2020	Experimental neurobiology	China	multiple
[82]	Autism Spectrum Disorder	Ming Yang	2021	Frontiers in Human Neuroscience	China	New York
[83]	Autism Spectrum Disorder	Zhi-An Huang	2020	IEEE Transactions on Neural Networks and Learning Systems	China	multiple
[84]	Autism Spectrum Disorder	Jie Zhao	2020	Brain and behavior	China	Beijing
[85]	Autism Spectrum Disorder	Fahad Almuqhim	2021	Frontiers in computational neuroscience	USA	multiple
[86]	Autism Spectrum Disorder	Lingyu Xu	2021	Clinical Neurophysiology	China	Shanghai
[87]	Autism Spectrum Disorder	James Lu	2015	Clinical psychological science	USA	Texas
[88]	Autism Spectrum Disorder	Md Rishad Ahmed	2020	IEEE J Biomed Health Inform	China	multiple
[89]	Autism Spectrum Disorder	Lingyu Xu	2019	Front Neurosci	China	Shanghai
[90]	Autism Spectrum Disorder	Chong‐Yaw Wee	2014	Human Brain Mapping	USA	Kentucky
[91]	Autism Spectrum Disorder	Harshini Sewani	2020	Children (Basel)	Canada	multiple
[92]	Autism Spectrum Disorder	Chunlei Shi	2021	Brain sciences	China	multiple

[93]	Autism Spectrum Disorder	Amirali Kazeminejad	2020	Front Neurosci	Canada	multiple
[94]	Autism Spectrum Disorder	WUTAO YIN	2019	JOURNAL OF COMPUTATIONAL BIOLOGY	Canada	multiple
[95]	Autism Spectrum Disorder	Yun Jiao	2010	Neuroimage	China	JiangSu
[96]	Autism Spectrum Disorder	Donna L. Murdaugh	2012	Plos one	USA	Alabama
[97]	Autism Spectrum Disorder	Yuqing Song	2019	Frontiers in Human Neuroscience	China	multiple
[98]	Autism Spectrum Disorder	Andrei Irimia	2018	Frontiers in computational neuroscience	USA	multiple
[99]	Autism Spectrum Disorder	Taban Eslami	2019	Front Neuroinform.	USA	multiple
[100]	Autism Spectrum Disorder	Darko Sarovic	2020	Int J Methods Psychiatr Res	Sweden	Gothenburg
[101]	Autism Spectrum Disorder	Amirali Kazeminejad	2019	Front Neurosci	Canada	multiple
[102]	Autism Spectrum Disorder	Xinyu Guo	2017	Front Neurosci	USA	Utah
[103]	Autism Spectrum Disorder	Rajat Mani Thomas	2020	Front Psychiatry	Netherlands	multiple
[104]	Attention-Deficit/Hyperactivity Disorder	He Chen	2019	Neuroscience	China	Beijing
[105]	Attention-Deficit/Hyperactivity Disorder	Xiaojie Guo	2020	Transl Psychiatry	China	Beijing

[106]	Attention-Deficit/Hyperactivity Disorder	Andreas Müller	2020	World J Biol Psychiatry	Switzerland	Chur
[107]	Attention-Deficit/Hyperactivity Disorder	Ming-Shan Gao	2020	Annu Int Conf IEEE Eng Med Biol Soc	Taiwan	multiple
[108]	Attention-Deficit/Hyperactivity Disorder	Ying Chen	2020	Artificial Intelligence in Medicine	China	multiple
[109]	Attention-Deficit/Hyperactivity Disorder	Muthuraman Muthuraman	2019	Scientific Report	Germany	Frankfurt am Main
[110]	Attention-Deficit/Hyperactivity Disorder	Chris McNorgan	2020	Front Physiol.	USA	multiple
[111]	Attention-Deficit/Hyperactivity Disorder	Amirali Vahid	2019	J Clin Med	Germany	Dresden
[112]	Attention-Deficit/Hyperactivity Disorder	Atif Riaz	2020	Journal of neuroscience methods	UK	multiple
[113]	Attention-Deficit/Hyperactivity Disorder	Mohammad Rostami	2020	Basic Clin Neurosci.	Iran	Tehran
[114]	Attention-Deficit/Hyperactivity Disorder	Hanni Kiiskia	2020	Clinical Neurophysiology	Ireland	Dublin
[115]	Attention-Deficit/Hyperactivity Disorder	Minyoung Jung	2020	Br J Psychiatry	Japan	multiple
[116]	Attention-Deficit/Hyperactivity Disorder	Yibin Tang	2019	Journal of Attention Disorders	China	multiple
[117]	Attention-Deficit/Hyperactivity Disorder	Yunkai Sun	2020	Neuropsychiatr Dis Treat.	China	New York
[118]	Attention-Deficit/Hyperactivity Disorder	Gagan Sidhu	2019	IEEE J Transl Eng Health Med	Canada	multiple

[119]	Attention-Deficit/Hyperactivity Disorder	Atif Riaz	2018	Computerized Medical Imaging and Graphics	UK	multiple
[120]	Attention-Deficit/Hyperactivity Disorder	Simranjit Kaur	2019	Clinical EEG and Neuroscience	India	Chandigarh
[121]	Attention-Deficit/Hyperactivity Disorder	Ming Chen	2019	Radiol Artif Intell	USA	multiple
[122]	Attention-Deficit/Hyperactivity Disorder	Stephanie Sutoko	2019	Neurophotonics	Japan	Tochigi
[123]	Attention-Deficit/Hyperactivity Disorder	Yuyang Luo	2020	NeuroImage: Clinical	USA	New Jersey
[124]	Attention-Deficit/Hyperactivity Disorder	Xun-Heng Wang	2018	Neuroscience Letters	China	New York
[125]	Attention-Deficit/Hyperactivity Disorder	Xiaolong Peng	2013	Plos one	China	Beijing
[126]	Attention-Deficit/Hyperactivity Disorder	Akira Yasumura	2020	Journal of Attention Disorders	Japan	Kodaira/Bunkyo/Setagaya/Fukushima/Saitama/Koganei/Fukuoka/Shinjuku/Tottori/Kofu
[127]	Attention-Deficit/Hyperactivity Disorder	Muhammad Naveed Iqbal Qureshi	2017	Frontiers in Human Neuroscience	Korea	multiple
[128]	Attention-Deficit/Hyperactivity Disorder	J Biederman	2017	Psychological Medicine	USA	Massachusetts
[129]	Attention-Deficit/Hyperactivity Disorder	Jean-G. Gehricke	2017	Plos one	USA	California
[130]	Attention-Deficit/Hyperact	João Ricardo Sato	2012	Front Syst Neurosci	Brazil	multiple

	ivity Disorder					
[131]	Attention-Deficit/Hyperactivity Disorder	Reto Iannaccone	2015	Eur Child Adolesc Psychiatry	Switzerland	Mannheim
[132]	Attention-Deficit/Hyperactivity Disorder	Yue Gu	2018	J Neural Eng	China	Beijing
[133]	Attention-Deficit/Hyperactivity Disorder	Junqiang Du	2016	Computerized Medical Imaging and Graphics	China	Beijing
[134]	Attention-Deficit/Hyperactivity Disorder	Xun-Heng Wang	2018	Scientific Report	China	multiple
[135]	Attention-Deficit/Hyperactivity Disorder	Heledd Hart	2014	Human Brain Mapping	UK	London
[136]	Attention-Deficit/Hyperactivity Disorder	Dai Dai	2012	Front Syst Neurosci	China	multiple
[137]	Attention-Deficit/Hyperactivity Disorder	Shuaiqi Liu	2021	IEEE TRANSACTIONS ON NEURAL SYSTEMS AND REHABILITATION ENGINEERING	China	multiple
[138]	Attention-Deficit/Hyperactivity Disorder	Xunheng Wang	2013	Eur J Radiol	China	New York
[139]	Attention-Deficit/Hyperactivity Disorder	Gagan S. Sidhu	2012	Front Syst Neurosci	Canada	multiple
[140]	Attention-Deficit/Hyperactivity Disorder	Martina D Liechti	2013	Brain Topogr	Switzerland	Zurich
[141]	Attention-Deficit/Hyperactivity Disorder	C Z Zhu	2005	NeuroImage	China	Beijing
[142]	Attention-Deficit/Hyperactivity Disorder	Emanuele Olivetti	2012	Front Syst Neurosci	Italy	multiple

[143]	Attention-Deficit/Hyperactivity Disorder	Blair A. Johnston	2014	Human Brain Mapping	UK	Juelich
[144]	Attention-Deficit/Hyperactivity Disorder	Abhay M S Aradhya	2018	2018 40th Annual International Conference of the IEEE Engineering in Medicine and Biology Society (EMBC)	Singapore	multiple
[145]	Attention-Deficit/Hyperactivity Disorder	Che-Wei Chang	2012	Front Syst Neurosci	Taiwan	multiple
[146]	Attention-Deficit/Hyperactivity Disorder	Janette L Smith	2003	Journal of Child Psychology and Psychiatry	Australia	Sydney
[147]	Attention-Deficit/Hyperactivity Disorder	Anderson dos Santos Siqueira	2014	BioMed research international	Brazil	multiple
[148]	Attention-Deficit/Hyperactivity Disorder	Huaiqiang Sun*,	2017	Radiology	China	Sichuan
[149]	Attention-Deficit/Hyperactivity Disorder	Andreas Mueller	2010	Nonlinear biomedical physics	Switzerland	Chur
[150]	Attention-Deficit/Hyperactivity Disorder	Wei Cheng	2012	Front Syst Neurosci	China	multiple
[151]	Attention-Deficit/Hyperactivity Disorder	Mehran Ahmadlou	2010	Clinical EEG and Neuroscience	Iran	Tehran
[152]	Attention-Deficit/Hyperactivity Disorder	Berdakh Abibullaev	2011	Journal of Medical Systems	Korea	Daegu
[153]	Attention-Deficit/Hyperactivity Disorder	John B. Colby	2012	Front Syst Neurosci	USA	multiple
[154]	Attention-Deficit/Hyperactivity Disorder	Andreas Mueller	2011	Nonlinear biomedical physics	Switzerland	Chur
[155]	Attention-Deficit/Hyperact	Dongchuan Yu	2013	Plos one	China	Beijing

	ivity Disorder					
[156]	Attention-Deficit/Hyperactivity Disorder	S-S Poil	2013	Clinical Neurophysiology	Switzerland	Zürich
[157]	Attention-Deficit/Hyperactivity Disorder	Muhammad Naveed Iqbal Quresh	2016	Plos one	Korea	multiple
[158]	Attention-Deficit/Hyperactivity Disorder	Sina Ghiassian	2016	Plos one	Canada	multiple
[159]	Attention-Deficit/Hyperactivity Disorder	Heledd Hart	2014	Journal of the American Academy of Child & Adolescent Psychiatry	UK	London
[160]	Attention-Deficit/Hyperactivity Disorder	Muhammad Naveed Iqbal Qureshi	2016	Annu Int Conf IEEE Eng Med Biol Soc	Korea	Beijing
[161]	Attention-Deficit/Hyperactivity Disorder	Gopikrishna Deshpande	2015	IEEE TRANSACTIONS ON CYBERNETICS	USA	multiple
[162]	Attention-Deficit/Hyperactivity Disorder	Rubi Hammer	2015	NeuroImage: Clinical	USA	Illinois
[163]	Attention-Deficit/Hyperactivity Disorder	MajidMoghaddari	2020	Computer Methods and Programs in Biomedicine	Iran	Tabriz
[164]	Attention-Deficit/Hyperactivity Disorder	Ernesto Pereda	2018	Plos one	Spain	Tenerife
[165]	Attention-Deficit/Hyperactivity Disorder	Lena Lim	2013	Plos one	UK	London
[166]	Attention-Deficit/Hyperactivity Disorder	HüseyinÖztoprak	2017	Clinical Neurophysiology	Cyprus	Ankara
[167]	Attention-Deficit/Hyperactivity Disorder	Hui TianTora	2021	Computer Methods and Programs in Biomedicine	Singapore	Clementi
[168]	Attention-Deficit/Hyperact	Mustafa Tosun	2021	Physical and Engineering Sciences in	Turkey	Kutahya

	ivity Disorder			Medicine		
[169]	Attention-Deficit/Hyperact ivity Disorder	Author links open overlay panelStuart J.Johnstone	2021	Biological Psychology	Australia	Beijing
[170]	Attention-Deficit/Hyperact ivity Disorder	Soumyabrata Dey	2012	Front Syst Neurosci	USA	multiple
[171]	Attention-Deficit/Hyperact ivity Disorder	Jae Hyun Yoo	2020	Brain imaging and behavior	Korea	Seoul
[172]	Attention-Deficit/Hyperact ivity Disorder	Aleksandar Tenev	2014	International Journal of Psychophysiology	Republic of Macedonia	Skopje
[173]	Attention-Deficit/Hyperact ivity Disorder	Bhaskar Sen	2018	Plos one	Canada	multiple
[174]	Attention-Deficit/Hyperact ivity Disorder	Maryam Rezaeezadeh	2020	Physical and Engineering Sciences in Medicine	Iran	Tehran
[175]	Attention-Deficit/Hyperact ivity Disorder	Alessandro Crippa	2017	Front Psychiatry	Italy	Lecco
[176]	Attention-Deficit/Hyperact ivity Disorder	Ayaka Ishii-Takahashi	2013	NeuroImage: Clinical	Japan	Tokyo
[177]	Attention-Deficit/Hyperact ivity Disorder	Tao Zhang	2020	Entropy	China	multiple
[178]	Attention-Deficit/Hyperact ivity Disorder	Dimitri M Abramov	2019	PeerJ	Brazil	Rio de Janeiro
[179]	Attention-Deficit/Hyperact ivity Disorder	Soumyabrata Dey	2014	Frontiers in neural circuits	USA	multiple
[180]	Attention-Deficit/Hyperact ivity Disorder	Halla Helgadóttir	2015	BMJ open	Iceland	Reykjavik

[181]	Attention-Deficit/Hyperactivity Disorder	Julie Chi Chow	2019	Clinical EEG and Neuroscience	Taiwan	Kaohsiung
[182]	Attention-Deficit/Hyperactivity Disorder	He Chen	2019	J Neural Eng	China	Beijing
[183]	Attention-Deficit/Hyperactivity Disorder	Hossein R. Jahanshahloo	2017	Journal of Medical Signals and Sensors	Iran	Once Caldas Deportiva
[184]	Attention-Deficit/Hyperactivity Disorder	Thomas Wolfers	2016	NeuroImage: Clinical	Netherlands	Amsterdam/Vrije
[185]	Attention-Deficit/Hyperactivity Disorder	Lizhen Shao	2020	Computer Methods and Programs in Biomedicine	China	Beijing
[186]	Attention-Deficit/Hyperactivity Disorder	Sarah Itani	2019	Plos one	Belgium	multiple
[187]	Attention-Deficit/Hyperactivity Disorder	Shiva Khoshnoud	2018	Journal of Integrative Neuroscience	Iran	Tehran
[188]	Attention-Deficit/Hyperactivity Disorder	Rupesh Kumar Chikara	2019	Sensors	Taiwan	Taibei
[189]	Specific Learning Disorder	Philipp Johannes Dinkel,	2013	Plos one	Germany	Aachen
[190]	Specific Learning Disorder	Sofia Zahia	2020	Computer Methods and Programs in Biomedicine	Spain	Bilbao
[191]	Specific Learning Disorder	Piotr Płoński	2017	Human Brain Mapping	Poland	Aachen
[192]	Specific Learning Disorder	Francisco J. Martinez-Murcia	2020	International Journal of Neural Systems	Spain	Malaga
[193]	Specific Learning Disorder	A Z A Zainuddin	2019	Annu Int Conf IEEE Eng Med Biol Soc	Malaysia	Selangor

[194]	Specific Learning Disorder	Bettina Serrallach	2016	Front Neurosci	Germany	Heidelberg
[195]	Specific Learning Disorder	Zaixu Cui	2016	Human Brain Mapping	China	Beijing
[196]	Specific Learning Disorder	Yolanda García Chimenoa	2014	Bio-Medical Materials and Engineering	Spain	Bilbao
[197]	Specific Learning Disorder	Stephen Bailey	2017	Ann Dyslexia	USA	Tennessee
[198]	Specific Learning Disorder	Opeyemi Lateef Usman,	2021	Plos one	Malaysia	Beijing
[199]	Specific Learning Disorder	Sara Mascheretti	2021	Brain sciences	Italy	Parini
[200]	Schizophrenia	Ang Li	2020	Nat Med	China	Beijing/Xian/ZhengZhou/Wuhan
[201]	Schizophrenia	Young Tak Jo	2020	Int J Methods Psychiatr Res	Korea	Seoul
[202]	Schizophrenia	Walid Yassin	2020	Transl Psychiatry	Japan	Tokyo
[203]	Schizophrenia	ZhiHong Chen	2020	Computational Intelligence and Neuroscience	China	multiple
[204]	Schizophrenia	V. Jahmunal	2019	Artificial Intelligence in Medicine	Singapore	Warsaw
[205]	Schizophrenia	Lubin Wang	2020	International Journal of Neural Systems	China	Beijing
[206]	Schizophrenia	Pavol Mikolas	2018	BMC Psychiatry	Germany	Prague
[207]	Schizophrenia	Emanuel Schwarz	2019	Transl Psychiatry	Germany	Aachen/Stockholm/Oslo/Verona
[208]	Schizophrenia	Mate Baradits	2020	Psychiatry Research	Hungary	Budapest
[209]	Schizophrenia	Jieun Kim	2020	Journal of neuroscience methods	Korea	Daejeon
[210]	Schizophrenia	Adriana Miyazaki de	2018	Psychiatry Research: Neuroimaging	Brazil	São Paulo

		Moura				
[211]	Schizophrenia	Maeri Yamamoto	2020	Plos one	Japan	Toyama/Nagoya
[212]	Schizophrenia	Hongliang Zou	2019	Artificial Intelligence in Medicine	China	unknown
[213]	Schizophrenia	Martin Rozycki	2018	Schizophrenia Bulletin	USA	Philadelphia/TianJing/Xian/Beijing
[214]	Schizophrenia	Sugai Liang	2020	Schizophrenia research	China	Sichuan
[215]	Schizophrenia	Golnoush Alamian	2020	NeuroImage: Clinical	Canada	Cardiff
[216]	Schizophrenia	A. de Pierrefeu	2018	Acta Psychiatrica Scandinavia	France	unknown
[217]	Schizophrenia	Pasquale Di Carlo	2020	Psychological Medicine	Italy	Bari
[218]	Schizophrenia	Youngoh Bae	2018	Journal of digital imaging	Korea	Washington
[219]	Schizophrenia	Yang Yu	2013	Plos one	China	Hunan
[220]	Schizophrenia	Petr Dluhos	2017	Neuroimage	Czech	Brno/Utrecht/Bohnice
[221]	Schizophrenia	Jungsun Lee	2018	NeuroImage: Clinical	USA	Seoul
[222]	Schizophrenia	Linda A Antonucci	2019	Neuropsychopharmacology	Germany	Bari
[223]	Schizophrenia	Xiaobing Lu	2016	Medicine (Baltimore)	China	GuangDong
[224]	Schizophrenia	Darya Chyzhyk	2015	International Journal of Neural Systems	Spain	San Sebastian
[225]	Schizophrenia	Rixing Jing	2019	Human Brain Mapping	China	Beijing
[226]	Schizophrenia	Minghui Hua	2020	Progress in Neuro-Psychopharmacology and Biology pathways	China	Tianjing
[227]	Schizophrenia	Ian C Gould	2014	NeuroImage: Clinical	Australia	multiple
[228]	Schizophrenia	Hong Song	2017	BMC Med inform decision making	China	Beijing
[229]	Schizophrenia	Qi Zhu	2018	Biomed Engineering Online	China	Nanjing
[230]	Schizophrenia	Wei Zhao	2020	Schizophrenia Bulletin	China	Taibei
[231]	Schizophrenia	Rowena Chin	2018	Scientific Report	Singapore	Buangkok View
[232]	Schizophrenia	S J Iwabuchi	2017	Psychological Medicine	UK	multiple

[233]	Schizophrenia	Julie L Winterburn	2019	Schizophrenia research	UK	multiple
[234]	Schizophrenia	Heng Chen	2017	Autism Research	China	Henan
[235]	Schizophrenia	Petra Verena Viher	2017	NeuroImage: Clinical	Switzerland	Sichuan
[236]	Schizophrenia	Ling-Li Zeng	2018	EBioMedicine	China	Xian/Anhui/Changsha/California/Washington
[237]	Schizophrenia	Kaustubh Supekar	2019	Biol Psychiatry	USA	New Mexico/Wisconsin
[238]	Schizophrenia	Dana Mastrovito	2018	NeuroImage: Clinical	USA	multiple
[239]	Schizophrenia	Yi Liu	2018	Schizophrenia research	China	Hunan/Henan
[240]	Schizophrenia	Long-Biao Cui	2018	Schizophrenia Bulletin	China	Shanxi
[241]	Schizophrenia	Walter H L Pinaya	2019	Human Brain Mapping	Brazil	unknown
[242]	Schizophrenia	Xi Chen	2017	Journal of affective disorders	China	Sichuan
[243]	Schizophrenia	Hugo G Schnack	2014	Neuroimage	Netherlands	Utrecht
[244]	Schizophrenia	Hu Cheng	2015	Schizophrenia research	USA	Indiana
[245]	Schizophrenia	P Mikolas	2016	Psychological Medicine	Czech	Prague
[246]	Schizophrenia	Archana Venkataraman	2012	Schizophrenia research	USA	Massachusetts
[247]	Schizophrenia	Shuai Wang	2018	Schizophrenia research	China	Henan
[248]	Schizophrenia	Carlos Cabral	2016	Schizophrenia Bulletin	Germany	multiple
[249]	Schizophrenia	Bjorn H Edrup	2019	Psychological Medicine	Denmark	Copenhagen
[250]	Schizophrenia	Walter H L Pinaya	2016	Scientific Report	Brazil	São Paulo
[251]	Schizophrenia	Junghoe Kim	2016	Neuroimage	Korea	multiple
[252]	Schizophrenia	Joseph de Viviano	2018	Biol Psychiatry	Canada	multiple
[253]	Schizophrenia	Eduardo Castro	2014	Annu Int Conf IEEE Eng Med Biol Soc	Chilie	unknown
[254]	Schizophrenia	Weigheng Yan	2019	EBioMedicine	China	Beijing/Xian/Xingxia

						ng/Wuhan/Zhumadian
[255]	Schizophrenia	Pierre Orban	2018	Schizophrenia research	Canada	unknown
[256]	Schizophrenia	Yang Yu	2013	Biomed Engineering Online	China	Hunan
[257]	Schizophrenia	Keith Dillon	2017	Journal of neuroscience methods	USA	multiple
[258]	Schizophrenia	Giulio Pergola	2017	Schizophrenia research	Italy	Bari
[259]	Schizophrenia	Hui Shen	2010	Neuroimage	China	Hunan
[260]	Schizophrenia	Wen-Lin Chu	2016	Behav Neurol	Taiwan	Chiayi
[261]	Schizophrenia	Kirill Masychev	2021	IEEE Trans Biomed Eng	USA	Ontario
[262]	Schizophrenia	A I Korda	2021	Psychiatry Research: Neuroimaging	Germany	Munich
[263]	Schizophrenia	Laura Pina-Camacho	2015	Eur Child Adolesc Psychiatry	Spain	multiple
[264]	Schizophrenia	Eduardo Castro	2011	Neuroimage	USA	Connecticut
[265]	Schizophrenia	Stefan P Koch	2015	Plos one	Germany	Berlin
[266]	Schizophrenia	Eduardo Castro	2014	Annu Int Conf IEEE Eng Med Biol Soc	Chilie	Massachusetts/Iowa/New Mexico
[267]	Schizophrenia	Juan I Arribas	2010	IEEE Trans Biomed Eng	Spain	San Sebastian
[268]	Schizophrenia	Jong H Yoon	2012	Schizophrenia research	USA	California
[269]	Schizophrenia	Darya Chyzhyk	2015	Neural Networks	Spain	multiple
[270]	Schizophrenia	Peng Wang	2008	Medical image computing and computer-assisted intervention	USA	unknown
[271]	Schizophrenia	Nuri F Ince	2008	Annu Int Conf IEEE Eng Med Biol Soc	USA	Minnesota
[272]	Schizophrenia	J R Sveinsson	1997	Med Eng Phys	Iceland	unknown
[273]	Schizophrenia	David Calhas	2020	Artificial Intelligence in Medicine	Portugal	Texas
[274]	Schizophrenia	Vince D Calhoun	2008	Human Brain Mapping	USA	Connecticut
[275]	Schizophrenia	Andres H Neuhaus	2012	European Archives of Psychiatry and	Germany	New York

				Clinical Neuroscience		
[276]	Schizophrenia	Tingting Xu	2016	IEEE TRANSACTIONS ON NEURAL SYSTEMS AND REHABILITATION ENGINEERING	USA	Minnesota
[277]	Schizophrenia	Maryam Ravan	2012	Annu Int Conf IEEE Eng Med Biol Soc	Canada	McMaster
[278]	Schizophrenia	U Castellani	2012	J Neural Transm (Vienna)	Italy	Verona
[279]	Schizophrenia	Smith K Khare	2020	BOOK	India	California
[280]	Schizophrenia	WeizhengYan	2021	Schizophrenia research	USA	multiple
[281]	Schizophrenia	Yuhui Du	2020	NeuroImage: Clinical	China	multiple
[282]	Schizophrenia	Kanghan Oh	2019	Schizophrenia research	Korea	Jeonju-si
[283]	Schizophrenia	Yingying Guo	2020	Brain sciences	China	multiple
[284]	Schizophrenia	Muhammad Naveed Iqbal Qureshi	2017	Front Neuroinform.	Korea	multiple
[285]	Schizophrenia	Ioannis K Gallos	2021	AIMS Neurosci	Greece	multiple
[286]	Schizophrenia	Tian Wang	2020	IEEE TRANSACTIONS ON CYBERNETICS	Singapore	multiple
[287]	Schizophrenia	Jinyu Zang	2021	Front Neurosci	China	GuangDong
[288]	Schizophrenia	Mengjiao Hu	2021	Schizophrenia research	Singapore	multiple
[289]	Schizophrenia	Kyungwon Kim	2021	Plos one	Korea	multiple
[290]	Schizophrenia	Raymond Salvador	2019	Front Neurosci	Spain	Barcelona
[291]	Schizophrenia	Po-Han Chou	2021	Front Psychiatry	Taiwan	Hsinchu
[292]	Schizophrenia	Zhuangzhuang Li	2021	Computerized Medical Imaging and Graphics	China	JiangSu
[293]	Schizophrenia	Honghui Yang	2010	Frontiers in Human Neuroscience	China	Connecticut
[294]	Schizophrenia	Sunil Vasu Kalmady	2019	NPJ Schizophr	Canada	Bangalore
[295]	Schizophrenia	Wenming Liu	2020	Front Neurosci	China	Shanxi
[296]	Schizophrenia	Ravi Bansal	2012	Plos one	USA	New York

[297]	Schizophrenia	Fali Li	2019	IEEE TRANSACTIONS ON NEURAL SYSTEMS AND REHABILITATION ENGINEERING	China	Beijing
[298]	Schizophrenia	Zongya Zhao	2021	Front Neurosci	China	Maryland
[299]	Schizophrenia	Su Mi Park	2021	Front Psychiatry	Korea	Seoul
[300]	Schizophrenia	Du Lei	2020	Human Brain Mapping	China	multiple
[301]	Schizophrenia	Jeong-Youn Kim	2020	J Clin Med	Korea	Seoul
[302]	Schizophrenia	Dafa Shi	2021	Disease Markers	China	multiple
[303]	Schizophrenia	Ahmad Shalbaf	2020	Physical and Engineering Sciences in Medicine	Iran	Warsaw
[304]	Schizophrenia	Eva Janousova	2016	Front Neurosci	Czech	Brno
[305]	Schizophrenia	Johannes Lieslehto	2021	NPJ Schizophr	Finland	multiple
[306]	Schizophrenia	Peng-Fei Ke	2021	Scientific Report	China	GuangDong
[307]	Schizophrenia	Mina Gheiratmand	2017	NPJ Schizophr	Canada	multiple
[308]	Schizophrenia	Jiayu Chen	2021	Human Brain Mapping	USA	multiple
[309]	Schizophrenia	Jihoon Oh	2020	Front Psychiatry	Korea	multiple
[310]	Schizophrenia	Kuldeep Singh	2021	Proc Inst Mech Eng H	India	Warsaw
[311]	Schizophrenia	Jie Sun	2021	Scientific Report	China	Beijing
[312]	Schizophrenia	Ying-Jie Li	2005	Conf Proc IEEE Eng Med Biol Soc	China	Shanghai
[313]	Schizophrenia	Jason K. Johannesen	2016	Neuropsychiatr Electrophysiol	USA	Connecticut
[314]	Schizophrenia	Kangyu Jin	2021	Acta Neuropsychiatr	China	Zhejiang
[315]	Schizophrenia	Siuly Siuly	2020	IEEE TRANSACTIONS ON NEURAL SYSTEMS AND REHABILITATION ENGINEERING	Australia	multiple
[316]	Schizophrenia	A I Korda	2021	Psychiatry Research: Neuroimaging	Germany	Munich

[317]	Bipolar Disorder	Hao LI	2020	BMC Psychiatry	China	GuangDong
[318]	Bipolar Disorder	Julia O Linke	2019	Journal of Child Psychology and Psychiatry	USA	Maryland
[319]	Bipolar Disorder	L.Squarcina	2019	Bipolar Disord	Italy	Verona
[320]	Bipolar Disorder	Koji Matsuo	2019	Cerebral Cortex	Japan	Yamaguchi/Hiroshima/Tokyo/TX
[321]	Bipolar Disorder	Sophia Frangou	2017	Neuroimage	UK	multiple
[322]	Bipolar Disorder	Nhat Trung Doan	2017	NeuroImage: Clinical	Norway	Oslo
[323]	Bipolar Disorder	Vince D. Calhoun	2008	Human Brain Mapping	USA	Connecticut
[324]	Bipolar Disorder	Eric A Reavis	2017	NeuroImage: Clinical	USA	California
[325]	Bipolar Disorder	Benson Mwangi	2015	Psychiatry Research	USA	North Carolina
[326]	Bipolar Disorder	Benson Mwangi	2017	Bipolar Disord	USA	Texas
[327]	Bipolar Disorder	Sergi G Costafreda	2011	BMC Psychiatry	UK	London
[328]	Bipolar Disorder	Barnaly Rashid	2016	Neuroimage	USA	Connecticut
[329]	Bipolar Disorder	Tobias Kaufmann	2017	Neuroimage	Norway	Oslo
[330]	Bipolar Disorder	Janaina Mourão-Miranda	2012	Bipolar Disord	UK	London
[331]	Bipolar Disorder	Mon-Ju Wu	2017	Neuroimage	USA	North Carolina
[332]	Bipolar Disorder	Christian Bürger	2017	Neuropsychopharmacology	Germany	Münster
[333]	Bipolar Disorder	Hugo G Schnack	2013	Neuroimage	Netherlands	multiple
[334]	Bipolar Disorder	Alan Anticevic	2014	Cerebral Cortex	USA	Connecticut
[335]	Bipolar Disorder	A Besga	2012	Neuroscience Letters	Spain	Vitoria
[336]	Bipolar Disorder	Juan I. Arribas	2010	IEEE Trans Biomed Eng	Spain	Connecticut
[337]	Bipolar Disorder	Ye Chen	2014	Journal of neuroscience methods	USA	unknown
[338]	Bipolar Disorder	Caixi Xi	2021	Journal of affective disorders	China	Zhejiang
[339]	Bipolar Disorder	Shin Teng	2013	Annu Int Conf IEEE Eng Med Biol Soc	Taiwan	Taipei

[340]	Bipolar Disorder	Yuhui Du	2020	NeuroImage: Clinical	USA	multiple
[341]	Bipolar Disorder	Ravi Bansal	2012	Plos one	USA	New York
[342]	Bipolar Disorder	Adeleh Dehghani Nazhvani	2013	Clin Neurol Neurosurg	Iran	Shiraz
[343]	Bipolar Disorder	Yen-Ling Chen	2020	Front Neurosci	Taiwan	Taipei
[344]	Bipolar Disorder	ZhuangzhuangLi	2021	Computerized Medical Imaging and Graphics	China	JiangSu
[345]	Bipolar Disorder	WeizhengYan	2021	Schizophrenia research	USA	multiple
[346]	Bipolar Disorder	Benson Mwangi	2017	Biol Psychiatry Cogn Neurosci Neuroimaging	USA	Texas
[347]	Bipolar Disorder	Su Mi Park	2021	Front Psychiatry	Korea	Seoul
[348]	Major Depressive Disorder	Ayumu Yamashita	2020	PLOS BIOL	Japan	Kyoto/Tokyo/Chiba
[349]	Major Depressive Disorder	Luigi A Maglanoc	2019	Human Brain Mapping	Norway	Oslo
[350]	Major Depressive Disorder	Miseon Shim	2019	NeuroImage: Clinical	USA	Gimhae-si
[351]	Major Depressive Disorder	Ji Ye Chun	2020	2020 42nd Annual International Conference of the IEEE Engineering in Medicine & Biology Society (EMBC)	USA	Sichuan/Henan/Zhejiang
[352]	Major Depressive Disorder	Caglar Uyulan	2021	Clinical EEG and Neuroscience	Turkey	Istanbul
[353]	Major Depressive Disorder	Yachen Shi	2021	ACS Chemical Neuroscience	China	multiple
[354]	Major Depressive Disorder	Shalini Mahato	2020	Journal of Medical Systems	India	Jharkhand

[355]	Major Depressive Disorder	Hao Guo	2012	Neuroreport	China	Shangxi
[356]	Major Depressive Disorder	Hao Guo	2019	Computational and mathematical methods in medicine	China	Shangxi
[357]	Major Depressive Disorder	Meenal J Patel	2015	International journal of geriatric psychiatry	USA	Pennsylvania
[358]	Major Depressive Disorder	Jie Yang	2018	Human Brain Mapping	USA	multiple
[359]	Major Depressive Disorder	Mehran Ahmadlou	2012	International Journal of Psychophysiology	Iran	Tehran
[360]	Major Depressive Disorder	Matthew D Sacchet	2015	Journal of Psychiatric Research	USA	California
[361]	Major Depressive Disorder	Rajamannar Ramasubbu	2019	Psychiatry and clinical neurosciences	Canada	Calgary, Alberta
[362]	Major Depressive Disorder	Hanxiaoran Li	2021	BMC Psychiatry	China	Zhejiang
[363]	Major Depressive Disorder	Xue Zhong	2017	Journal of affective disorders	China	Hunan
[364]	Major Depressive Disorder	Wenya Liu	2020	Clinical Neurophysiology	China	Niaoning
[365]	Major Depressive Disorder	Shih-Cheng Liao	2017	Sensors	China	Taibei
[366]	Major Depressive Disorder	Maria J Rosa	2015	Neuroimage	UK	London
[367]	Major Depressive Disorder	David M Schnyer	2017	Psychiatry Research: Neuroimaging	USA	Texas

[368]	Major Depressive Disorder	Blair A Johnston	2015	Plos one	UK	Dundee
[369]	Major Depressive Disorder	Feng Liu	2012	Plos one	China	Hunan
[370]	Major Depressive Disorder	Maobin Wei	2013	Psychiatry Research: Neuroimaging	China	JiangSu
[371]	Major Depressive Disorder	Shu-Hsien Chu	2018	2018 40th Annual International Conference of the IEEE Engineering in Medicine and Biology Society (EMBC)	USA	Minnesota
[372]	Major Depressive Disorder	Hao Guo	2017	Computational and mathematical methods in medicine	China	Shangxi
[373]	Major Depressive Disorder	Ling-Li Zeng	2014	Human Brain Mapping	China	Hunan
[374]	Major Depressive Disorder	Peng Fang	2012	Plos one	China	Hunan
[375]	Major Depressive Disorder	Wenjian Tan	2021	Australian & New Zealand Journal of Psychiatry	China	Hunan
[376]	Major Depressive Disorder	Longlong Cao	2014	Psychiatry and clinical neurosciences	China	Hunan
[377]	Major Depressive Disorder	Ling-Li Zeng	2012	Brain	China	Hunan
[378]	Major Depressive Disorder	Rajamannar Ramasubbu	2016	NeuroImage: Clinical	Canada	Calgary, Alberta
[379]	Major Depressive Disorder	Lihua Qiu	2014	J Neural Transm (Vienna)	China	Sichuan
[380]	Major Depressive	Benson Mwang	2012	Brain	UK	Aberdeen/Edinburg

	Disorder					h
[381]	Major Depressive Disorder	Jianlong Zhao	2020	Journal of neuroscience methods	China	Beijing/Xian/ZhengZhou/Wuhan
[382]	Major Depressive Disorder	Kun Bi	2016	Journal of affective disorders	China	JiangSu
[383]	Major Depressive Disorder	Xueling Zhu	2021	Brain imaging and behavior	China	Hunan
[384]	Major Depressive Disorder	A I Korda	2021	Psychiatry Research: Neuroimaging	Germany	Philadelphia
[385]	Major Depressive Disorder	Benedikt Sundermann	2017	J Neural Transm (Vienna)	Germany	multiple
[386]	Major Depressive Disorder	Jiaolong Qin	2014	Magnetic Resonance Imaging	China	JiangSu
[387]	Major Depressive Disorder	Ying-Jie Li	2002	Conf Proc IEEE Eng Med Biol Soc	China	Shanghai
[388]	Major Depressive Disorder	Miseon Shim	2019	NeuroImage: Clinical	USA	Goyang
[389]	Major Depressive Disorder	Man Guo	2020	J Neural Eng	China	Ningxia
[390]	Major Depressive Disorder	DANISH M. KHAN	2021	ieee access	Malaysia	Iskandar
[391]	Major Depressive Disorder	Bingtao Zhang	2021	IEEE TRANSACTIONS ON NEURAL SYSTEMS AND REHABILITATION ENGINEERING	China	Ningxia
[392]	Major Depressive Disorder	Yibo Zhu	2020	IEEE TRANSACTIONS ON NEURAL SYSTEMS AND REHABILITATION	USA	Texas

				ENGINEERING		
[393]	Major Depressive Disorder	Xiangfei Geng	2018	Front Neurosci	China	Tianjing
[394]	Major Depressive Disorder	Matthew D Sacchet	2014	Proc IEEE Int Symp Biomed Imaging .	USA	California
[395]	Major Depressive Disorder	Baoyu Yan	2020	Front Neurosci	China	Shanxi
[396]	Major Depressive Disorder	Takashi Nakano	2020	Front Psychiatry	Japan	Hiroshima
[397]	Major Depressive Disorder	Matthew D Sacchet	2015	Front Psychiatry	USA	California
[398]	Major Depressive Disorder	Yachen Shi	2021	Journal of affective disorders	China	JiangSu
[399]	Major Depressive Disorder	Lijuan Duan	2020	Frontiers in Human Neuroscience	China	Beijing
[400]	Major Depressive Disorder	Abdolkarim Saeedi	2021	Cognitive Neurodynamics	China	Iskandar
[401]	Major Depressive Disorder	Kun Bi	2018	Journal of affective disorders	China	JiangSu
[402]	Major Depressive Disorder	Jiaolong Qin	2015	Journal of affective disorders	China	JiangSu
[403]	Major Depressive Disorder	V Knott	2001	Psychiatry Research	Canada	Ontario
[404]	Major Depressive Disorder	Andre F Marquand	2008	Neuroreport	UK	London
[405]	Major Depressive	Qing Lu	2013	Brain research	China	JiangSu

	Disorder					
[406]	Major Depressive Disorder	R Cameron Craddock	2009	Magnetic Resonance Imaging	USA	Georgia
[407]	Major Depressive Disorder	Min Kang	2020	Sensors	Korea	Iskandar
[408]	Major Depressive Disorder	Giannis Lois	2016	Social cognitive and affective neuroscience	Germany	Mannheim
[409]	Major Depressive Disorder	Fatemeh Hasanzadeh	2020	Journal of affective disorders	Iran	Tehran
[410]	Major Depressive Disorder	Wajid Mumtaz	2017	Medical & Biological Engineering & Computing	Malaysia	Iskandar
[411]	Social Anxiety Disorder	Andreas Frick	2014	Behav Brain Res	Sweden	Uppsala
[412]	Social Anxiety Disorder	Wenjing Zhang	2015	BioMed research international	China	Sichuan
[413]	Social Anxiety Disorder	Feng Liu	2015	Brain Struct Funct	China	Sichuan
[414]	Social Anxiety Disorder	Mengqi Xing	2020	Front Psychiatry	USA	Illinois
[415]	Unspecified Anxiety Disorder	Mihai Gavrilescu	2019	Sensors	Romania	Bucharest
[416]	Unspecified Anxiety Disorder	Yunlong Xie	2020	Annu Int Conf IEEE Eng Med Biol Soc	China	Shanghai
[417]	Unspecified Anxiety Disorder	Su Mi Park	2021	Front Psychiatry	Korea	Seoul
[418]	General Anxiety Disorder	Jianping Qiao	2017	Frontiers in Human Neuroscience	China	Shandong
[419]	Obsessive-Compulsive Disorders	Xiaodan Xing	2020	Brain and behavior	China	Shenzhen
[420]	Obsessive-Compulsive Disorders	Xi Yang	2019	BMC Psychiatry	China	Sichuan

[421]	Obsessive-Compulsive Disorders	Willem B. Bruin	2020	Transl Psychiatry	Netherlands	multiple
[422]	Obsessive-Compulsive Disorders	Yu Takagi	2017	Scientific Report	Japan	Kyoto
[423]	Obsessive-Compulsive Disorders	Cong Zhou	2018	Front Psychiatry	China	Yunnan
[424]	Obsessive-Compulsive Disorders	Xinyu Hu	2016	Eur Neuropsychopharmacol	China	Sichuan
[425]	Obsessive-Compulsive Disorders	Xuan Bu	2019	Transl Psychiatry	China	Sichuan
[426]	Obsessive-Compulsive Disorders	Bhaskar Sen	2016	Annu Int Conf IEEE Eng Med Biol Soc	USA	Minnesota
[427]	Obsessive-Compulsive Disorders	Lucas R Trambaiolli	2017	Journal of affective disorders	Brazil	Santo André
[428]	Obsessive-Compulsive Disorders	Fei Li	2014	Human Brain Mapping	China	Sichuan
[429]	Obsessive-Compulsive Disorders	Sona Khaneh Shenas	2014	Annu Int Conf IEEE Eng Med Biol Soc	Turkey	Ankara
[430]	Obsessive-Compulsive Disorders	Jing Liu	2021	Human Brain Mapping	China	Sichuan
[431]	Obsessive-Compulsive Disorders	Wei Liu	2020	Brain imaging and behavior	China	Tianjing
[432]	Obsessive-Compulsive Disorders	Yunhui Chen	2021	Neural Plast	China	Heilongjiang
[433]	Obsessive-Compulsive Disorders	I Kalatzis	2005	Conf Proc IEEE Eng Med Biol Soc	Greece	Athens

[434]	Obsessive-Compulsive Disorders	Serap Aydin	2015	International Journal of Neural Systems	Turkey	Istanbul
[435]	Posttraumatic Stress Disorder	Sigal Zilcha-Mano	2020	Biol Psychiatry Cogn Neurosci Neuroimaging	Israel	New York
[436]	Posttraumatic Stress Disorder	Andrew A Nicholson	2019	Psychological Medicine	UK	Ontario
[437]	Posttraumatic Stress Disorder	H Zhu	2020	Acta Psychiatrica Scandinavia	China	Sichuan
[438]	Posttraumatic Stress Disorder	Miseon Shim	2019	NeuroImage: Clinical	USA	Goyang
[439]	Posttraumatic Stress Disorder	Andrew A Nicholson	2020	NeuroImage: Clinical	Canada	Ontario
[440]	Posttraumatic Stress Disorder	Sherain Harricharan	2020	Psychophysiology	Canada	Ontario
[441]	Posttraumatic Stress Disorder	Su Mi Park	2021	Front Psychiatry	Korea	Seoul
[442]	Somatic Symptom Disorder	Aykut Eken	2019	J Neural Eng	Spain	Barcelona
[443]	Anorexia Nervosa	Lavagnino L	2015	Psychological Medicine	USA	Turin
[444]	Anorexia Nervosa	Luca Lavagnino	2018	Int J Eat Disord.	USA	Colorado
[445]	Anorexia Nervosa	Daniel Geisler	2018	Psychological Medicine	Germany	Magdeburg
[446]	Bing-Eating Disorder	Martin Weygandt	2012	Human Brain Mapping	Germany	Berlin
[447]	Insomnia Disorder	Mi Hyun Lee	2021	Scientific Report	Korea	Inchon
[448]	Insomnia Disorder	Christoph Jansen	2019	Chaos	Germany	Berlin
[449]	Conduct Disorder	Jiang Zhang	2020	Medical & Biological Engineering & Computing	China	Hunan

[450]	Conduct Disorder	Jianing Zhang	2019	Brain imaging and behavior	China	Hunan
[451]	Conduct Disorder	Jianing Zhang	2018	Frontiers in Human Neuroscience	China	Hunan
[452]	Antisocial Personality Disorder	Yan Tang	2013	Plos one	China	Hunan
[453]	Antisocial Personality Disorder	Yan Tang	2013	Neuroreport	China	Hunan
[454]	Antisocial Personality Disorder	João R Sato	2012	Social Neuroscience	Brazil	Rio de Janeiro
[455]	Other (or Unknown) Substance Use Disorder	Reagan R. Wetherill	2019	Addict Biol	USA	Pennsylvania
[456]	Other (or Unknown) Substance Use Disorder	Yadi Li	2019	Addict Biol	China	Fujian
[457]	Other (or Unknown) Substance Use Disorder	Xinfang Ding	2020	Brain and behavior	China	Shandong
[458]	Other (or Unknown) Substance Use Disorder	Mutlu Mete	2016	BMC Bioinformatics	USA	Texas
[459]	Alcohol Use Disorder	Ehsan Adeli	2020	Biol Psychiatry Cogn Neurosci Neuroimaging	Canada	California
[460]	Alcohol Use Disorder	Matthias Guggenmos	2020	Scientific Report	Germany	Berlin
[461]	Alcohol Use Disorder	Virender Kumar Mehla	2020	Journal of neuroscience methods	India	Illinois
[462]	Alcohol Use Disorder	Xi Zhu	2019	Neuroscience Letters	USA	Maryland/New York
[463]	Alcohol Use Disorder	Wajid Mumtaz	2018	Artificial Intelligence in Medicine	Malaysia	Sabah
[464]	Alcohol Use Disorder	Shui-Hua Wang	2017	Journal of Medical Systems	China	JiangSu
[465]	Alcohol Use Disorder	Sivan Kinreich	2021	Transl Psychiatry	USA	multiple

[466]	Alcohol Use Disorder	Youngoh Bae	2017	Physiol Meas	Korea	Illinois
[467]	Alcohol Use Disorder	Surendra Kumar	2015	Medical & Biological Engineering & Computing	India	Jharkhand
[468]	Alcohol Use Disorder	Danish M Khan	2021	IEEE TRANSACTIONS ON NEURAL SYSTEMS AND REHABILITATION ENGINEERING	Malaysia	Seri Iskandar
[469]	Alcohol Use Disorder	Wajid Mumtaz	2017	Cognitive Neurodynamics	Malaysia	Sabah, Bingkor
[470]	Alcohol Use Disorder	Sage Hahn	2020	Human Brain Mapping	USA	multiple
[471]	Alcohol Use Disorder	Hongyi Zhang	2020	Frontiers in Human Neuroscience	China	Illinois
[472]	Alcohol Use Disorder	Shui-Hua Wang	2019	Front Psychiatry	China	Henan
[473]	Alcohol Use Disorder	Sunil Kumar Prabhakar	2020	Heliyon	Korea	Illinois
[474]	Opioid Use Disorder	Turker Tekin Erguzel	2019	Clinical EEG and Neuroscience	Turkey	Istanbul
[475]	Borderline Personality Disorder	Henk Cremers	2020	European Archives of Psychiatry and Clinical Neuroscience	Netherlands	Maastricht
[476]	Borderline Personality Disorder	Tingting Xu	2014	2014 36th Annual International Conference of the IEEE Engineering in Medicine and Biology Society	USA	Minnesota

Table S1. Evidence table. This table is to summary the metadata and metainformation of all the included studies (k = 476).
