# Supplementary material for: Sampling inequalities affect generalization of neuroimaging-based diagnostic classifiers in psychiatry
Source: BMC Med. 2023 Jul 3;21:241. doi: 10.1186/s12916-023-02941-4 (PMC10318841; doi:10.1186/s12916-023-02941-4)
Supplement: Supplementary file 3 — Additional file 3: Table S1-S2. TabS1. RNIMP 2022 Checklist. Reporting guideline and checklist for neuroimaging-based machine learning studies for psychiatry. TabS2. RNIMP 2022 workflow diagram. A workflow to guide for reporting neuroimaging-based machine learning models for psychiatry. [file 12916_2023_2941_MOESM3_ESM.pdf]

RNIMP 2022 Checklist for Reporting guideline for neuroimaging-based machine learning studies for psychiatry

Section and topics	Item #	Description	Page for reporting location
**PRE-REGISTRATION**			
Pre-register	0	Reporting pre-registration platform and valid hyperlink to access it	
**TITLE AND ABSTRACT**			
Title	1	Identifying study as machine-learning model aiming at psychiatric diagnoses	
Abstract	2	Structurally reporting background, methods (internal and external sample size, model, cross-validation, at least), results (AUC, accuracy, sensitivity and specificity) and conclusions	
**INTRODUCTION**			
Introduction	3	Describing rationale for clinical and scientific implications	
	4	Clarifying research proposal and hypotheses as reported in pre-registration	
**METHODS**			
Research sample	5	Identifying what sample population is for this study, such as different sites (multiple sites with different population would be recommended)	
Sampling strategy	6	Detailing how to determine the sample size	
Data collection	7	Reporting how to recruit participants or indicating what OA dataset is used	
Data timing	8	Reporting data collection date or duration	
Inclusion criteria	9a	Detailing how to include patients by clinical criteria, such as DSM-5 or ICD-10	
	9b	Detailing how to include healthy control, and reporting the case/control ratio. If the ratio is at high risk of skewness, it would be reported for how to re-sample dataset	
Exclusion criteria	10a	Reporting how to exclude participants by clinical criteria, such as comorbidity and symptom	
	10b	Reporting how to remove participants as technical flaws, such as head-motion or artifact	
**DATA ANALYSIS**			
Technical details	11a	Reporting adequate technical details for collecting data, such as scanner, task, equipment, scanning parameters, and neuroimaging modality, at least	

Quality control	11b	Reporting how to perform quality control	
Preprocessing	11c	Reporting how to preprocess raw neuroimaging data and detailing how to preprocess features preceding to build ML model, such as Fish's z transformation or 0-1 normalization (preprocessing feature vectors preceding to train model would be recommended)	
**MODEL SETTINGS**			
Model	12a	Reporting what model is built, such as SVM, GPC or RNN	
Toolkit	12b	Reporting what toolbox, software or self-made codes are used for building ML model	
Feature selection	12c	Reporting how to do feature selection and preparation (if applicable), and validating no leakage of testing data in selecting features	
Parameters	12d	Reporting hwo to perform parameter selection (if applicable), such as kernel function or penalty function; clarifying what parameters are used in final ML model	
Cross-validation	12e	Reporting what cross-validation scheme is adopted for estimating model performance (k-fold cross-validation portfolio would be recommended)	
External sample	12f	Reporting details of independently external sample(s) for validation and generalizability (if applicable)(External sample for validation would be recommended)	
Control analysis	13	Testing trained model by other sample splits or other populations (if applicable)	
Robustness analysis	14	Testing model performance by different parameters for validating model robustness (if applicable)	
Interpretation	15	Reporting feature contributions or anything else facilitating to improve model interpretation (if applicable)	
**RESULTS**			
Diagram	16	Reporting the workflow diagram (see RNIMP 2022 Workflow Diagram)	
Sample details	17	Reporting final sample characteristics	
Model performance	18	Reporting mandatory measures for model performance, including accuracy, sensitivity, specificity, AUC, ROC plot and confusion matrix for inner and external samples (if applicable)	
Statistics	19	Reporting how to make statistical inference, such as bootstrap or permutation test	

Interpretation	20	Interpreting how this model could perform well to predict patients	
**DISCUSSION**			
Model advance	21	Discussing how to achieve high model performance and major advance this model delivers	
Interpretation	22	Discussing how to explain this ML model by decoding feature contributions or patterns	
Clarification	23	Discussing why methods or parameters are used inconsistently as reported in pre-registration (if applicable)	
Implication	24	Discussing the clinical and scientific values for this model	
Limitation	25	Discussing what limitations this model possesses and directing future research	
**ADDITIONAL INFORMATION**			
Model availability	26	Reporting how to get access for trained model, such as OSF or Github (if applicable)	
Code availability	27	Reporting how to obtain codes or toolboxes to reproduce this study adequately (if applicable)	
Data availability	28	Reporting how to gain raw data or materials (if applicable)	

Tab. S1 RNIMP 2022 Checklist. Reporting guideline and checklist for neuroimaging-based machine learning studies for psychiatry.

RNIMP 2022 workflow diagram for Reporting guideline for neuroimaging-based machine learning studies for psychiatry

Pre-registered site:

Prepared eligible subjects: $n_{patients}$ = ; $n_{control}$ =

Exclusion criteria by clinical diagnose

Excluded ($n_{patients}$ = ; $n_{control}$ =)

Reason 1 :

Reason 2 :

Reason 3:

Eligible subjects: $n_{patients}$ = ; $n_{control}$ =

Exclusion criteria by data quality control

Excluded ($n_{patients}$ = ; $n_{control}$ =)

Reason 1 :

Reason 2 :

Reason 3:

Eligible subjects: $n_{patients}$ = ; $n_{control}$ =

Upper layers report parametric details in line with pre-registration; Bottom layers report what actually adopted in building model

Toolkit:

Toolkit:

Feature select:

Feature select:

External sample:

$n_{patient}$ =

$n_{control}$ =

External sample:

$n_{patient}$ =

$n_{control}$ =

Cross-validation:

Cross-validation:

Reasons should be given if there are inconsistent model parameter details between preregistration and actual usages

Reason 1 :

Reason 2 :

Reason 3:

....

....

TP: (%)	FP: (%)
FN: (%)	TN: (%)

(paste ROC curve here)

ROC curve plot should be inserted in the left box; if multiple models are built, please integrate curves into one ROC plot

Tab. S2 RNIMP 2022 workflow diagram. A workflow to guide for reporting neuroimaging-based machine learning models for psychiatry.
